# C-2 functionalization of indoles with xanthate-lactam derivatives by radical-oxidative coupling, an approach to *Aspidosperma* alkaloids†

**DOI:** 10.1039/d5ra01600b

**Published:** 2025-07-18

**Authors:** Manuel Pastrana, Luis D. Miranda

**Affiliations:** a Instituto de Química, Universidad Nacional Autónoma de México, Circuito Exterior, Ciudad Universitaria Coyoacán Mexico City 04510 Mexico lmiranda@unam.mx

## Abstract

The C-2 functionalization of various indole derivatives *via* radical-oxidative coupling is presented. The xanthate precursors utilized as alkylating agents are derived from two distinct lactams, which are of considerable significance due to their presence in numerous monoterpenoid indole alkaloids, such as *Aspidosperma* and ibophyllidine types. The synthesis of 1,2-dehydroaspidospermidine exemplifies the application of this methodology.

## Abstract 2

Key intermediates in the total synthesis of indole-monoterpene alkaloids were obtained from a C2-radical alkylation of indoles using lactam-derived xanthates.

## Graphical abstract

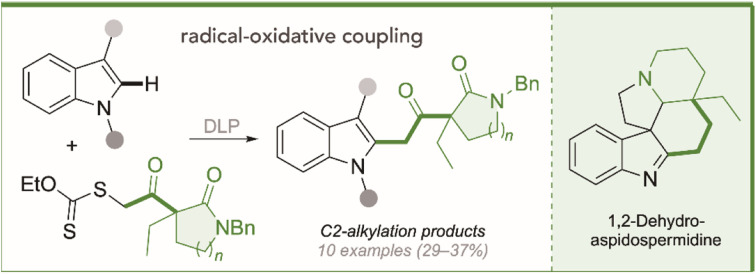

The importance of indole-monoterpene alkaloids lies in their formidable diversity of complex polycyclic structures and their prominent biological activities.^1^ Molecules representative of these natural substances that show notable differences in their structural topology include aspidospermidine (1), vincadifformine (2), goniomitine (3), quebrachamine (4), ibophyllidine (5), and desethylibophyllidine (6). These molecules have been isolated from different natural sources and have been shown to exhibit important biological effects. Thus, based on the possibility of their pharmacological usefulness, developing new synthetic alternatives of these compounds that give access to the natural scaffolds and their analogs is worth investigating. Within this field of exploration, the synthetic chemistry community has focused on the concept of divergent alternatives or unified strategies, which enable the synthesis of various natural or unnatural products from common precursors.^2^

Interestingly, a close analysis revealed that although alkaloids 1–6 appear to have topologically different molecular structures, a common structural motif can be identified (Scheme 1). There is a piperidine ring or, in the cases of 5 and 6, a pyrrolidine ring connected by a two-carbon bridge to the C-2 position of the indole. Based on this observation in 2018, we reported a unified synthetic approach to five topologically diverse *Aspidosperma* alkaloids using the indole δ-valerolactam (8) as a common intermediate.^3^ The approach relied on the chemo-differentiated trapping process of the iminium formed during the hemi-reduction of the lactam A or *via* its over-reduction in the case of the synthesis of quebrachamine (4). In this sense, different methodologies have previously been used to obtain intermediates of type B, generally through a low-yielding multi-step synthetic sequence.^4^ Indeed, our approach for synthesizing A took seven steps, with the *de novo* synthesis of the indole system.

**Scheme 1:**
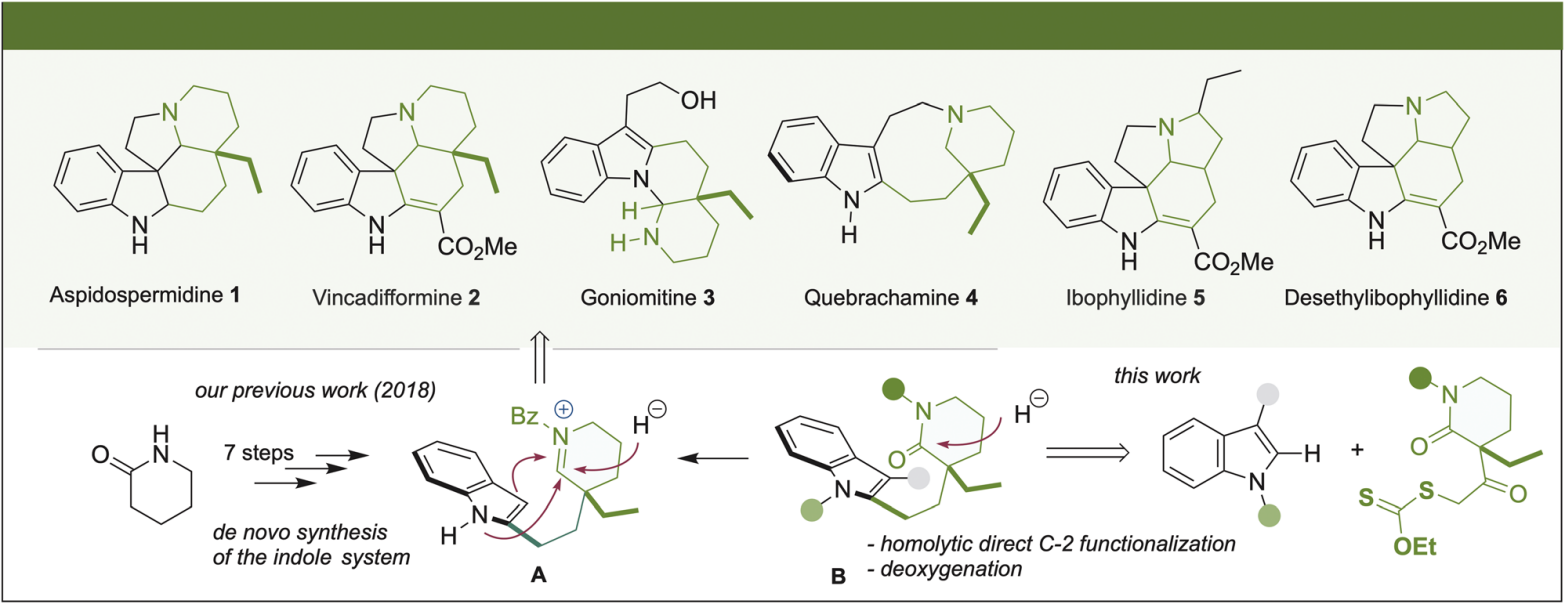
Important advanced intermediates derived from C-2 alkylation over indole.

Building upon these antecedents and our longstanding interests in the C-2 homolytic alkylation of indoles, we recently envisioned connecting the indole system directly with the ethyl-valerolactam fragment through a radical-oxidative coupling reaction. In this context, homolytic direct functionalization of indoles represents a notable advancement by facilitating alkylation at the C-2 position.^5^ Notably, over the years, we have demonstrated the efficiency of radical xanthate-based chemistry for carrying out the homolytic alkylation of several heterocyclic systems, including several indole derivatives.^6^

This study reports the radical-oxidative coupling at the C-2 position of synthetically relevant indole derivatives with two xanthates derived from 2-pyrrolidone (7) and δ-valerolactam (8). In principle, alkylated indole products possess structural features that can be considered valuable intermediates for progressing to natural products 1–6 or analogs. They have a piperidone ring, or a pyrrolidine ring, connected by a two-carbon bridge to the C-2 position of the indole. We envisaged that B could be accessed through a Barton–McCombie deoxygenation of the secondary alcohol obtained by reducing the ketone fragment. Then, the tetracyclic system in 1–4 might be constructed by some variant of the already reported intramolecular Bischler–Napieralski condensation. Furthermore, the methodology might be useful to obtain valuable intermediates in synthesizing ibophyllidine alkaloids 5 and 6, starting from the precursor 2-pyrrolidone xanthate.

Xanthates 17 and 18 were prepared from commercially available 2-pyrrolidone (7) and δ-valerolactam (8) in five steps (Scheme 2). The protection of the nitrogen with benzyl bromide (BnBr) produced the *N*-benzyl lactams 9 and 10, and subsequent α-acylation of the carbonyl group by methyl acetate (MeOAc), mediated by the *in situ* synthesis of lithium diisopropylamide (LDA), yielded the 1,3-ketoamides 11 and 12. Then, alkylation of the 1,3-dicarbonyl system was conducted using K_2_CO_3_ and iodoethane, forming *rac*-13 and *rac*-14. The formation of bromides 15 and 16 occurred through the generation of the corresponding sodium enolate, which was subsequently trapped by chlorotrimethylsilane (TMSCl), followed by the bromination of the respective silyl enol ether. Finally, the displacement of the bromine by potassium ethyl xanthogenate (KSCSOEt) gave the xanthates 17 and 18. This protocol allowed the xanthate synthesis on a multigram scale with good yields at each stage.

**Scheme 2:**
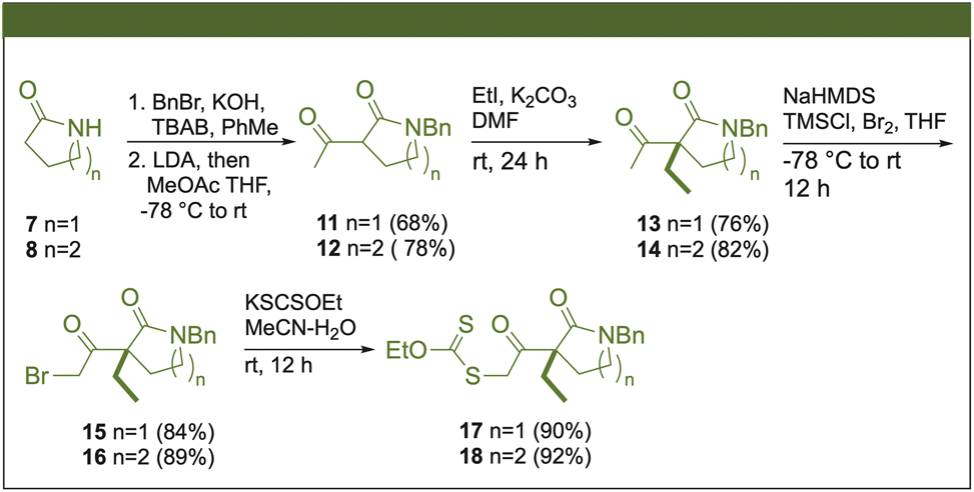
Synthesis of the xanthate intermediates.

The next stage involved the oxidative radical coupling process between compounds 17 or 18 with various indole derivatives carrying different substituents at C-3 (19a–e) (Scheme 3). These indole derivatives were chosen based on the commercially available indoles used in synthesizing several indole-monoterpene alkaloids. Compounds 20 and 25 exhibited slightly higher yields (37–38%), while others did not surpass a yield of 33%. In this context, the presence of substituents at C-3, despite stabilizing the benzyl radical formed after addition at C-2 might create steric hindrances in the addition step of the radical formed from the xanthates. The radical coupling with indole derivative 19d, having an electron-withdrawing group at C-3, demonstrated that xanthates 17 and 18, precursors of electrophilic radicals stabilized by a carbonyl group, could react with indoles systems with electrophilic behavior. Thus, whether the group at C-3 on the indole was electron-donating or electron-withdrawing had little effect on the outcome of the radical yields. Besides the expected product, we observed the complete consumption of the xanthate and the indole substrate's excess. The remaining byproducts form an inseparable mixture with no significant components. This problem may be due to a reaction between the xanthate and the stoichiometric amount of dilauroyl peroxide. Even though yields are moderate, this process enables the generation of intermediates 20–29, potential building blocks for the synthesis of *Aspidosperma* and ibophyllidine alkaloids.

**Scheme 3:**
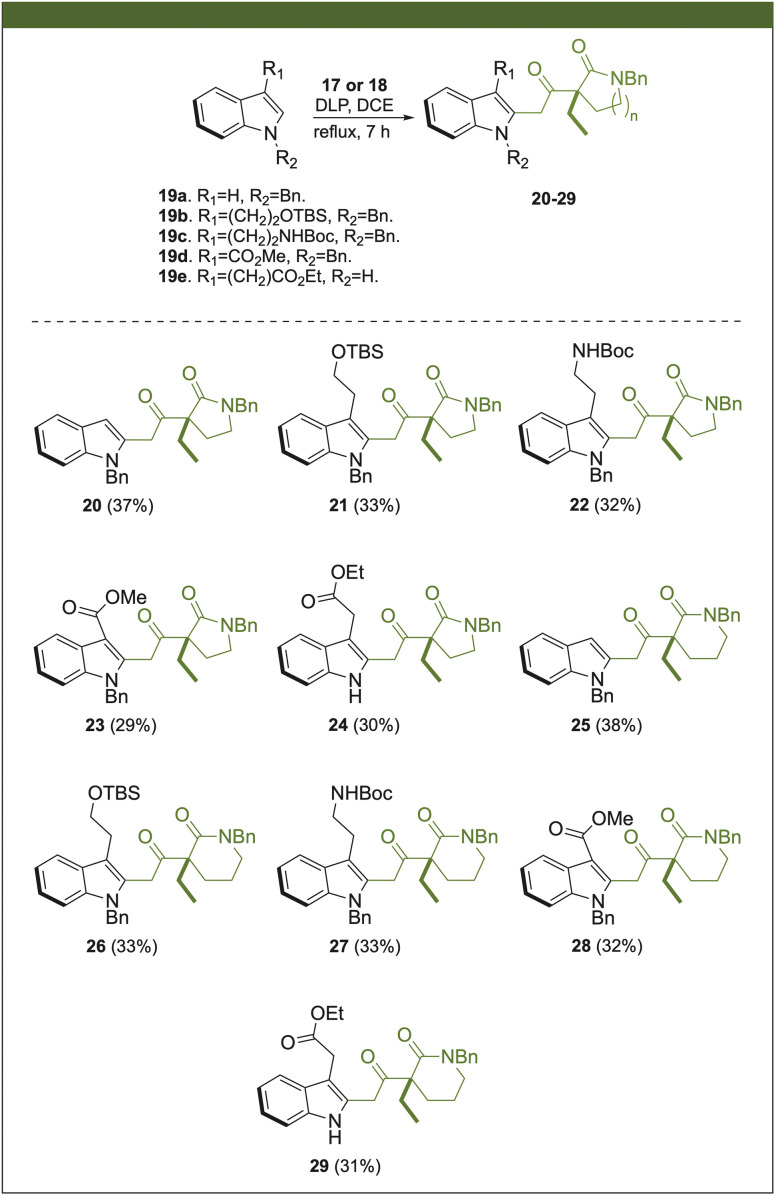
Alkylation at C-2 position of indole derivatives by a radical-oxidative coupling.

In the next stage, we chose 20, 25, and 26 as representative examples to apply the protocol for deoxygenation of the ketone group (Scheme 4). The reduction of the keto groups was initially carried out using NaBH_4_. Then, without thoroughly purifying the secondary alcohols, the formation of xanthates 30–32 was performed using the classic CS_2_ and methyl iodide protocol under basic conditions. Thus, the respective diastereoisomeric pairs were obtained for all xanthates, which were purified for their corresponding characterizations, but in the syntheses were used as a mixture in the next reaction step. The Barton–McCombie deoxygenation was carried out using *n*-Bu_3_SnH as a reducing agent in the presence of azobisisobutyronitrile (AIBN) with yields above 88% to get intermediates 33–35. The double *N*-debenzylation at the indole and lactam moieties was carried out in one step by sodium in ammonia at −78 °C, giving the desired products 36–38. It is noteworthy that intermediate 38 represents an interesting intermediate that could provide access to goniomitine^7^ and quebrachamine^8^ alkaloids.

**Scheme 4:**
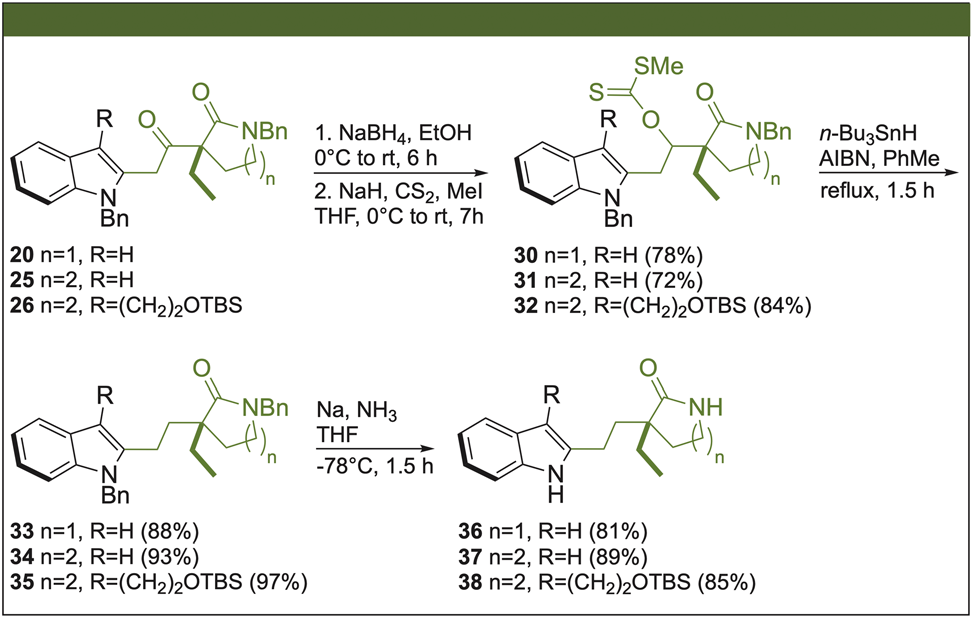
Barton–McCombie deoxygenation protocol.

The synthesis of 1,2-dehydroaspidospermidine (42) was performed using our previously reported strategy starting from the advanced intermediate 37 (Scheme 5).^3^ Thus, protection of the *N*-lactam and *N*-indole groups in 37 with di-*tert*-butyl dicarbonate (Boc_2_O) under basic conditions produced 39. The reduction of the lactam ring in 39 with Super Hydride® and subsequent trapping of the iminium ion by the C-3 at the indole moiety allowed the construction of the tetracyclic ring 40. The construction of the pentacyclic ring, characteristic of *Aspidosperma* alkaloids, was assembled by the alkylation of 40 with bromoethanol, affording the respective amino alcohol 41. Then, the reaction of the primary alcohol with tosyl chloride (TsCl) in a basic medium, with the concomitant displacement of the corresponding tosyl group by the NH at the indole, facilitated the synthesis of the pentacyclic core. In this way, we synthesized 1,2-dehydroaspidospermidine (42) in 13 steps with a total yield of 3%. With an appropriate modification at the imine segment of 42 in one further step, the synthesis of aspidospermidine (2),^9^ vincadifformine (3)^10^ and winchinine B (43)^11^ might be accomplished.

**Scheme 5:**
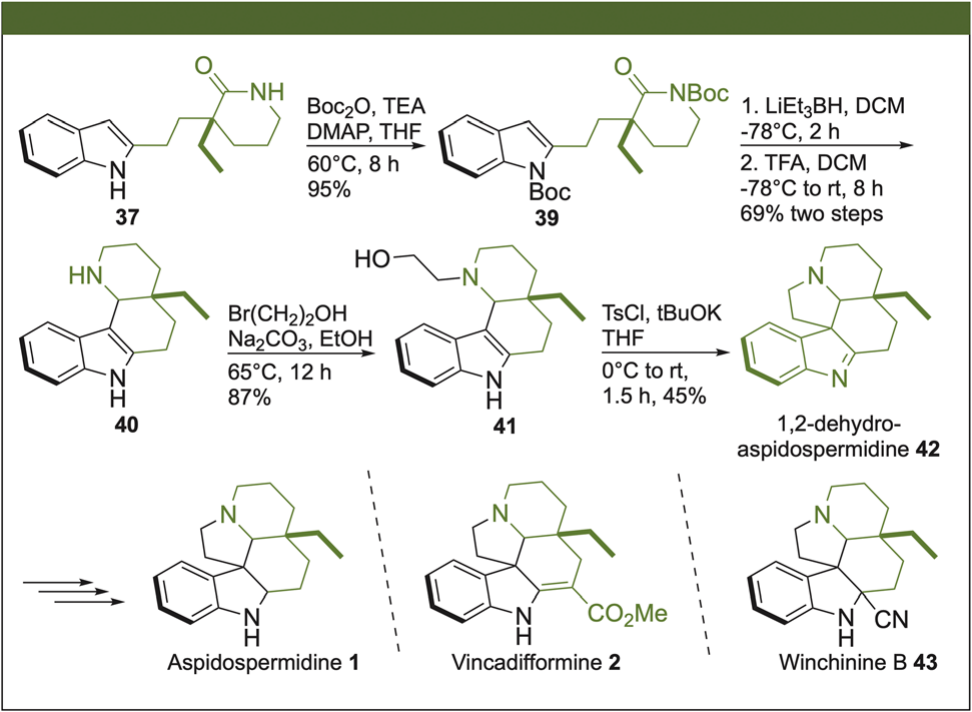
Total synthesis of 1,2-dehydroaspidospermidine (42), and formal synthesis of aspidospermidine (1), vincadifformine (2) and winchinine B (43).

## Conclusions

The C-2 functionalization of various indole derivatives *via* radical-oxidative coupling was achieved. The xanthate precursors utilized as alkylating agents are derived from two distinct lactams, which are of considerable significance due to their presence in numerous monoterpenoid indole alkaloids, such as *Aspidosperma* and ibophyllidine types. The synthesis of 1,2-dehydroaspidospermidine exemplifies the application of this methodology. Furthermore, deoxygenation of the ketone group of three representative intermediates of the indole-2-acetylpiperidine or piperidine type (20–26) was carried out. In principle, modifications of alkyl-type substituents in the keto-amide system, the use of different indole systems with variations at the C-3 position, along with variations of the δ-valerolactam motif with other cyclic amides such as 2-pyrrolidinone, would facilitate the synthesis of analogs and other types of alkaloids.

## Conflicts of interest

There are no conflicts to declare.

## Supplementary Material

RA-015-D5RA01600B-s001

## Data Availability

The data supporting this article have been included as part of the ESI.†

## References

[cit1] Mohammed A. E., Abdul-Hameed Z. H., Alotaibi M. O., Bawakid N. O., Sobahi T. R., Abdel-Lateff A., Alarif W. M. (2021). Molecules.

[cit2] Ma F., Li Y., Akkarasereenon K., Qiu H., Cheung Y. T., Guo Z., Tong R. (2024). Chem. Sci..

[cit3] Mijangos M. V., Miranda L. D. (2018). Org. Biomol. Chem..

[cit4] Takano S., Sato T., Inomata K., Ogasawara K. (1991). J. Chem. Soc., Chem. Commun..

[cit5] Li Y., Vaz R. J., Olson S. H., Munson M., Paras N. A., Conrad J. (2020). Eur. J. Org Chem..

[cit6] Wu X., Huang J., Guo B., Zhao L., Liu Y., Chen J., Cao W. (2014). Adv. Synth. Catal..

[cit7] Randriambola L., Quirion J. C., Kan-Fan C., Husson H. P. (1987). Tetrahedron Lett..

[cit8] Deutsch H. F., Evenson M. A., Drescher P., Sparwasser C., Madsen P. O. (1994). J. Pharm. Biomed. Anal..

[cit9] Jiao L., Herdtweck E., Bach T. (2012). J. Am. Chem. Soc..

[cit10] Jones S. B., Simmons B., Mastracchio A., MacMillan D. W. C. (2011). Nature.

[cit11] Liu Z., Ju X., Ma S., Du C., Zhang W., Li H., Wang X., Xie X., She X. (2019). J. Org. Chem..

