# Correction: C-2 functionalization of indoles with xanthate-lactam derivatives by radical-oxidative coupling, an approach to *Aspidosperma* alkaloids

**DOI:** 10.1039/d6ra90024k

**Published:** 2026-02-25

**Authors:** Manuel Pastrana, Marco V. Mijangos, Luis D. Miranda

**Affiliations:** a Instituto de Química, Universidad Nacional Autónoma de México, Circuito Exterior, Ciudad Universitaria Coyoacán Mexico City 04510 Mexico lmiranda@unam.mx

## Abstract

Correction for ‘C-2 functionalization of indoles with xanthate-lactam derivatives by radical-oxidative coupling, an approach to *Aspidosperma* alkaloids’ by Manuel Pastrana *et al.*, *RSC Adv.*, 2025, **15**, 25694–25697, https://doi.org/10.1039/D5RA01600B.

The authors regret the omission of one of the authors, Marco V. Mijangos, from the original manuscript. The corrected list of authors and affiliations for this article is as shown above.

The Royal Society of Chemistry apologises for these errors and any consequent inconvenience to authors and readers.

