# Supplementary material for: C-2 functionalization of indoles with xanthate-lactam derivatives by radical-oxidative coupling, an approach to Aspidosperma alkaloids
Source: RSC Adv. 2025 Jul 18;15(31):25694–7. doi: 10.1039/d5ra01600b (PMC12273341; doi:10.1039/d5ra01600b)
Supplement: RA-015-D5RA01600B-s001 [file RA-015-D5RA01600B-s001.pdf]

Supplementary Information (SI) for RSC Advances.
This journal is © The Royal Society of Chemistry 2025

SUPPLEMENTARY INFORMATION

C-2 functionalization of indoles with xanthate-lactam derivatives by radical-oxidative coupling, an approach to aspidosperma alkaloids

Manuel Pastrana[a] and Luis D. Miranda[a*]

[a] Instituto de Química, Universidad Nacional Autónoma de México, Circuito Exterior, Ciudad Universitaria, Coyoacán, Mexico City 04510, Mexico.

*Email: lmiranda@unam.mx

Table of Contents

1. GENERAL INFORMATION 2

2. EXPERIMENTAL PROCEDURES 3

3. NMR-SPECTRA 20

4. REFERENCES. 63

1. GENERAL INFORMATION

^{1}H and ^{13}C NMR spectra were obtained on JEOL Eclipse 300 MHz and Bruker Avance III 400 MHz spectrometers. Chemical shifts (δ) are reported in parts per million (ppm) relative to residual proton signal of CHLOROFORM-D and ACETONE-D_6. Coupling constants (*J*) are reported in Hertz. Multiplicity was indicated as follows: s (singlet), d (doublet), t (triplet), q (quartet), p (quintet), m (multiplet), dd (doublet of doublet), bs (broad singlet). HRMS were determined on a JEOL AccuTOF JMS-T100LC with an ionSense DART controller ionization source or an Agilent 6530 Accurate-Mass Q-TOF LC/MS spectrometer, as specified. Infrared spectra were recorded on a Bruker Tensor 27 FT-IR spectrophotometer. Microwave-assisted reactions were performed using a Microwave Synthesis System-CEM reactor. Melting points were determined on a Fisher apparatus and are uncorrected. Thin layer chromatograms were performed on precoated TLC sheets of silica gel 60 F254 (E. Merck). Flash chromatography was carried out using silica gel (Merck 230–400 mesh). All reactions were carried out under an argon atmosphere in oven- or flamedried glassware unless the reaction procedure states otherwise. Degassed solutions were obtained by freeze-pump-thaw cycles (3x) using liquid nitrogen. All reagents and solvents were purchased from Sigma-Aldrich and Tecsiquim and were used without further purification.

2. EXPERIMENTAL PROCEDURES

General procedure A. lactam *N*-benzylation

*Compound **9**. 1-benzylpyrrolidin-2-one.*

To a solution of 2-pyrrolidone (**7**) (5.0 g, 58.8 mmol, 1.0 equiv) in PhMe (60 mL, 1.0 M) was added KOH (13.18 g, 235.0 mmol, 4.0 equiv), BnBr (14 mL, 117.5 mmol, 2.0 equiv) and TBAB (1.89 g, 5.87 mmol, 0.1 equiv). The resulting suspension was stirred at room temperature for 48 hours. After the reaction was completed, TLC analysis (EtOAc 100%), the solvent was removed under reduced pressure and the crude was diluted with EtOAc (30 mL) and extracted with brine (30 mL), the aqueous phase was extracted with EtOAc (20 mL). The organic phases were put together and dried over anhydrous Na_2SO_4, filtered and evaporated under reduced pressure. The residue was purified by flash column chromatography using silica gel (EtOAc 100% as eluent). Compound **9** was obtained as a pale-yellow oil (8.82 g, 50.35 mmol, 85% yield). **^{1}H NMR** (400 MHz, CHLOROFORM-*D*) δ 7.39 – 7.26 (m, 5H), 4.49 (s, 2H), 3.30 (t, *J* = 8.0 Hz, 2H), 2.48 (t, *J* = 8.1 Hz, 2H), 2.03 (q, *J* = 7.2, 7.7 Hz, 2H). **^{13}C NMR** (100 MHz, CHLOROFORM-*D*) δ 175.0, 136.6, 128.7, 128.1, 127.6, 46.6, 31.0, 17.7. **FT-IR** (ATR) cm^{-1}: 3469, 3029, 2974, 2945, 2914, 2878, 1673, 1494, 1421, 1285, 1261, 1163, 1082, 923, 699. **HRMS** (DART) *m/z* calcd for $C_{11}H_{14}NO$ [M+H$^+$]: 176.1075; found: 176.1069.

*Compound **10**. 1-benzylpiperidin-2-one.*

Following the general procedure A. δ-valerolactam (**8**) (5.0 g, 50.43 mmol, 1.0 equiv), PhMe (50 mL, 1.0 M), KOH (11.32 g, 201.74 mmol, 4.0 equiv), BnBr (12 mL, 100.87 mmol, 2.0 equiv) and TBAB (1.63 g, 5.04 mmol, 0.1 equiv). Compound **10** was obtained as a pale-yellow oil (8.4 g, 44.37 mmol, 88% yield). **^{1}H NMR** (400 MHz, CHLOROFORM-*D*) δ 7.32 – 7.22 (m, 5H), 4.58 (s, 2H), 3.17 (t, *J* = 6.2 Hz, 2H), 2.45 (t, *J* = 6.3 Hz, 2H), 1.82 – 1.71 (m, 4H). **^{13}C NMR** (100 MHz, CHLOROFORM-*D*) δ 169.8, 137.3, 128.6, 128.0, 127.3, 50.0, 47.2, 32.4, 23.1, 21.4. **FT-IR** (ATR) cm^{-1}: 3455, 3261, 3059, 3028, 2940, 2865, 1629, 1492, 1446, 1417, 1350, 1255, 1171, 1070, 991, 952, 702. **HRMS** (DART) *m/z* calcd for $C_{12}H_{16}NO$ [M+H$^+$]: 190.1231; found: 190.1233.

General procedure B. α-carbonyl acylation

*Compound **11**. 3-acetyl-1-benzylpyrrolidin-2-one.*

A solution of diisopropylamine (5.7 mL, 40.49 mmol, 1.1 equiv) in freshly distilled THF (73 mL, 0.5 M) was cooled at –78 °C in an acetone/dry ice bath. Then *n*-BuLi 2.5M (16.2 mL, 40.49 mmol, 1.1 equiv) was slowly added via syringe and the solution was stirred 30 minutes at –78 °C. A solution of **9** (6.45 g, 36.81 mmol, 1.0 equiv) in THF (6.5 mL) was slowly transferred via syringe and left to react for 1 hour at –78 °C. Then MeOAc (4.4 mL, 55.22 mmol, 1.5 equiv) was slowly added, and the reaction was allowed to reach room temperature over 11 hours under nitrogen atmosphere. After the reaction was completed, TLC analysis (hexane/EtOAc 1:1), the crude was diluted with EtOAc (30 mL) and extracted with brine (50 mL). The aqueous phase was extracted with EtOAc (20 mL). The organic phases were put together and dried over anhydrous Na_2SO_4, filtered

and evaporated under reduced pressure. The residue was purified by flash column chromatography using silica gel (hexane/EtOAc 1:1 as eluent). Compound **11** was obtained as a pale-yellow oil (6.48 g, 29.81 mmol, 81% yield). **^{1}H NMR** (400 MHz, CHLOROFORM-*D*) δ 7.41 – 7.29 (m, 3H), 7.27 – 7.25 (m, 2H), 4.49 (q, *J* = 14.7 Hz, 2H), 3.70 (dd, *J* = 9.3, 6.1 Hz, 1H), 3.36 (td, *J* = 9.3, 5.4 Hz, 1H), 3.25 (td, *J* = 9.2, 5.5 Hz, 1H), 2.61 – 2.53 (m, 1H), 2.51 (s, 3H), 2.11 – 2.02 (m, 1H). **^{13}C NMR** (100 MHz, CHLOROFORM-*D*) δ 203.8, 169.9, 136.0, 128.9, 128.1, 127.8, 55.8, 47.0, 45.0, 30.1, 19.6. **FT-IR** (ATR) cm^{-1}: 3498, 3062, 3029, 2988, 2890, 1713, 1674, 1494, 1452, 1426, 1356, 1258, 1163, 1080, 737, 699. **HRMS** (DART) *m/z* calcd for $C_{13}H_{16}NO_2$ [M+H$^+$]: 218.1181; found: 218.1170.

__Compound 12. 3-acetyl-1-benzylpiperidin-2-one.__

Following the general procedure B. Diisopropylamine (6.0 mL, 42.30 mmol, 1.1 equiv), THF (70 mL, 0.5 M), *n*-BuLi 2.5M (16.9 mL, 42.30 mmol, 1.1 equiv), **10** (7.28 g, 38.46 mmol, 1.0 equiv) in THF (7.3 mL) and MeOAc (4.6 mL, 57.69 mmol, 1.5 equiv). Compound **12** was obtained as a pale-yellow oil (7.93 g, 34.26 mmol, 89% yield) as a mixture of keto and enol tautomers 5:4. **^{1}H NMR** (400 MHz, CHLOROFORM-*D*), keto form δ 7.36 – 7.23 (m, 5H), 4.66 (d, *J* = 15.2 Hz, 1H), 4.53 (d, *J* = 15.1 Hz, 1H), 3.57 (t, *J* = 6.4 Hz, 1H), 3.27 – 3.16 (m, 2H), 2.37 (s, 3H), 2.18 – 2.08 (m, 1H), 1.91 – 1.83 (m, 2H), 1.74 – 1.66 (m, 1H); enol form δ 14.95 (s, 1H), 7.36 – 7.23 (m, 5H), 4.61 (s, 2H), 3.27 – 3.16 (m, 2H), 2.41 – 2.37 (m, 2H), 1.96 (s, 3H), 1.83 – 1.74 (m, 2H). **^{13}C NMR** (100 MHz, CHLOROFORM-*D*) δ 205.9, 170.5, 169.2, 166.6, 137.3, 136.9, 128.7, 128.7, 128.0, 127.8, 127.5, 127.4, 95.7, 55.6, 50.4, 49.9, 47.3, 47.2, 30.2, 24.2, 23.7, 22.6, 21.0, 18.7. **FT-IR** (ATR) cm^{-1}:3060, 3028, 2930, 2857, 1715, 1629, 1592, 1491, 1442, 1255, 1200, 954, 696. **HRMS** (DART) *m/z* calcd for $C_{14}H_{18}NO_2$ [M+H$^+$]: 232.1337; found: 232.1328.

__General procedure C. 1,3-dicarbonyl alkylation__

__Compound 13. 3-acetyl-1-benzyl-3-ethylpyrrolidin-2-one.__

To a solution of **11** (3.0 g, 13.80 mmol, 1.0 equiv) in DMF (27 mL, 0.5 M) was added K_2CO_3 (5.72 g, 41.42 mmol, 3.0 equiv) and the suspension was stirred 30 minutes at room temperature. Then ethyl iodide (1.7 mL, 20.71 mmol, 1.5 equiv) was added, and the reaction was stirred 24 hours at room temperature. After the reaction was completed, TLC analysis (hexane/EtOAc 3:1), the crude was diluted with EtOAc (30 mL) and extracted with brine (50 mL). The aqueous phase was extracted with EtOAc (20 mL). The organic phases were put together and dried over anhydrous Na_2SO_4, filtered and evaporated under reduced pressure. The residue was purified by flash column chromatography using silica gel (hexane/EtOAc 3:1 as eluent). Compound **13** was obtained as a pale-yellow oil (2.56 g, 10.45 mmol, 76% yield). **^{1}H NMR** (400 MHz, CHLOROFORM-*D*) δ 7.35 – 7.26 (m, 3H), 7.21 – 7.18 (m, 2H), 4.51 (d, *J* = 14.7 Hz, 1H), 4.39 (d, *J* = 14.7 Hz, 1H), 3.24 – 3.18 (m, 1H), 3.12 (td, *J* = 9.4, 3.4 Hz, 1H), 2.68 (ddd, *J* = 13.0, 8.0, 3.4 Hz, 1H), 2.35 (s, 3H), 2.13 (dt, *J* = 14.8, 7.5 Hz, 1H), 1.86 (dq, *J* = 14.8, 7.5 Hz, 1H), 1.74 (ddd, *J* = 13.0, 9.1, 7.5 Hz, 1H), 0.87 (t, *J* = 7.5 Hz, 3H). **^{13}C NMR** (100 MHz, CHLOROFORM-*D*) δ 206.1, 172.4, 136.2, 128.8, 128.0, 127.7, 63.6, 47.1, 43.9, 28.0, 26.2, 25.0, 9.1. **FT-IR** (ATR) cm^{-1}:2966, 2934,

2879, 1677, 1494, 1424, 1355, 1263, 1203, 734, 700. **HRMS** (DART) *m/z* calcd for $C_{15}H_{20}NO_2$ [M+H$^+$]: 246.1494; found: 246.1486.

*Compound **14**. 3-acetyl-1-benzyl-3-ethylpiperidin-2-one.*

Following the general procedure C. **12** (3.0 g, 12.97 mmol, 1.0 equiv), DMF (26 mL, 0.5 M), K_2CO_3 (5.38 g, 39.91 mmol, 3.0 equiv) and ethyl iodide (1.6 mL, 19.45 mmol, 1.5 equiv). Compound **14** was obtained as a pale-yellow oil (2.76 g, 10.65 mmol, 82% yield). **^{1}H NMR** (400 MHz, CHLOROFORM-*D*) δ 7.35 – 7.22 (m, 5H), 4.70 (d, *J* = 15 Hz, 1H), 4.51 (d, *J* = 14.5 Hz, 1H), 3.23 – 3.15 (m, 2H), 2.31 – 2.25 (m, 1H), 2.26 (s, 3H), 2.07 – 1.98 (m, 1H), 1.96 – 1.87 (m, 1H), 1.77 – 1.65 (m, 2H), 1.57 (ddd, *J* = 13.4, 9.8, 5.3 Hz, 1H), 0.87 (t, *J* = 7.4 Hz, 3H). **^{13}C NMR** (100 MHz, CHLOROFORM-*D*) δ 207.8, 170.1, 137.3, 128.7, 128.1, 127.5, 60.8, 50.9, 47.6, 29.6, 27.4, 27.0 20.2, 9.1. **FT-IR** (ATR) cm^{-1}: 2963, 2938, 2877, 1704, 1628, 1489, 1451, 1352, 1259, 1197, 1156, 699. **HRMS** (DART) m/*z* calcd for $C_{16}H_{22}NO_2$ [M+H$^+$]: 260.1651; found: 260.1646.

General procedure D. α-bromination.

*Compound **15**. 1-benzyl-3-(2-bromoacetyl)-3-ethylpyrrolidin-2-one.*

To a solution of **13** (3.25 g, 13.23 mmol, 1.0 equiv) in freshly distilled THF (44 mL, 0.3 M) was cooled to –78 °C in acetone/dry ice bath. NaHMDS 1.0 M (14.6 mL, 14.6 mmol, 1.1 equiv) was slowly added via syringe and the solution was stirred 1 hour at –78 °C. TMSCl (2.5 mL, 19.85 mmol, 1.5 equiv) was slowly added, and the solution was stirred 1 hour at –78 °C. Br_2 (0.74 mL, 14.56 mmol, 1.1 equiv) was slowly added at –78 °C, and the reaction was allowed to reach room temperature over 10 hours under nitrogen atmosphere. After the reaction was completed, TLC analysis (hexane/EtOAc 4:1), the crude was diluted with EtOAc (30 mL) and extracted with brine (40 mL). The aqueous phase was extracted with EtOAc (20 mL). The organic phases were put together and dried over anhydrous Na_2SO_4, filtered and evaporated under reduced pressure. The residue was purified by flash column chromatography using silica gel (hexane/EtOAc 5:1 as eluent). Compound **15** was obtained as a pale-yellow oil (3.60 mg, 11.11 mmol, 84% yield). **^{1}H NMR** (400 MHz, CHLOROFORM-*D*) δ 7.33 – 7.24 (m, 3H), 7.18 – 7.14 (m, 2H), 4.59 (d, *J* = 14.7 Hz, 1H), 4.51 (d, *J* = 14.7 Hz, 1H), 4.34 (t, *J* = 14.8 Hz, 2H), 3.23 – 3.09 (m, 2H), 2.72 (ddd, *J* = 13.3, 8.2, 4.0 Hz, 1H), 2.13 – 2.04 (m, 1H), 1.91 – 1.76 (m, 2H), 0.86 (t, *J* = 7.5 Hz, 3H). **^{13}C NMR** (100 MHz, CHLOROFORM-*D*) δ 198.9, 171.6, 135.8, 128.9, 128.1, 127.9, 63.2, 47.3, 43.8, 33.0, 28.6, 25.2, 9.1. **FT-IR** (ATR) cm^{-1}: 3029, 2966, 2936, 2878, 1722, 1675, 1494, 1428, 1261, 1042, 1028, 737, 699. **HRMS** (DART) *m/z* calcd for $C_{15}H_{19}BrNO_2$ [M+H$^+$]: 324.0599; found: 324.0610.

*Compound **16**. 1-benzyl-3-(2-bromoacetyl)-3-ethylpiperidin-2-one.*

Following the general procedure D. **14** (3.15 g, 12.15 mmol, 1.0 equiv), THF (40 mL, 0.3 M), NaHMDS 1.0 M (13.6 mL, 13.6 mmol, 1.1 equiv), TMSCl (2.3 mL, 18.22 mmol, 1.5 equiv) and Br_2 (0.68 mL, 13.65 mmol, 1.1 equiv). Compound **16** was obtained as a pale-yellow oil (3.67 mg, 10.85 mmol, 89% yield). **^{1}H NMR** (400 MHz, CHLOROFORM-*D*) δ 7.27 – 7.12 (m, 5H), 4.51 (q, *J* = 14.5 Hz, 2H), 4.33 (q, *J* = 14.1 Hz, 2H), 3.15 – 3.11 (m, 2H),

2.36 – 2.30 (m, 1H), 1.99 – 1.83 (m, 2H), 1.72 – 1.56 (m, 2H), 1.50 (ddd, *J* = 13.4, 11.2, 3.7 Hz, 1H), 0.78 (t, *J* = 7.4 Hz, 3H). **^{13}C NMR** (100 MHz, CHLOROFORM-*D*) δ 200.4, 169.3, 136.9, 128.8, 128.2, 127.7, 60.6, 51.2, 47.8, 33.6, 30.6, 27.7, 20.1, 9.2. **FT-IR** (ATR) cm^{-1}: 3028, 2938, 2877, 1753, 1719, 1624, 1490, 1451, 1353, 1259, 1199, 1165, 1029, 734, 698. **HRMS** (DART) *m/z* calcd for $C_{16}H_{21}BrNO_2$ [M+H$^+$]: 340.0735; found: 340.0745.

General procedure E. α-ketylmethyl xanthate

*Compound **17**. S-(2-(1-benzyl-3-ethyl-2-oxopyrrolidin-3-yl)-2-oxoethyl)-O-ethyl carbonodithioate.*

To a solution of **15** (3.56 g, 10.98 mmol, 1.0 equiv) in a mixture of MeCN-H_2O 1:1 (33 mL, 0.3 M) was added KSCSOEt (2.29 g, 14.27 mmol, 1.3 equiv), and the resulting solution was stirred at room temperature for 12 hours. After the reaction was completed, TLC analysis (hexane/EtOAc 4:1), the volatiles were removed under reduced pressure and the crude was diluted with EtOAc (30 mL) and extracted with brine (20 mL); the aqueous phase was extracted with EtOAc (20 mL). The organic phases were put together and dried over anhydrous Na_2SO_4, filtered and evaporated under reduced pressure. The residue was purified by flash column chromatography using silica gel (hexane/EtOAc 5:1). Compound **17** was obtained as a pale-yellow oil (3.61 g, 9.88 mmol, 90% yield). **^{1}H NMR** (400 MHz, CHLOROFORM-*D*) δ 7.42 – 7.33 (m, 3H), 7.29 – 7.26 (m, 2H), 4.78 – 4.64 (m, 4H), 4.47 – 4.39 (m, 2H), 3.33 – 3.19 (m, 2H), 2.81 (ddd, *J* = 13.3, 8.1, 3.7 Hz, 1H), 2.26 (sext, *J* = 7.5 Hz, 1H), 2.01 (sext, *J* = 7.4 Hz, 1H), 1.88 (ddd, *J* = 13.3, 9.0, 7.1 Hz, 1H), 1.47 (t, *J* = 7.1 Hz, 3H), 0.99 (t, *J* = 7.5 Hz, 3H). **^{13}C NMR** (100 MHz, CHLOROFORM-*D*) δ 213.7, 199.9, 172.0, 136.0, 128.9, 128.1, 127.8, 70.7, 63.5, 47.3, 44.0, 43.0, 28.4, 25.3, 13.8, 9.1. **FT-IR** (ATR) cm^{-1}: 2966, 2934, 2878, 1717, 1678, 1452, 1427, 1359, 1262, 1217, 1111, 1039, 736, 699. **HRMS** (DART) *m/z* calcd for $C_{18}H_{24}NO_3S_2$ [M+H$^+$]: 366.1197; found: 366.1215.

*Compound **18**. S-(2-(1-benzyl-3-ethyl-2-oxopiperidin-3-yl)-2-oxoethyl)-O-ethyl carbonodithioate.*

Following the general procedure E. **16** (3.67 g, 10.85 mmol, 1.0 equiv), MeCN-H_2O 1:1 (30 mL, 0.3 M) and KSCSOEt (2.26 g, 14.10 mmol, 1.3 equiv). Compound **18** was obtained as a pale-yellow oil (3.80 g, 10.03 mmol, 92% yield). **^{1}H NMR** (400 MHz, CHLOROFORM-*D*) δ 7.36 – 7.25 (m, 5H), 4.71 – 4.59 (m, 4H), 4.49 (d, *J* = 0.9 Hz, 2H), 3.25 (t, 2H, *J* = 8 Hz), 2.47 – 2.42 (m, 1H), 2.18 – 2.08 (m, 1H), 2.05 – 1.96 (m, 1H), 1.80 – 1.73 (m, 2H), 1.62 (ddd, *J* = 13.4, 8.8, 6.5 Hz, 1H), 1.41 (t, *J* = 7.1 Hz, 3H), 0.91 (t, *J* = 7.4 Hz, 3H). **^{13}C NMR** (100 MHz, CHLOROFORM-*D*) δ 213.9, 201.6, 169.6, 137.0, 128.8, 128.2, 127.7,70.7, 60.8, 51.1, 47.8, 44.0, 30.4, 27.8, 20.2, 13.9, 9.2. **FT-IR** (ATR) cm^{-1}: 2963, 2937, 2877, 1715, 1626, 1489, 1450, 1353, 1216, 1111, 1049, 1036, 733, 699. **HRMS** (DART) *m/z* calcd for $C_{19}H_{26}NO_3S_2$ [M+H$^+$]: 380.1354; found: 380.1337.

General procedure F, indol *N*-benzylation.

*Compound **19a**. 1-benzyl-1H-indole.*

To a solution of indole (2.0 g, 17.07 mmol, 1.0 equiv) in THF (35 mL, 0.5 M) at 0 °C was added NaH (887 mg, 22.19 mmol, 1.3 equiv) and the resulting suspension was stirred 30 minutes at 0 °C. Benzyl bromide (2.6 mL, 22.19 mmol, 1.3 equiv) was added, and the reaction was stirred for 6 hours at room temperature. After the

reaction was completed, TLC analysis (hexane/EtOAc 20:1), the crude was diluted with EtOAc (20 mL) and extracted with brine (30 mL). The aqueous phase was extracted with EtOAc (20 mL). The organic phases were put together and dried over anhydrous Na_2SO_4, filtered and evaporated under reduced pressure. The residue was purified by flash column chromatography using silica gel (hexane/EtOAc). The product **19a** was obtained as a pale-yellow solid (3.18 g, 15.35 mmol, 90% yield), mp 42-44 °C. **^{1}H NMR** (400 MHz, CHLOROFORM-*D*) δ 7.68 (d, *J* = 7.6 Hz, 1H), 7.34 – 7.25 (m, 4H), 7.19 (t, *J* = 6.9 Hz, 1H), 7.16 – 7.11 (m, 4H), 6.58 (d, *J* = 3.2 Hz, 1H), 5.34 (s, 2H). **^{13}C NMR** (100 MHz, CHLOROFORM-*D*) δ 137.7, 136.5, 128.9, 128.4, 127.7, 126.9, 121.8, 121.1, 119.7, 109.8, 101.8, 50.2. **FT-IR** (ATR) cm^{-1}: 3119, 3092, 3050, 3025, 2929, 2865, 1586, 1507, 1486, 1456, 1395, 1315, 1254, 1206, 1176, 1076, 1005, 915, 735, 712. **HRMS** (DART) *m/z* calcd for $C_{15}H_{14}N$ [M+H$^+$]:208.1126; found: 208.1122.

*Compound **19b**. 1-benzyl-3-(2-((tert-butyldimethylsilyl)oxy)ethyl)-1H-indole.*

To a solution of tryptophol (1.6 g, 9.92 mmol, 1.0 equiv) in THF (33 mL, 0.3 M) were added TBDMSCl (1.8 g, 11.91 mmol, 1.2 equiv) and imidazole (878 mg, 12.90 mmol, 1.3 equiv) and the suspension was stirred 5 hours at room temperature. After the reaction was completed, TLC analysis (hexane/EtOAc 5:1), the crude was diluted with EtOAc (20 mL) and extracted with brine (30 mL). The aqueous phase was extracted with EtOAc (20 mL). The organic phases were put together and dried over anhydrous Na_2SO_4, filtered and evaporated under reduced pressure. The residue was purified by flash column chromatography using silica gel (hexane/EtOAc). tryptophol-TBS was obtained as a pale-red oil (2.57 g, 9.32 mmol, 94% yield). **^{1}H NMR** (400 MHz, CHLOROFORM-*D*) δ 7.96 (s, 1H), 7.67 (d, *J* = 7.9 Hz, 1H), 7.37 (d, *J* = 8.0 Hz, 1H), 7.24 (t, *J* = 7.6 Hz, 1H), 7.18 (t, *J* = 7.5 Hz, 1H), 7.04 (d, *J* = 2.3 Hz, 1H), 3.95 (t, *J* = 7.4 Hz, 2H), 3.06 (t, *J* = 7.3 Hz, 2H), 0.98 (s, 10H), 0.10 (s, 6H). **^{13}C NMR** (100 MHz, CHLOROFORM-*D*) δ 136.3, 127.8, 122.2, 122.0, 119.3, 119.0, 113.2, 111.2, 64.0, 29.2, 26.1, 18.5, -5.1. The spectroscopic data correspond with the literature.[1]

Following the general procedure F. Tryptophol-TBS (2.0 g, 7.26 mmol, 1.0 equiv), THF (14 mL, 0.5 M), NaH (377 mg, 9.44 mmol, 1.3 equiv) and benzyl bromide (1.1 mL, 9.44 mmol, 1.3 equiv). The residue was purified by flash column chromatography (hexane/EtAOc 5:1 as eluent). Product **19b** was obtained as a pale-yellow oil (2.15 g, 5.88 mmol, 81% yield). **^{1}H NMR** (400 MHz, CHLOROFORM-*D*) δ 7.63 (ddd, *J* = 7.7, 1.4, 0.8 Hz, 1H), 7.32 – 7.22 (m, 4H), 7.20 – 7.09 (m, 4H), 6.97 (s, 1H), 5.27 (s, 2H), 3.90 (t, *J* = 7.3 Hz, 2H), 3.00 (t, *J* = 7.8 Hz, 2H), 0.90 (s, 9H), 0.03 (s, 6H). **^{13}C NMR** (100 MHz, CHLOROFORM-*D*) δ 137.9, 136.7, 128.9, 128.5, 127.7, 127.0, 126.4, 121.8, 119.2, 119.1, 112.5, 109.7, 64.1, 50.0, 29.1, 26.1, 18.5, -5.1. **FT-IR** (ATR) cm^{-1}: 3057, 3029, 2951, 2927, 2896, 2854, 2161, 1610, 1494, 1465, 1356, 1329, 1251, 1173, 1088, 917, 832, 733. **HRMS** (DART) *m/z* calcd for $C_{23}H_{32}NOSi$ [M+H$^+$]:366.2253; found: 366.2245.

*Compound **19c**. tert-butyl (2-(1-benzyl-1H-indol-3-yl)ethyl)carbamate.*

To a solution of tryptamine (1.5 g, 9.36 mmol, 1.0 equiv) in THF (31 mL, 0.3 M) were added TEA (1.7 mL, 12.17 mmol, 1.3 equiv) and Boc_2O (2.45 g, 11.23 mmol, 1.2 equiv) and the reaction was stirred for 5 hours at room

temperature. After the reaction was completed, TLC analysis (hexane/EtOAc 2:1), the crude was diluted with EtOAc (20 mL) and extracted with brine (30 mL). The aqueous phase was extracted with EtOAc (20 mL). The organic phases were put together and dried over anhydrous Na_2SO_4, filtered and evaporated under reduced pressure. The residue was purified by flash column chromatography using silica gel (hexane/EtOAc 2:1). tryptamine-Boc was obtained as a withe solid (2.36 g, 9.07 mmol, 97% yield), mp 89-91 °C. **^{1}H NMR** (400 MHz, CHLOROFORM-*D*) δ 8.09 (s, 1H), 7.61 (d, *J* = 7.9 Hz, 1H), 7.37 (dt, *J* = 8.1, 1.0 Hz, 1H), 7.21 (ddd, *J* = 8.1, 7.0, 1.2 Hz, 1H), 7.13 (ddd, *J* = 8.0, 7.0, 1.0 Hz, 1H), 7.03 (s, 1H), 4.50 (s, 1H), 3.47 (s, 2H), 2.97 (d, *J* = 7.3 Hz, 2H), 1.44 (s, 9H). **^{13}C NMR** (100 MHz, CHLOROFORM-*D*) δ 156.1, 136.5, 127.5, 122.3, 122.2, 119.6, 119.0, 113.2, 111.3, 79.1, 40.5, 27.9, 26.0. The spectroscopic data correspond with the literature.[2]

Following the general procedure F. Tryptamine-Boc (2.0 mg, 7.68 mmol, 1.0 equiv), THF (15 mL, 0.5 M), NaH (400 mg, 9.98 mmol, 1.3 equiv) and benzyl bromide (1.2 mL, 9.98 mmol, 1.3 equiv) The residue was purified by flash column chromatography (hexane/EtAOc 4:1 as eluent). Product **19c** was obtained as a pale-yellow oil (2.26 g, 6.45 mmol, 84% average yield). **^{1}H NMR** (400 MHz, CHLOROFORM-*D*) δ 7.62 (d, *J* = 7.8, 1H), 7.32 – 7.24 (m, 4H), 7.19 (ddd, *J* = 8.2, 6.9, 1.2 Hz, 1H), 7.14 – 7.10 (m, 3H), 6.96 (s, 1H), 5.28 (s, 2H), 4.60 (s, 1H), 3.46 (d, *J* = 6.8 Hz, 2H), 2.96 (t, *J* = 6.9 Hz, 2H), 1.44 (s, 9H). **^{13}C NMR** (100 MHz, CHLOROFORM-*D*) δ 155.6, 137.7, 136.9, 128.9, 128.2, 127.7, 126.3, 121.4, 119.3, 119.2, 112.5, 109.9, 79.2, 50.1, 41.1, 28.6, 25.9. **FT-IR** (ATR) cm^{-1}: 3358, 3257, 3120, 3084, 2979, 2964, 2923, 2865, 2013, 1691, 1469, 1440, 1390, 1360, 1262, 1159, 1116, 1006, 732. **HRMS** (DART) *m/z* calcd for $C_{22}H_{27}N_2O_2$ [M+H$^+$]:351.2072; found: 351.2068.

*Compound **19d**. methyl 1-benzyl-1H-indole-3-carboxylate.*

Following the general procedure F. Methyl indole-3-carboxylate (1.5 g, 7.93 mmol, 1.0 equiv), THF (16 mL, 0.5 M), NaH (412 mg, 10.3 mmol, 1.3 equiv) and benzyl bromide (1.2 mL, 10.3 mmol, 1.3 equiv). The residue was purified by flash column chromatography (hexane/EtAOc 3:1 as eluent). Product **19d** was obtained as yellow solid (1.92 g, 6.87 mmol, 86% yield), mp 101-103 °C. **^{1}H NMR** (400 MHz, CHLOROFORM-*D*) δ 8.21 (ddd, *J* = 7.8, 1.6, 0.9 Hz, 1H), 7.83 (s, 1H), 7.34 – 7.21 (m, 6H), 7.15 – 7.13 (m, 2H), 5.30 (s, 2H), 3.90 (s, 3H). **^{13}C NMR** (100 MHz, CHLOROFORM-*D*) δ 165.5, 136.9, 136.0, 134.7, 129.1, 128.2, 127.2, 126.9, 123.1, 122.1, 121.9, 110.4, 107.7, 51.1, 50.8. **FT-IR** (ATR) cm^{-1}: 3107, 3058, 3032, 2997, 2925, 2182, 2024, 1687, 1527, 1491, 1458, 1435, 1384, 1237, 1175, 1090, 1030, 926, 730. **HRMS** (DART) *m/z* calcd for $C_{17}H_{16}NO_2$ [M+H$^+$]:266.1181; found: 266.1172.

*Compound **19e**. ethyl 2-(1-benzyl-1H-indol-3-yl)acetate.*

To a solution of indol-3-acetic acid (1.50 g, 8.56 mmol, 1.0 equiv) in EtOH (34 mL, 0.2 M) at room temperature was added $SOCl_2$ (2.0 mL, 25.68 mmol, 3.0 equiv) dropwise and then allowed to reflux 12 hours. After the reaction was completed, TLC analysis (hexane/EtOAc 2:1), the volatiles were removed under reduced pressure and the crude was diluted with EtOAc (30 mL) and extracted with $NaHCO_3$ (20 mL); the aqueous phase was extracted with EtOAc (20 mL). The organic phases were put together and dried over anhydrous Na_2SO_4, filtered

and evaporated under reduced pressure. The residue was purified by flash column chromatography using silica gel (hexane/EtOAc 3:1). The product **19e** was obtained as a pale-red oil (1.53 g, 7.53 mmol, 88 % yield). **^{1}H NMR** (400 MHz, CHLOROFORM-*D*) δ 8.10 (s, 1H), 7.64 (d, *J* = 7.7 Hz, 1H), 7.35 (d, *J* = 8.2 Hz, 1H), 7.25 – 7.10 (m, 3H), 4.18 (q, *J* = 7.1 Hz, 2H), 3.78 (s, 2H), 1.27 (t, *J* = 7.1 Hz, 3H). **^{13}C NMR** (100 MHz, CHLOROFORM-*D*) δ 172.2, 136.2, 127.4, 123.2, 122.3, 119.8, 119.0, 111.3, 60.9, 31.6, 14.4. **FT-IR** (ATR) cm^{-1}: 3404, 3055, 2980, 2904, 1717, 1456, 1424, 1368, 1335, 1298, 1157, 1093, 1025, 739. The spectroscopic data correspond with the literature.[3]

General procedure G. Radical-oxidative coupling.

*Compound **20**. 1-benzyl-3-(2-(1-benzyl-1H-indol-2-yl)acetyl)-3-ethylpyrrolidin-2-one.*

To a solution of xanthate **17** (304 mg, 0.83 mmol, 1.0 equiv) in DCE (3.3 mL, 0.25 M) was added **19a** (224 mg, 1.08 mmol, 1.3 equiv) and the resulting mixture was degassed by three consecutive freeze-pump-thaw cycles using liquid nitrogen and back filled with pure argon. The reaction mixture was refluxed and DLP (596 mg, 1.49 mmol, 1.8 equiv) was added under nitrogen atmosphere in ratio of 0.3 equiv/h (six additions for 7 hours of reaction). After the reaction was completed, TLC analysis (hexane/EtOAc 5:1), the crude was cooled to room temperature and the solvent was removed under reduced pressure. The residue was purified by flash column chromatography using silica gel. Compound **20** was obtained as a pale-yellow oil (140 mg, 0.31 mmol, 37 % yield). **^{1}H NMR** (400 MHz, ACETONE-D_6) δ 7.54 - 7.53 (m, 1H), 7.52 - 7.50 (m, 1H), 7.30 - 7.22 (m, 7H), 7.19 – 7.18 (m, 2H), 7.07 – 6.99 (m, 4H), 5.36 (dd, *J*= 4Hz, 2H), 4.53 (d, *J*=20Hz, 1H), 4.43 (d, *J*=16Hz, 1H), 4.32 (d, *J*=16Hz, 1H), 4.03 (d, *J*=16Hz, 1H), 3.15 (m, 2H), 2.64-2.58 (m, 1H), 2.09 (dq, *J* = 14.8, 7.4 Hz, 1H), 1.89 (dq, *J* = 14.7, 7.5 Hz, 1H), 0.87 (t, *J*=7.5 Hz, 3H).**^{13}C NMR** (100 MHz, ACETONE-D_6) δ 204.3, 172.8, 139.4, 138.4, 137.7, 134.8, 129.5, 129.4, 129.1, 128.8, 128.3, 128.0, 127.1, 121.8, 120.8, 120.2, 110.7, 103.4, 64.0, 47.3, 44.5, 36.9, 28.2, 25.7, 9.3. **FT-IR** (ATR) cm^{-1}: 3060, 3029, 2965, 2927, 2878, 1710, 1677, 1494, 1452, 1425, 1353, 1313, 1262, 1196, 1175, 1028, 912, 727, 697. **HRMS** (DART) *m/z* calcd for $C_{30}H_{31}N_2O_2$ [M+H$^+$]:451.2385; found: 451.2364.

*Compound **21**. 1-benzyl-3-(2-(1-benzyl-3-(2-((tert-butyldimethylsilyl)oxy)ethyl)-1H-indol-2-yl)acetyl)-3-ethylpyrrolidin-2-one.*

Following the general procedure G. **17** (312 mg, 0.85 mmol, 1.0 equiv), **19b** (405 mg, 1.11 mmol, 1.3 equiv), DLP (612 mg, 1.53 mmol, 1.8 equiv) and DCE (3.4 mL, 0.25 M). The residue was purified by flash column chromatography (hexane/EtAOc 8:1 as eluent). Compound **21** was obtained as a pale-yellow oil (169 mg, 0.28 mmol, 33% yield). **^{1}H NMR** (400 MHz, CHLOROFORM-*D*) δ 7.80 – 7.77 (m, 1H), 7.53 – 7.26 (m, 11H), 7.14 (d, *J* = 8.0 Hz, 2H), 5.38 (q, *J* = 16, 8.0 Hz, 2H), 4.75 (t, *J* = 18 Hz, 2H), 4.20 (t, *J* = 18 Hz, 2H), 3.99 (t, *J* = 8.0 Hz, 2H), 3.26 (m, *J* = 6 Hz, 2H), 3.12 (t, *J* = 6 Hz, 2H), 2.82 (dt, *J* = 11.9, 5.7 Hz, 1H), 2.38 (dq, *J* = 14.6, 7.4 Hz, 1H), 2.13 (dq, *J* = 14.6, 7.4 Hz, 1H), 1.92 (dt, *J* = 15.9, 9.6 Hz, 1H), 1.09 (m, 12H), 0.21 (d, *J* = 1.2 Hz, 6H). **^{13}C NMR** (100 MHz, CHLOROFORM-*D*) δ 203.7, 172.2, 138.1, 137.0, 136.2, 130.4, 128.9, 128.7, 128.1, 127.9, 127.2, 126.2,

121.5, 119.2, 118.7, 111.5, 109.7, 64.0, 63.7, 47.2, 46.9, 44.0, 34.6, 28.8, 28.0, 26.2, 25.3, 18.5, 9.3, -5.1, -5.2. **FT-IR** (ATR) cm^{-1}: 3085, 3059, 3029, 2951, 2927, 2882, 2854, 2160, 1680, 1608, 1529, 1464, 1428, 1357, 1254, 1089, 833, 735, 698. **HRMS** (DART) *m/z* calcd for $C_{38}H_{49}N_2O_3Si$ [$M+H^+$]:609.3512; found: 609.3489.

*Compound **22**. tert-butyl (2-(1-benzyl-2-(2-(1-benzyl-3-ethyl-2-oxopyrrolidin-3-yl)-2-oxoethyl)-1H-indol-3-yl)ethyl)carbamate.*

Following the general procedure G. **17** (297 mg, 0.81 mmol, 1.0 equiv), **19c** (370 mg, 1.05 mmol, 1.3 equiv), DLP (583 mg, 1.46 mmol, 1.8 equiv) and DCE (3.3 mL, 0.25 M). The residue was purified by flash column chromatography (hexane/EtAOc 3:1 as eluent). Compound **22** was obtained as a pale-yellow oil (154 mg, 0.26 mmol, 32% yield). **^{1}H NMR** (400 MHz, CHLOROFORM-*D*) δ 7.53 (dd, *J* = 6.2, 2.3 Hz, 1H), 7.28 – 7.12 (m, 6H), 7.10 – 7.00 (m, 5H), 6.87 (d, *J* = 6.2 Hz, 2H), 5.12 (s, 2H), 4.54 (q, *J* = 18.7, 21.7 Hz, 2H), 3.94 (d, *J* = 14.6 Hz, 1H), 3.80 (d, *J* = 18.7 Hz, 1H), 3.29 (q, *J* = 6.5 Hz, 2H), 3.07 – 2.97 (m, 2H), 2.88 – 2.73 (m, 2H), 2.60 (ddd, *J* = 13.1, 7.5, 3.9 Hz, 1H), 2.12 (dq, *J* = 14.7, 7.4 Hz, 1H), 1.86 (dq, *J* = 14.7, 7.4 Hz, 1H), 1.66 (dt, *J* = 13.1, 7.9 Hz, 1H), 1.35 (s, 9H), 0.83 (t, *J* = 7.4 Hz, 3H). **^{13}C NMR** (100 MHz, CHLOROFORM-*D*) δ 204.4, 172.1, 156.1, 137.9, 137.1, 136.1, 130.5, 128.9, 128.8, 128.1, 127.9, 127.8, 127.3, 126.2, 121.8, 119.4, 118.7, 111.8, 109.8, 78.9, 63.9, 47.2, 47.0, 44.0, 34.6, 28.6, 27.90, 25.2, 9.3. **FT-IR** (ATR) cm^{-1}: 3369, 3059, 3030, 2971, 2929, 2878, 1705, 1676, 1495, 1465, 1452, 1390, 1363, 1263, 1249, 1165, 1077, 1061, 1044, 1028, 864, 778, 734, 697. **HRMS** (DART) *m/z* calcd for $C_{37}H_{44}N_3O_4$ [$M+H^+$]:594.3331; found: 594.3311.

*Compound **23**. methyl 1-benzyl-2-(2-(1-benzyl-3-ethyl-2-oxopyrrolidin-3-yl)-2-oxoethyl)-1H-indole-3-carboxylate.*

Following the general procedure G. **17** (300 mg, 0.82 mmol, 1.0 equiv), **19d** (283 mg, 1.07 mmol, 1.3 equiv), DLP (588 mg, 1.47 mmol, 1.8 equiv) and DCE (3.3 mL, 0.25 M). The residue was purified by flash column chromatography (hexane/EtAOc 3:1 as eluent). The product **23** was obtained as a pale-yellow oil (122 mg, 0.24 mmol, 29% yield). **^{1}H NMR** (400 MHz, CHLOROFORM-*D*) δ 8.26 (dt, *J* = 7.9, 1.1 Hz, 1H), 7.41 – 7.24 (m, 11H), 7.10 – 7.06 (m, 2H), 5.33 (q, *J* = 17.0, 18.0 Hz, 2H), 4.99 (m, *J* = 18.4, 1H), 4.86 (d, *J* = 18.4 Hz, 1H), 4.63 (d, *J* = 14.7 Hz, 1H), 4.21 (d, *J* = 14.7 Hz, 1H), 4.00 (s, 3H), 3.29 – 3.17 (m, 2H), 2.81 (ddd, *J* = 13.2, 8.2, 3.9 Hz, 1H), 2.33 (dq, *J* = 7.45, 6.73 Hz, 1H), 2.05 (dq, *J* = 14.7, 7.4 Hz, 1H), 1.88 (ddd, *J* = 13.3, 8.9, 7.0 Hz, 1H), 1.06 (t, *J* = 7.4 Hz, 3H). **^{13}C NMR** (100 MHz, CHLOROFORM-*D*) δ 202.8, 172.6, 166.4, 141.9, 136.8, 136.4, 136.2, 128.9, 128.9, 128.2, 127.8, 127.7, 126.5, 126.3, 122.8, 122.0, 121.9, 110.3, 106.4, 63.5, 50.9, 47.2, 47.0, 44.2, 36.3, 27.9, 25.2, 9.1. **FT-IR** (ATR) cm^{-1}:3060, 3030, 2966, 2946, 2880, 1676, 1541, 1495, 1436, 1352, 1338, 1277, 1238, 1215, 1156, 1135, 1112, 1020, 781, 729, 697. **HRMS** (DART) *m/z* calcd for $C_{32}H_{33}N_2O_4$ [$M+H^+$]:509.2440; found: 509.2435.

*Compound **24**. ethyl 2-(2-(2-(1-benzyl-3-ethyl-2-oxopyrrolidin-3-yl)-2-oxoethyl)-1H-indol-3-yl)acetate.*

Following the general procedure G. **17** (250 mg, 0.68 mmol, 1.0 equiv), **19e** (180 mg, 0.89 mmol, 1.3 equiv), DLP (490 mg, 1.23 mmol, 1.8 equiv) and DCE (2.7 mL, 0.25 M). The residue was purified by flash column chromatography (hexane/EtAOc 3:1 as eluent). The product **24** was obtained as a pale-yellow oil (89 mg, 0.21 mmol, 30% yield). **^{1}H NMR** (400 MHz, acetone-d$_6$) δ 9.96 (s, 1H), 7.53 – 7.49 (m, 1H), 7.36 (dt, *J* = 8.0, 0.9 Hz, 1H), 7.28 – 7.17 (m, 5H), 7.07 (ddd, *J* = 8.1, 7.0, 1.3 Hz, 1H), 7.03 – 6.98 (m, 1H), 4.52 (d, *J* = 17.4 Hz, 1H), 4.47 (s, 2H), 4.14 – 4.00 (m, 3H), 3.79 – 3.57 (m, 2H), 3.27 – 3.16 (m, 2H), 2.67 (ddd, *J* = 13.3, 7.5, 4.8 Hz, 1H), 2.24 – 2.10 (m, 1H), 1.97 – 1.85 (m, 2H), 1.18 (t, *J* = 7.1 Hz, 3H), 0.89 (t, *J* = 7.5 Hz, 3H).**^{13}C NMR** (100 MHz, acetone-d$_6$) δ 204.4, 173.0, 172.1, 137.7, 136.9, 130.8, 129.5, 129.2, 128.8, 128.3, 121.9, 119.6, 119.1, 111.7, 106.9, 64.0, 60.8, 47.4, 44.5, 36.0, 30.7, 28.3, 25.7, 14.6, 9.3. **FT-IR** (ATR) cm^{-1}: 3332, 3030, 2970, 2933, 2878, 1715, 1667, 1493, 1458, 1442, 1301, 1262, 1157, 1029, 927, 740, 700. **HRMS** (DART) *m/z* calcd for $C_{27}H_{31}N_2O_4$ [M+H$^+$]:447.2284; found: 447.2289.

*Compound **25**. 1-benzyl-3-(2-(1-benzyl-1H-indol-2-yl)acetyl)-3-ethylpiperidin-2-one.*

Following the general procedure G. **18** (563 mg, 1.48 mmol, 1.0 equiv), **19a** (400 mg, 1.93 mmol, 1.3 equiv), DLP (1064 mg, 2.67 mmol, 1.8 equiv) and DCE (7.0 mL, 0.25 M). Compound **25** was obtained as a pale-yellow oil (260 mg, 0.56 mmol, 38 % yield).**^{1}H NMR** (400 MHz, CHLOROFORM-*D*) δ 7.59 – 7.55 (m, 1H), 7.26 – 7.05 (m, 12H), 6.92 (d, *J* = 5.2 Hz, 2H), 6.38 (s, 1H), 5.34 (s, 2H), 4.52 (q, *J* = 14.5 Hz, 2H), 4.30 (d, *J* = 17.0 Hz, 1H), 3.98 (d, *J* = 17.0 Hz, 1H), 3.15 (ddd, *J* = 12.0, 8.6, 5.3 Hz, 1H), 3.09 – 3.01 (m, 1H), 2.37 – 2.27 (m, 1H), 2.08 (dq, *J* = 14.8, 7.5 Hz, 1H), 1.96 (dq, *J* = 14.5, 7.4 Hz, 1H), 1.75 – 1.67 (m, 1H), 1.57 (d, *J* = 7.4 Hz, 2H), 0.88 (t, *J* = 7.4 Hz, 3H).**^{13}C NMR** (100 MHz, CHLOROFORM-*D*) δ 205.1, 169.7, 138.0, 137.7, 137.1, 133.3, 128.8, 128.1, 128.1, 127.6, 127.4, 126.1, 121.5, 120.4, 119.8, 109.8, 102.9, 60.9, 51.0, 47.6, 46.9, 36.8, 30.0, 29.9, 27.4, 20.1, 9.2. **FT-IR** (ATR) cm^{-1}: 3028, 2929, 2878, 1709, 1676, 1607, 1492, 1450, 1429, 1353, 1302, 1262, 1197, 1173, 1077, 1044, 1025, 811, 732, 697. HRMS (DART) *m/z* calcd for $C_{31}H_{33}N_2O_2$ [M+H$^+$]: 465.2542; found: 465.2520.

*Compound **26**. 1-benzyl-3-(2-(1-benzyl-3-(2-((tert-butyldimethylsilyl)oxy)ethyl)-1H-indol-2-yl)acetyl)-3-ethylpiperidin-2-one.*

Following the general procedure G. **18** (400 mg, 1.05 mmol, 1.0 equiv), **19b** (496 mg, 1.37 mmol, 1.3 equiv), DLP (756 mg, 1.89 mmol, 1.8 equiv) and DCE (6 mL, 0.25 M). The residue was purified by flash column chromatography (hexane/EtAOc 8:1 as eluent). Compound **26** was obtained as a pale-yellow oil (216 mg, 0.34 mmol, 33% yield). **^{1}H NMR** (400 MHz, CHLOROFORM-*D*) δ 7.62 – 7.58 (m, 1H), 7.33 – 7.07 (m, 11H), 6.90 (d, *J* = 6.3 Hz, 2H), 5.19 (dd, *J* = 17.4, 3.1 Hz, 2H), 4.68 (d, *J* = 14.4 Hz, 1H), 4.47 (d, *J* = 14.4 Hz, 1H), 4.20 (q, *J* = 18.8, 11.4 Hz, 2H), 3.83 – 3.73 (m, 2H), 3.22 – 3.08 (m, 2H), 2.92 (td, *J* = 7.9, 3.1 Hz, 2H), 2.31 – 2.27 (m, 1H), 2.13 (dq, *J* = 14.6, 7.4 Hz, 1H), 1.97 (dq, *J* = 14.7, 7.4 Hz, 1H), 1.72 – 1.65 (m, 1H), 1.59 – 1.50 (m, 2H), 0.90 (d, *J* = 3.2 Hz, 12H), 0.02 (s, 6H). **^{13}C NMR** (100 MHz, CHLOROFORM-*D*) δ 205.3, 169.8, 138.1, 137.1, 137.0, 130.6, 128.9, 128.7, 128.2, 128.1, 127.7, 127.2, 126.2, 121.5, 119.2, 118.7, 111.2, 109.6, 64.0, 60.7, 51.2, 47.7, 46.9,

35.1, 30.0, 28.8, 27.6, 26.2, 20.1, 18.5, 8.3, -5.1. **FT-IR** (ATR) cm^{-1}: 3059, 3029, 2927, 2855, 1711, 1629, 1490, 1464, 1452, 1354, 1253, 1197, 1174, 1089, 1029, 937, 833, 775, 734, 697. **HRMS** (DART) *m/z* calcd for $C_{39}H_{51}N_2O_3Si$ [M+H$^+$]:623.3668; found: 623.3660.

*Compound **27.** tert-butyl (2-(1-benzyl-2-(2-(1-benzyl-3-ethyl-2-oxopiperidin-3-yl)-2-oxoethyl)-1H-indol-3-yl)ethyl)carbamate.*

Following the general procedure G. **18** (316 mg, 0.83 mmol, 1.0 equiv), **19c** (379 mg, 1.08 mmol, 1.3 equiv), DLP (597 mg, 1.49 mmol, 1.8 equiv) and DCE (3.3 mL, 0.25 M). The residue was purified by flash column chromatography (hexane/EtAOc 3:1 as eluent). Compound **27** was obtained as a pale-yellow oil (170 mg, 0.28 mmol, 33% yield). **^{1}H NMR** (400 MHz, CHLOROFORM-*D*) δ 7.62 – 7.58 (m, 1H), 7.33 – 7.07 (m, 12H), 6.91 – 6.88 (m, 2H), 5.18 (q, *J* = 17.3, 5.6 Hz, 2H), 4.77 (s, 1H), 4.65 (d, *J* = 14.4 Hz, 1H), 4.49 (d, *J* = 14.4 Hz, 1H), 4.27 (d, *J* = 18.8 Hz, 1H), 4.10 (d, *J* = 18.8 Hz, 1H), 3.36 (h, *J* = 6.4 Hz, 2H), 3.23 – 3.10 (m, 2H), 2.85 (t, *J* = 6.7 Hz, 2H), 2.33 – 2.29 (m, 1H), 2.16 – 2.07 (m, 1H), 2.02 – 1.93 (m, 1H), 1.73 – 1.66 (m, 1H), 1.63 – 1.54 (m, 2H), 1.42 (s, 9H), 0.90 (t, *J* = 7.4 Hz, 3H). **^{13}C NMR** (100 MHz, CHLOROFORM-*D*) δ 206.0, 169.7, 156.2, 137.9, 137.2, 137.1, 130.7, 128.9, 128.8, 128.3, 127.8, 127.7, 127.3, 126.1, 121.7, 119.4, 118.7, 111.5, 109.7, 78.7, 60.7, 51.2, 47.8, 46.9, 41.1, 35.2, 30.0, 28.6, 27.6, 25.3, 20.2, 9.3. **FT-IR** (ATR) cm^{-1}: 3360, 3058, 3029, 2970, 2930, 2874, 1704, 1624, 1493, 1450, 1358, 1246, 1166, 1073, 1043, 960, 735, 698. **HRMS** (DART) *m/z* calcd for $C_{38}H_{46}N_3O_4$ [M+H$^+$]:608.3488; found: 608.3506.

*Compound **28.** methyl 1-benzyl-2-(2-(1-benzyl-3-ethyl-2-oxopiperidin-3-yl)-2-oxoethyl)-1H-indole-3-carboxylate.*

Following the general procedure G. **18** (307 mg, 0.81 mmol, 1.0 equiv), **19d** (279 mg, 1.05 mmol, 1.3 equiv), DLP (580 mg, 1.45 mmol, 1.8 equiv) and DCE (3.2 mL, 0.25 M). The residue was purified by flash column chromatography (hexane/EtAOc 3:1 as eluent). The product **28** was obtained as a pale-yellow oil (138 mg, 0.26 mmol, 32% yield). **^{1}H NMR** (400 MHz, CHLOROFORM-*D)* δ 8.17 – 8.14 (m, 1H), 7.32 – 7.18 (m, 11H), 6.96 (dd, *J* = 7.6, 1.8 Hz, 2H), 5.25 (q, *J* = 17.3, 4.9 Hz 2H), 4.76 (*J* = 18.5, 10.5 Hz, 2H), 4.62 (s, 2H), 3.89 (s, 3H), 3.23 (dd, *J* = 7.9, 3.5 Hz, 2H), 2.42 – 2.36 (m, 1H), 2.12 (dq, *J* = 13.7, 8.0 Hz, 1H), 1.99 (dq, *J* = 14.6, 7.3 Hz, 1H), 1.78 – 1.64 (m, 3H), 0.93 (t, *J* = 7.4 Hz, 3H). **^{13}C NMR** (100 MHz, CHLOROFORM-*D*) δ 204.6, 170.1, 166.3, 142.0, 137.1, 136.8, 136.5, 128.9, 128.8, 128.2, 127.7, 127.6, 126.5, 126.2, 122.8, 122.0, 121.9, 110.2, 106.1, 60.4, 51.0, 50.9, 47.7, 47.0, 36.9, 29.7, 20.0, 9.2. **FT-IR** (ATR) cm^{-1}: 3059, 3029, 2927, 2855, 1711, 1629, 1490, 1464, 1452, 1354, 1253, 1197, 1174, 1089, 1029, 937, 833, 775, 734, 697. **HRMS** (DART) *m/z* calcd for $C_{33}H_{35}N_2O_4$ [M+H$^+$]:523.2596; found: 523.2572.

*Compound **29.** ethyl 2-(2-(2-(1-benzyl-3-ethyl-2-oxopiperidin-3-yl)-2-oxoethyl)-1H-indol-3-yl)acetate.*

Following the general procedure G. **18** (324 mg, 0.85 mmol, 1.0 equiv), **19e** (225 mg, 1.10 mmol, 1.3 equiv), DLP (612 mg, 1.53 mmol, 1.8 equiv) and DCE (3.4 mL, 0.25 M). The residue was purified by flash column

chromatography (hexane/EtAOc 3:1 as eluent). Compound **29** was obtained as a pale-yellow oil (121 mg, 0.26 mmol, 31% yield). ^{1}**H NMR** (400 MHz, CHLOROFORM-*D*) δ 8.88 (s, 1H), 7.48 (d, *J* = 6.5 Hz, 1H), 7.23 – 7.14 (m, 4H), 7.12 – 6.98 (m, 4H), 4.55 (d, *J* = 2.4 Hz, 2H), 4.26 (d, *J* = 15.8 Hz, 1H), 4.03 (q, *J* = 7.1 Hz, 2H), 3.94 (d, *J* = 15.9 Hz, 1H), 3.67 (q, *J* = 15.6, 6.2 Hz, 2H), 3.16 – 3.04 (m, 2H), 2.28 (ddt, *J* = 12.5, 6.5, 1.9 Hz, 1H), 1.99 (qd, *J* = 7.5, 5.4 Hz, 2H), 1.74 – 1.48 (m, 4H), 1.15 (t, *J* = 7.1 Hz, 3H), 0.83 (t, *J* = 7.4 Hz, 3H). ^{13}**C NMR** (100 MHz, CHLOROFORM-*D*) δ 206.3, 172.0, 170.0, 136.8, 135.6, 129.4, 128.9, 128.2, 128.0, 127.7, 121.8, 119.5, 118.6, 111.0, 106.5, 61.2, 60.8, 51.0, 47.7, 35.8, 30.5, 29.7, 27.5, 20.1, 14.4, 9.3. **FT-IR** (ATR) cm^{-1}: 3311, 3058, 3029, 2935, 2877, 1722, 1613, 1490, 1452, 1353, 1301, 1261, 1199, 1157, 1073, 1029, 957, 738, 699. **HRMS** (DART) *m/z* calcd for $C_{28}H_{33}N_2O_4$ [M+H$^+$]:461.2440; found: 461.2424.

General procedure H. Xanthate synthesis.

*Compound **30**. O-(2-(1-benzyl-1H-indol-2-yl)-1-((S)-1-benzyl-3-ethyl-2-oxopyrrolidin-3-yl)ethyl) S-methyl carbonodithioate*

Step 1. To a solution **20** (897 mg, 1.99 mmol, 1.0 equiv) in EtOH (8.0 mL, 0.25 M) at 0 °C was added $NaBH_4$ (113 mg, 2.98 mmol, 2.0 equiv) and the reaction was allowed to reach room temperature over 6 hours. After the reaction was completed, TLC analysis (hexane/EtOAc 5:1), the solvent was removed under reduced pressure, and the reaction crude was treated with NH_4Cl(ac) and EtOAc, the phases were separated, and the aqueous phase was extracted with EtOAc. The organic phases were put together and dried over anhydrous Na_2SO_4, filtered, and the solvent was removed under reduced pressure. The residue was not purified to the next reaction. Step 2. To a solution of the previously crude in THF (6.2 mL, 0.25 M) at 0 °C was added NaH (80 mg, 2.01 mmol, 1.3 equiv) and the reaction was stirred 30 minutes at 0 °C. CS_2 (139 µL, 2.32 mmol, 1.5 equiv) was added, and the reaction was removed from the ice bath, and let stirred 30 minutes at room temperature. MeI (145 µL, 2.32 mmol, 1.5 equiv) was added, and the reaction was stirred 6 hours at room temperature under nitrogen atmosphere. After the reaction was complete, TLC analysis (hexane/EtOAc 5:1), the crude was diluted with EtOAc (15 mL) and extracted with brine (20 mL). The aqueous phase was extracted with EtOAc. The organic phases were put together and dried over Na_2SO_4, filtered, and the solvent was removed under reduced pressure. The residue was purified by flash column chromatography using silica gel (hexane/EtAOc 7:1). Compound **30** was obtained as mixture of separable diastereomers, both as a yellow oil (651 mg, 1.21 mmol, 78% yield, *dr* 1:1). Diastereomer 1. ^{1}**H NMR** (400 MHz, CHLOROFORM-*D*) δ 7.57 (d, *J* = 7.2 Hz, 1H), 7.33 – 7.22 (m, 10H), 7.12 (dt, *J* = 20.0, 7.1 Hz, 2H), 7.04 (d, *J* = 7.9 Hz, 2H), 6.47 (dd, *J* = 9.6, 3.4 Hz, 1H), 6.44 (s, 1H), 5.49 (s, 2H), 4.46 (dd, 14.5, 22.8 Hz, 2H), 3.32 (dd, *J* = 15.4, 9.7 Hz, 1H), 3.22 – 3.14 (m, 3H), 2.42 (s, 3H), 2.25 (ddd, *J* = 13.3, 8.5, 5.0 Hz, 1H), 1.95 (ddd, *J* = 13.8, 9.0, 6.1 Hz, 1H), 1.88 – 1.77 (m, 1H), 1.68 – 1.56 (m, 2H), 0.91 (d, *J* = 7.4 Hz, 3H). ^{13}**C NMR** (100 MHz, CHLOROFORM-*D*) δ 216.0, 174.5, 138.2, 137.5, 136.4, 135.3, 128.9, 128.8, 128.4, 128.3, 127.7, 127.3, 126.3, 121.2, 120.2, 119.5, 109.7, 103.1, 85.5, 52.9, 46.6, 44.4, 28.4, 28.0, 25.5, 19.3, 8.6. Diastereomer 2. ^{1}**H NMR** (400 MHz, CHLOROFORM-*D*) δ 7.48 (d, *J* = 6.8 Hz, 1H), 7.24 – 7.10 (m,

11H), 7.06 – 6.97 (m, 3H), 6.90 (d, *J* = 6.3 Hz, 2H), 6.37 (s, 1H), 6.23 (dd, *J* = 8.4, 4.0 Hz, 1H), 5.29 (q, 16.7 Hz, 2H), 4.31 (q, 15.4, 32 Hz, 2H), 3.31 (dd, *J* = 15.7, 4.1 Hz, 1H), 3.15 – 2.96 (m, 3H), 2.36 (s, 3H), 2.06 (ddd, *J* = 13.9, 9.1, 5.3 Hz, 1H), 1.78 – 1.66 (m, 2H), 1.45 (dq, *J* = 14.5, 7.4 Hz, 1H), 0.78 (t, *J* = 7.4 Hz, 3H). **^{13}C NMR** (100 MHz, CHLOROFORM-*D*) δ 214.9, 174.3, 138.0, 137.5, 136.5, 135.5, 128.9, 128.8, 128.3, 128.2, 127.7, 127.4, 126.2, 121.3, 120.2, 119.6, 109.7, 103.2, 84.1, 53.4, 47.0, 46.8, 44.0, 27.7, 26.1, 24.6, 19.2, 8.8. **FT-IR** (ATR) cm^{-1}: 3059, 3028, 2965, 2922, 2877, 2241, 1678, 1494, 1452, 1352, 1314, 1263, 1214, 1181, 1054, 1028, 964, 907, 724, 697, 607. **HRMS** (DART) *m/z* calcd for $C_{33}H_{35}N_2O_2S_2$ [M+H$^+$]:543.2133; found: 543.2139.

*Compound **31**. O-(2-(1-benzyl-1H-indol-2-yl)-1-(1-benzyl-3-ethyl-2-oxopiperidin-3-yl)ethyl) S-methyl carbonodithioate.*

Following the general procedure H. Step 1, **25** (2185 mg, 4.70 mmol, 1.0 equiv), $NaBH_4$ (355 mg, 9.40 mmol, 2.0 equiv) and EtOH (19 mL, 0.25 M). Step 2, THF (18 mL,0.25 M), NaH (244 mg, 6.11 mmol, 1.3 equiv), CS_2 (421 µL, 7.05 mmol, 1.5 equiv) and MeI (439 µL, 7.05 mmol, 1.5 equiv). Compound **31** was obtained as mixture of separable diastereomers, both as a yellow oil (1881 mg, 3.38 mmol, 72% yield, *dr* 1:1). Diastereomer 1. **^{1}H NMR** (400 MHz, ACETONE-*D*$_6$) δ 7.48 – 7.42 (m, 1H), 7.29 – 7.20 (m, 9H), 7.06 – 6.93 (m, 4H), 6.74 (dd, *J* = 10.5, 2.5 Hz, 1H), 6.35 (s, 1H), 5.74 – 5.44 (m, 2H), 4.61 – 4.36 (m, 2H), 3.36 – 3.25 (m, 3H), 3.03 (dd, *J* = 14.8, 2.4 Hz, 1H), 2.46 (s, 3H), 2.27 (dd, *J* = 7.3, 5.5 Hz, 1H), 1.91 – 1.78 (m, 4H), 1.65 (dd, *J* = 13.7, 7.4 Hz, 1H), 0.94 (t, *J* = 7.5 Hz, 3H). **^{13}C NMR** (100 MHz, ACETONE-*D*$_6$) δ 215.4, 170.9, 139.0, 137.8, 137.6, 135.6, 128.6, 128.3, 128.1, 127.2, 127.0, 126.3, 120.9, 119.8, 119.2, 102.8, 86.1, 50.0, 49.7, 46.9, 46.1, 31.1, 26.8, 20.6, 18.0, 8.9. Diastereomer 2. **^{1}H NMR** (400 MHz, ACETONE-*D*$_6$) δ 7.48 (d, *J* = 7.0 Hz, 1H), 7.32 – 7.20 (m, 9H), 7.06 – 6.96 (m, 4H), 6.50 (dd, *J* = 8.9, 4.0 Hz, 1H), 6.44 (s, 1H), 5.59 – 5.40 (m, 2H), 4.62 (d, *J* = 14.6 Hz, 1H), 4.48 (d, *J* = 14.6 Hz, 1H), 3.45 (dd, *J* = 15.7, 3.7 Hz, 1H), 3.30 – 3.13 (m, 3H), 2.45 (s, 3H), 2.03 – 1.69 (m, 5H), 1.54 – 1.45 (m, 1H), 0.90 (d, *J* = 7.4 Hz, 3H).**^{13}C NMR** (100 MHz, ACETONE-*D*$_6$) δ 214.3, 170.5, 138.6, 138.0, 137.5, 136.1, 128.6, 128.5, 128.4, 128.0, 127.2, 127.1, 126.2, 120.9, 119.8, 119.3, 109.7, 102.8, 85.9, 50.4, 50.0, 47.3, 46.4, 28.5, 27.3, 27.2, 20.4, 18.2, 8.9.**FT-IR** (ATR) (mixture) cm^{-1}: 3058, 3029, 2960, 2928, 1619, 1543, 1492, 1450, 1426, 1357, 1314, 1258, 1223, 1180, 1160, 1145, 1053, 1013, 960, 907, 746, 725, 694. **HRMS** (DART) (mixture) *m/z* calcd for $C_{33}H_{37}N_2O_2S_2$ [M+H$^+$]:557.2296; found: 557.2322.

*Compound **32**. O-(2-(1-benzyl-3-(2-((tert-butyldimethylsilyl)oxy)ethyl)-1H-indol-2-yl)-1-(1-benzyl-3-ethyl-2-oxopiperidin-3-yl)ethyl) S-methyl carbonodithioate.*

Following the general procedure H. Step 1, **26** (1517 mg, 2.43 mmol, 1 equiv), $NaBH_4$ (184 mg, 4.87 mmol, 2.0 equiv) and EtOH (10 mL, 0.25 M). Step 2, THF (10 mL, 0.25 M), NaH (126 mg, 3.15 mmol, 1.3 equiv), CS_2 (217 µL, 3.64 mmol, 1.5 equiv) and MeI (226 µL, 3.64 mmol, 1.5 equiv). Compound **32** was obtained as mixture of separable diastereomers, both as a yellow oil (1459 mg, 2.04 mmol, 84% yield, *dr* 1:1). Diastereomer 1. **^{1}H NMR** (400 MHz, CHLOROFORM-*D*) δ 7.53 (ddd, *J* = 7.6, 1.4, 0.7 Hz, 1H), 7.29 – 7.05 (m, 13H), 6.74 (dd, *J* = 11.5, 1.9 Hz, 1H), 5.68 (d, *J* = 17.4 Hz, 1H), 5.49 (d, *J* = 17.4 Hz, 1H), 4.66 (d, *J* = 14.4 Hz, 1H), 4.51 (d, *J* = 14.4 Hz, 1H),

3.94 (ddd, *J* = 9.9, 8.2, 5.7 Hz, 1H), 3.80 (dt, *J* = 9.9, 7.7 Hz, 1H), 3.39 (dd, *J* = 14.9, 11.4 Hz, 1H), 3.28 – 3.17 (m, 2H), 3.06 – 2.94 (m, 2H), 2.85 (dd, *J* = 14.9, 1.9 Hz, 1H), 2.36 (s, 3H), 2.30 – 2.23 (m, 1H), 2.03 – 1.76 (m, 4H), 1.68 (dq, *J* = 14.8, 7.4 Hz, 1H), 1.00 (t, *J* = 7.5 Hz, 3H), 0.90 (s, 9H), 0.03 (d, *J* = 14.5 Hz, 6H). **^{13}C NMR** (100 MHz, CHLOROFORM-*D*) δ 214.9, 171.5, 138.8, 137.2, 137.1, 132.1, 128.8, 128.6, 128.3, 128.0, 127.5, 127.0, 126.4, 121.1, 118.7, 118.3, 111.3, 109.6, 86.5, 64.0, 50.7, 50.0, 47.2, 46.4, 31.5, 28.6, 27.2, 26.5, 26.1, 21.1, 19.2, 18.5, 9.5, -5.1, -5.1. Diastereomer 2. **^{1}H NMR** (400 MHz, CHLOROFORM-*D*) δ 7.52 – 7.48 (m, 1H), 7.29 – 7.20 (m, 8H), 7.15 – 7.12 (m, 1H), 7.10 – 7.02 (m, 2H), 6.99 – 6.95 (m, 2H), 6.52 (dd, *J* = 8.1, 5.9 Hz, 1H), 5.44 (d, *J* = 17.4 Hz, 1H), 5.35 (d, *J* = 17.4 Hz, 1H), 4.62 (d, *J* = 14.5 Hz, 1H), 4.47 (d, *J* = 14.5 Hz, 1H), 3.88 (ddd, *J* = 9.9, 8.4, 6.1 Hz, 1H), 3.79 (dt, *J* = 10.0, 7.5 Hz, 1H), 3.22 – 3.18 (m, 4H), 3.04 – 2.90 (m, 2H), 2.30 (s, 3H), 2.12 – 2.03 (m, 2H), 1.98 – 1.91 (m, 1H), 1.86 – 1.71 (m, 2H), 1.54 (dq, *J* = 13.1, 7.3 Hz, 1H), 0.97 (t, *J* = 7.4 Hz, 3H), 0.89 (s, 10H), 0.02 (d, *J* = 4.6 Hz, 6H). **^{13}C NMR** (100 MHz, CHLOROFORM-*D*) δ 213.8, 171.0, 138.4, 137.5, 137.0, 132.2, 128.8, 128.6, 128.3, 128.1, 127.4, 127.2, 126.2, 121.2, 118.9, 118.4, 111.6, 109.5, 85.5, 63.9, 51.1, 50.2, 47.5, 46.9, 29.0, 28.8, 27.4, 26.2, 24.9, 20.7, 19.3, 18.5, 9.7, -5.1, -5.1. **FT-IR** (ATR) (mixture) cm^{-1}: 3029, 2950, 2928, 2855, 1633, 1491, 1465, 1355, 1251, 1204, 1179, 1092, 1048, 963, 920, 833, 775, 731, 697. **HRMS** (DART) (mixture) *m/z* calcd for $C_{41}H_{55}N_2O_3S_2Si$ [M+H$^+$]:716.3419; found: 716.3426.

General procedure I. Barton-McCombie Deoxygentation.

*Compound **33**. 1-benzyl-3-(2-(1-benzyl-1H-indol-2-yl)ethyl)-3-ethylpyrrolidin-2-one.*

To a solution of xanthate **30** (615 mg, 1.13 mmol, 1.0 equiv) in PhMe (4.5 mL, 0.25 M) was added *n*-Bu_3SnH (457 µL, 1.70 mmol, 1.5 equiv) and AIBN (19 mg, 0.11 mmol, 0.1 equiv), the resulting mixture was degassed by three consecutive freeze-pump-thaw cycles using liquid nitrogen and back filled with pure argon. The reaction mixture was refluxed and stirred for 1.5 hours under argon atmosphere. After the reaction was completed, TLC analysis (hexane/EtOAc 8:1), the crude was cooled to room temperature and the solvent was removed under reduced pressure. The residue was purified by flash column chromatography using silica gel (hexane/EtOAc 8:1). Compound **33** was obtained as a pale-yellow oil (432 mg, 0.98 mmol, 88% yield). **^{1}H NMR** (400 MHz, CHLOROFORM-*D*) δ 7.55 (dd, *J* = 6.4, 2.6 Hz, 1H), 7.21 (m, 10H), 7.07 (m, 2H), 6.97 – 6.89 (m, 2H), 6.32 (s, 1H), 5.29 (d, *J* = 4.1 Hz, 2H), 4.39 (q, *J* = 14.5 Hz, 2H), 3.10 – 2.98 (m, 2H), 2.75 – 2.63 (m, 1H), 2.61 – 2.51 (m, 1H), 1.96 (td, *J* = 12.9, 12.2, 4.6 Hz, 1H), 1.89 – 1.69 (m, 3H), 1.64 – 1.50 (m, 2H), 0.83 (t, *J* = 7.4 Hz, 3H). **^{13}C NMR** (100 MHz, CHLOROFORM-*D*) δ 177.5, 140.9, 138.1, 137.3, 136.8, 128.8, 128.3, 128.2, 127.6, 127.3, 126.1, 121.0, 119.9, 119.6, 99.5, 47.9, 46.8, 46.4, 43.7, 35.9, 29.7, 27.5, 21.9, 8.7. **FT-IR** (ATR) cm^{-1}: 3058, 3029, 2962, 2919, 2240, 1673, 1604, 1545, 1494, 1451, 1410, 1353, 1312, 1261, 1200, 116+4, 909, 724, 696. **HRMS** (DART) *m/z* calcd for $C_{30}H_{33}N_2O$ [M+H$^+$]:437.2598; found: 437.2592.

*Compound **34**. 1-benzyl-3-(2-(1-benzyl-1H-indol-2-yl)ethyl)-3-ethylpiperidin-2-one.*

Following the general procedure I. **31** (1652 mg, 2.96 mmol, 1.0 equiv), PhMe (12 mL, 0.25 M), *n*-Bu_3SnH (1.2 mL, 4.45 mmol, 1.5 equiv) and AIBN (48 mg, 0.29 mmol, 0.1 equiv). Compound **34** was obtained as a pale-

yellow oil (1239 mg, 2.75 mmol, 93% yield). **^{1}H NMR** (400 MHz, CHLOROFORM-*D*) δ 7.68 – 7.64 (m, 1H), 7.35 – 7.26 (m, 9H), 7.21 – 7.14 (m, 2H), 7.05 (dd, *J* = 7.8, 1.6 Hz, 2H), 6.44 (s, 1H), 5.45 (q, *J* = 16.5, 5.4 Hz, 2H), 4.62 (q, *J* = 14.5, 8.6 Hz, 2H), 3.23 (h, *J* = 5.4 Hz, 2H), 2.88 – 2.68 (m, 2H), 2.22 (td, *J* = 12.9, 4.4 Hz, 1H), 1.95 – 1.64 (m, 8H), 0.95 (t, *J* = 7.5 Hz, 3H). **^{13}C NMR** (100 MHz, CHLOROFORM-*D*) δ 174.4, 141.5, 138.4, 137.8, 137.4, 128.8, 128.7, 128.3, 128.2, 127.4, 127.2, 126.2, 120.9, 119.9, 119.6, 109.5, 99.5, 50.7, 47.7, 46.4, 45.1, 38.2, 31.5, 29.4, 22.3, 19.8, 8.6. **FT-IR** (ATR) cm^{-1}: 3057, 3028, 2935, 2871, 1624, 1545, 1490, 1453, 1349, 1312, 1257, 1194, 1165, 1076, 1024, 910, 727, 697. **HRMS** (DART) *m/z* calcd for $C_{31}H_{35}N_2O$ [M+H$^+$]:451.2749; found: 451.2744.

*<u>Compound **35**. 1-benzyl-3-(2-(1-benzyl-3-(2-((tert-butyldimethylsilyl)oxy)ethyl)-1H-indol-2-yl)ethyl)-3-ethylpiperidin-2-one.</u>*

Following the general procedure I. **32** (1217 mg, 1.70 mmol, 1.0 equiv), PhMe (7.0 mL, 0.25 M), *n*-Bu_3SnH (687 µL, 2.55 mmol, 1.5 equiv) and AIBN (28 mg, 0.17 mmol, 0.1 equiv). Compound **35** was obtained as a pale-yellow oil (998 mg, 1.64 mmol, 97% yield). **^{1}H NMR** (400 MHz, CHLOROFORM-*D*) δ 7.63 – 7.60 (m, 1H), 7.27 – 7.19 (m, 9H), 7.16 – 7.11 (m, 2H), 7.02 – 6.99 (m, 2H), 5.55 (d, *J* = 17.3 Hz, 1H), 5.40 (d, *J* = 17.3 Hz, 1H), 4.58 (q, *J* = 14.6, 5.0 Hz, 2H), 3.88 (t, *J* = 7.7 Hz, 2H), 3.21 – 3.13 (m, 2H), 3.04 (t, *J* = 7.0 Hz, 2H), 2.89 (td, *J* = 13.6, 4.9 Hz, 1H), 2.58 (dd, *J* = 26.9, 3.8 Hz, 1H), 2.06 (td, *J* = 13.3, 3.7 Hz, 1H), 1.89 – 1.79 (m, 4H), 1.73 – 1.53 (m, 3H), 0.94 (s, 9H), 0.90 (t, *J* = 7.4 Hz, 3H), 0.08 (d, *J* = 4.2 Hz, 6H). **^{13}C NMR** (100 MHz, CHLOROFORM-*D*) δ 174.3, 138.8, 137.9, 137.7, 136.8, 128.7, 128.6, 128.3, 128.2, 127.3, 127.0, 126.2, 121.0, 119.1, 118.3, 64.2, 50.6, 47.6, 46.3, 45.2, 39.4, 31.8, 29.2, 28.8, 26.2, 20.3, 19.8, 18.5, 8.6, -5.1, -5.1. **FT-IR** (ATR) cm^{-1}: 3059, 3029, 2929, 2856, 1627, 1491, 1465, 1355, 1253, 1194, 1090, 910, 834, 776, 731, 697. **HRMS** (DART) *m/z* calcd for $C_{39}H_{53}N_2O_2Si$ [M+H$^+$]:609.3876; found: 609.3872.

<u>General procedure J. *N*-Debenzylation.</u>

*<u>Compound **36**.</u>* *<u>3-(2-(1H-indol-2-yl)ethyl)-3-ethylpyrrolidin-2-one.</u>*

To a solution of **33** (384 mg, 0.88 mmol, 1.0 equiv) in freshly distilled THF (3 mL, 0.3 M) was cooled to –78 °C in acetone/dry ice bath. Freshly condensed ammonia (3 mL, 0.3 M) was slowly added to the solution and the system was stirred at –78 °C for 10 minutes. Sodium in small pieces (202 mg, 8.79 mmol, 10 equiv) was added to the mixture, and the reaction was vigorously stirred for 1.5 hours at –78 °C. After the reaction was completed, TLC analysis (EtOAc 100%), EtOH (1.0 mL, 17.59 mmol, 20 equiv) and NH_4Cl (470 mg, 8.79 mmol, 10 equiv) was added to the mixture, the cryogenic bath was removed, the reaction was opened and allowed to reach room temperature. The reaction was diluted with EtOAc (15 mL) and extracted with brine (15 mL). The aqueous phase was extracted with EtOAc (15 mL). The organic phases were put together and dried over Na_2SO_4, filtered, and the solvent was removed under reduced pressure. The residue was purified by flash column chromatography using silica gel (hexane/EtAOc 1:2). Compound **36** was obtained as a white solid (182 mg, 0.71 mmol, 81% yield), mp 160-163 °C. **^{1}H NMR** (400 MHz, CHLOROFORM-*D*) δ 8.49 (s, 1H), 7.51 (dd, *J* =

7.2, 1.5 Hz, 1H), 7.28 (d, *J* = 8.0 Hz, 1H), 7.07 (dtd, *J* = 18.0, 7.1, 1.3 Hz, 2H), 6.28 (s, 1H), 6.21 (s, 1H), 3.31 (t, *J* = 7.1 Hz, 2H), 2.90 – 2.82 (m, 1H), 2.76 – 2.65 (m, 1H), 2.07 (t, *J* = 7.9 Hz 2H), 2.04 – 1.95 (m, 1H), 1.94 – 1.86 (m, 1H), 1.69 – 1.56 (m, 2H), 1.27 (d, *J* = 4.8 Hz, 1H), 0.95 (t, *J* = 7.5 Hz, 3H).**^{13}C NMR** (100 MHz, CHLOROFORM-*D*) δ 181.9, 139.6, 136.2, 121.1, 119.8, 119.6, 1107. 99.3, 47.2, 39.9, 35.9, 30.3, 29.6, 23.5, 8.8. **FT-IR** (ATR) cm^{-1}:3279, 2968, 2939, 2876, 2826, 1679, 1646, 1455, 1417, 1377, 1282, 1217, 1055, 773, 749, 700. **HRMS** (DART) *m/z* calcd for $C_{16}H_{21}N_2O$ [M+H$^+$]:257.1658; found: 257.1653.

*<u>Compound **37**. 3-(2-(1H-indol-2-yl)ethyl)-3-ethylpiperidin-2-one.</u>*

Following the general procedure J. To a solution of **34** (1452 mg, 3.22 mmol, 1.0 equiv), Na (740 mg, 32.2 mmol, 10 equiv), EtOH (9.8 mL, 64.4 mmol, 20 equiv), NH_4Cl (1723 mg, 32.2 mmol, 10 equiv), THF (11 mL, 0.3 M) and NH_3 (11 mL, 0.3 M). Compound **37** was obtained as a white solid (773 mg, 2.86 mmol, 89% yield), mp 170-172 °C. **^{1}H NMR** (400 MHz, CHLOROFORM-*D*) δ 8.56 (s, 1H), 7.56 (d, *J* = 7.6 Hz, 1H), 7.35 – 7.31 (m, 1H), 7.13 (dt, *J* = 19.3, 7.2 Hz, 2H), 6.26 (s, 1H), 6.06 (s, 1H), 3.39 – 3.35 (m, 2H), 2.92 (ddd, *J* = 14.5, 11.1, 5.9 Hz, 1H), 2.76 (ddd, *J* = 15.0, 11.1, 4.6 Hz, 1H), 2.25 – 2.15 (m, 1H), 1.94 – 1.81(m, 7H), 1.70 (dq, *J* = 14.6, 7.4 Hz, 1H), 0.98 (t, *J* = 7.5 Hz, 3H). **^{13}C NMR** (100 MHz, CHLOROFORM-*D*) δ 177.2, 140.1, 136.2, 128.8, 121.0, 119.8, 119.5, 110.7, 99.3, 45.3, 42.9, 37.8, 31.3, 29.3, 23.9, 19.8, 8.6. **FT-IR** (ATR) cm^{-1}: 3279, 3081, 3051, 2943, 2870, 1640, 1585, 1487, 1454, 1415, 1349, 1328, 1283, 1202, 1148, 1103, 1048, 1007 792, 735. **HRMS** (DART) *m/z* calcd for $C_{17}H_{23}N_2O$ [M+H$^+$]:271.1810; found: 271.1805.

*<u>Compound **38**. 3-(2-(3-(2-((tert-butyldimethylsilyl)oxy)ethyl)-1H-indol-2-yl)ethyl) ethylpiperidin-2-one.</u>*

Following the general procedure J**. 35** (610 mg, 1.00 mmol, 1.0 equiv), Na (230 mg, 10.0 mmol, 10 equiv), EtOH (1.2 mL, 20.0 mmol, 20 equiv), NH_4Cl (535 mg, 10 mmol, 10 equiv) THF (4.0 mL, 0.3 M) and NH_3 (4.0 mL, 0.3 M). Compound **38** was obtained as a white solid (365 mg, 0.85 mmol, 85% yield).**^{1}H NMR** (400 MHz, CHLOROFORM-*D*) δ 8.44 (s, 1H), 7.50 – 7.46 (m, 1H), 7.27 – 7.23 (m, 1H), 7.11 – 7.03 (m, 2H), 5.95 (s, 1H), 3.77 (t, *J* = 7.3, 2H), 3.35 – 3.30 (m, 2H), 2.95 – 2.86 (m, 3H), 2.58 (ddd, *J* = 14.7, 10.9, 4.3 Hz, 1H), 2.09 (ddd, *J* = 13.6, 10.9, 4.3 Hz, 1H), 1.91 – 1.60 (m, 7H), 0.90 (s, 12H), 0.03 (d, *J* = 1.5 Hz, 6H). **^{13}C NMR** (100 MHz, CHLOROFORM-*D*) δ 177.3, 136.6, 135.6, 128.7, 121.0, 119.0, 118.2, 110.7, 107.4, 64.2, 45.5, 42.9, 38.2, 31.4, 29.1, 28.4, 26.2, 21.6, 19.8, 18.6, 8.6, -5.1. **FT-IR** (ATR) cm^{-1}: 3524, 3109, 3053, 2931, 2857, 2738, 2164, 2014, 1646, 1605, 1489, 1461, 1356, 1325, 1251, 1088, 1064, 1006, 857, 835, 774, 738. **HRMS** (DART) *m/z* calcd for $C_{25}H_{41}N_2O_2Si$ [M+H$^+$]:429.2937; found: 429.2916.

*<u>Compound **39**. tert-butyl 2-(2-(1-(tert-butoxycarbonyl)-3-ethyl-2-oxopiperidin-3-yl)ethyl)-1H-indole-1-carboxylate.</u>*

To a solution of **37** (653 mg, 2.41 mmol, 1.0 equiv) in freshly distilled THF (12 mL, 0.2 M) were added TEA (1.0 mL, 7.24 mmol, 3.0 equiv), DMAP (59 mg, 0.483 mmol, 0.2 equiv) and Boc_2O (1518 mg, 7.24 mmol, 3.0 equiv). The reaction mixture was heated to 60 °C and stirred for 8 hours. After the reaction was completed, TLC

analysis (hexane/EtOAc 7:1), the crude was cooled to room temperature and the solvent was removed under reduced pressure. The residue was purified by flash column chromatography using silica gel (hexane/EtAOc 7:1 as eluent). Compound **39** was obtained as a pale-yellow oil (1077 mg, 2.29 mmol, 95% yield). **^{1}H NMR** (400 MHz, CHLOROFORM-*D*) δ 8.04 (dd, *J* = 8.3, 1.2 Hz, 1H), 7.45 – 7.41 (m, 1H), 7.23 – 7.14 (m, 2H), 6.38 (s, 1H), 3.64 (td, *J* = 5.2, 1.4 Hz, 2H), 3.10 – 2.93 (m, 2H), 2.01 (dddd, *J* = 38.4, 13.3, 11.5, 5.1 Hz, 2H), 1.91 – 1.77 (m, 6H), 1.69 (s, 12H), 1.52 (s, 9H), 0.92 (t, *J* = 7.4 Hz, 3H). **^{13}C NMR** (100 MHz, CHLOROFORM-*D*) δ 176.8, 154.0, 150.7, 142.5, 136.6, 129.5, 123.3, 122.7, 119.9, 115.6, 106.9, 83.8, 82.5, 48.2, 47.3, 36.6, 31.0, 30.7, 28.4, 28.2, 24.9, 20.3, 8.6. **FT-IR** (ATR) cm^{-1}: 2974, 2935, 2880, 1765, 1712, 1593, 1567, 1453, 1367, 1325, 1297, 1275, 1251, 1143, 1114, 1084, 851, 801, 742. **HRMS** (DART) *m/z* calcd for $C_{27}H_{39}N_2O_5$ [M+H$^+$]:471.2859; found: 471.2870.

*Compound **40**. 4a-ethyl-2,3,4,4a,5,6,7,11c-octahydro-1H-pyrido[3,2-c]carbazole.*

To a solution of **39** (841 mg, 1.78 mmol, 1.0 equiv) in freshly distilled DCM (9 mL, 0.2 M) was cooled to –78 °C in acetone/dry ice bath. A solution of $LiEt_3BH$ 1.0 M in THF (2.70 mL, 2.7 mmol, 1.5 equiv) was added and the solution was stirred 1.5 hours at –78 °C. TFA (2.8 mL, 35.74 mmol, 20.0 equiv) was added slowly at –78 °C and the reaction was allowed to reach room temperature for 10 hours. After the reaction was completed, TLC analysis (hexane/acetone 4:1 + 5% TEA), the volatiles were removed under reduced pressure and the residue was treated with a $NaHCO_3$(ac) until pH=9-10 and extracted with AcOEt twice. The organic phases were put together and dried over anhydrous Na_2SO_4, filtered, and the solvent was removed under reduce pressure. The residue was purified by flash column chromatography using silica gel (hexane/acetone 4:1 + 5% TEA). Compound **40** was obtained as a white solid (310 mg, 1.22 mmol, 69% yield), mp 178-181 °C. **^{1}H NMR** (400 MHz, CHLOROFORM-*D*) δ 7.97 (s, 1H), 7.59 – 7.54 (m, 1H), 7.24 – 7.19 (m, 1H), 7.07 (tt, *J* = 7.2, 5.5 Hz, 2H), 3.71 (s, 1H), 3.03 (d, *J* = 12.4 Hz, 1H), 2.78 (td, *J* = 11.6, 3.2 Hz, 1H), 2.65 (dd, *J* = 8.3, 3.9 Hz, 2H), 2.34 (dt, *J* = 13.5, 8.7 Hz, 1H), 1.85 – 1.78 (m, 1H), 1.68 – 1.42 (m, 6H), 1.12 (dq, *J* = 14.5, 7.4 Hz, 1H), 0.85 (t, *J* = 7.5 Hz, 3H). **^{13}C NMR** (100 MHz, CHLOROFORM-*D*) δ 136.3, 134.3, 127.6, 121.0, 119.4, 117.8, 112.5, 110.6, 56.8, 46.3, 34.6, 34.2, 29.6, 24.4, 23.0, 20.3, 7.7. **FT-IR** (ATR) cm^{-1}: 3143, 3103, 3055, 2926, 2848, 2823, 2785, 2742, 1622, 1590, 1569, 1451, 1377, 1348, 1303, 1228, 1187, 1165, 1113, 1011, 980, 897, 871, 834, 814, 779, 734. **HRMS** (DART) *m/z* calcd for $C_{17}H_{23}N_2$ [M+H$^+$]:255.1861; found: 255.1862.

*Compound **41**. 2-(4a-ethyl-2,3,4,4a,5,6,7,11c-octahydro-1H-pyrido[3,2-c]carbazol-1-yl)ethan-1-ol.*

To a solution of **40** (285 mg, 1.12 mmol, 1.0 equiv) in EtOH (11.2 mL, 0.1 M) were added 2-bromoethanol (1.2 mL, 16.8 mmol, 15 equiv) and Na_2CO_3 (1781 mg, 16.8 mmol, 15 equiv). The mixture was heated at 65 °C for 12 hours. After the reaction was completed, TLC analysis (hexane/acetone 4:1 + 5% TEA), the crude was cooled to room temperature and the solvent was removed under reduced pressure. The residue was treated with water (15 mL) and EtOAc (15 mL), the phases were separated, and the aqueous phase was extracted with EtOAc (15 mL). The organic phases were put together and dried over anhydrous Na_2SO_4, filtered, and the

solvent was removed under reduced pressure. The residue was purified by flash column chromatography (hexane/acetone 4:1 + 5% TEA). Compound **41** was obtained as a yellow oil (271 mg, 0.91 mmol, 81% yield). **^{1}H NMR** (400 MHz, CHLOROFORM-*D*) δ 8.05 (s, 1H), 7.44 (ddt, *J* = 7.5, 4.1, 2.0 Hz, 1H), 7.27 – 7.21 (m, 1H), 7.14 – 7.03 (m, 2H), 3.55 – 3.48 (m, 1H), 3.25 (s, 1H), 3.23 – 3.06 (m, 3H), 2.80 – 2.61 (m, 3H), 2.24 (td, *J* = 11.7, 2.6 Hz, 2H), 1.85 (t, *J* = 13.3 Hz, 1H), 1.75 (ddt, *J* = 13.5, 4.3, 2.0 Hz, 1H), 1.60 (ddq, *J* = 12.9, 5.0, 2.6 Hz, 1H), 1.50 – 1.36 (m, 2H), 1.19 (dt, *J* = 14.9, 7.5 Hz, 1H), 1.00 – 0.91 (m, 1H), 0.75 (t, *J* = 7.5 Hz, 3H). **^{13}C NMR** (100 MHz, CHLOROFORM-*D*) δ 136.2, 135.6, 121.0, 119.6, 117.9, 110.7, 110.7, 63.1, 58.1, 54.3, 52.4, 37.2, 34.8, 29.7, 24.4, 22.1, 20.5, 8.0. **FT-IR** (ATR) cm^{-1}: 3399, 3212, 3180, 3055, 2926, 2874, 2851, 1620, 1583, 1459, 1375, 1331, 1306, 1263, 1232, 1193, 1167, 1118, 1037, 989, 944, 922, 885, 865, 777, 734. **HRMS** (DART) *m/z* calcd for $C_{19}H_{27}N_2O$ [M+H$^+$]:299.2123; found: 299.2126.

*Compound **42**. 1,2-Dehydroaspidospermidine.*

To a solution of **41** (249 mg, 0.83 mmol, 1.0 equiv) in THF (8.3 mL, 0.1 M) was added portion-wise tBuOK (374 mg, 3.33 mmol, 4.0 equiv) at 0 °C. After stirring for 10 minutes, TsCl (239 mg, 1.25 mmol, 1.5 equiv) was added, and the reaction was allowed to warm to room temperature for 1.5 hours. After the reaction was completed, TLC analysis (hexane/acetone 4:1 + 5% TEA), the crude was diluted with EtOAc (15 mL) and extracted with brine (15 mL). The aqueous phase was extracted with EtOAc (15 mL). The organic phases were put together and dried over anhydrous Na_2SO_4, filtered, and the solvent was removed under reduced pressure. The residue was purified by flash column chromatography using silica gel (hexane/Acetone 4:1 + 10% TEA). Compound **1** was obtained as a pale-yellow oil (103 mg, 0.37 mmol, 45% yield). **^{1}H NMR** (400 MHz, CHLOROFORM-*D*) δ 7.43 (d, *J* = 7.7 Hz, 1H), 7.27 – 7.17 (m, 2H), 7.07 (td, *J* = 7.4, 1.1 Hz, 1H), 3.12 – 3.06 (m, 2H), 3.05 – 2.98 (m, 1H), 2.67 (ddd, *J* = 14.1, 10.4, 3.5 Hz, 1H), 2.50 (ddd, *J* = 11.5, 8.5, 5.7 Hz, 1H), 2.42 – 2.33 (m, 1H), 2.31 (s, 1H), 2.15 – 2.03 (m, 2H), 1.77 (qt, *J* = 13.4, 4.7 Hz, 1H), 1.59 – 1.43 (m, 3H), 1.42 – 1.34 (m, 1H), 0.92 (td, *J* = 13.5, 5.0 Hz, 1H), 0.66 – 0.47 (m, 2H), 0.46 – 0.33 (m, 3H). **^{13}C NMR** (100 MHz, CHLOROFORM-*D*) δ 192.5, 154.7, 147.3, 127.6, 125.2, 121.1, 120.2, 79.1, 61.4, 54.7, 52.1, 36.6, 35.3, 33.3, 29.8, 27.3, 23.9, 22.2, 7.4. **FT-IR** (ATR) cm^{-1}: 2928, 2853, 2774, 2715, 2676, 1694, 1610, 1575, 1451, 1371, 1342, 1322, 1247, 1192, 1172, 1141, 1121, 1012, 770, 746. **HRMS** (DART) *m/z* calcd for $C_{19}H_{25}N_2$ [M+H$^+$]:281.2017; found: 281.2011.

3. NMR-SPECTRA

*Compound **9**. ^{1}H NMR (400 MHz, CHLOROFORM-D).*

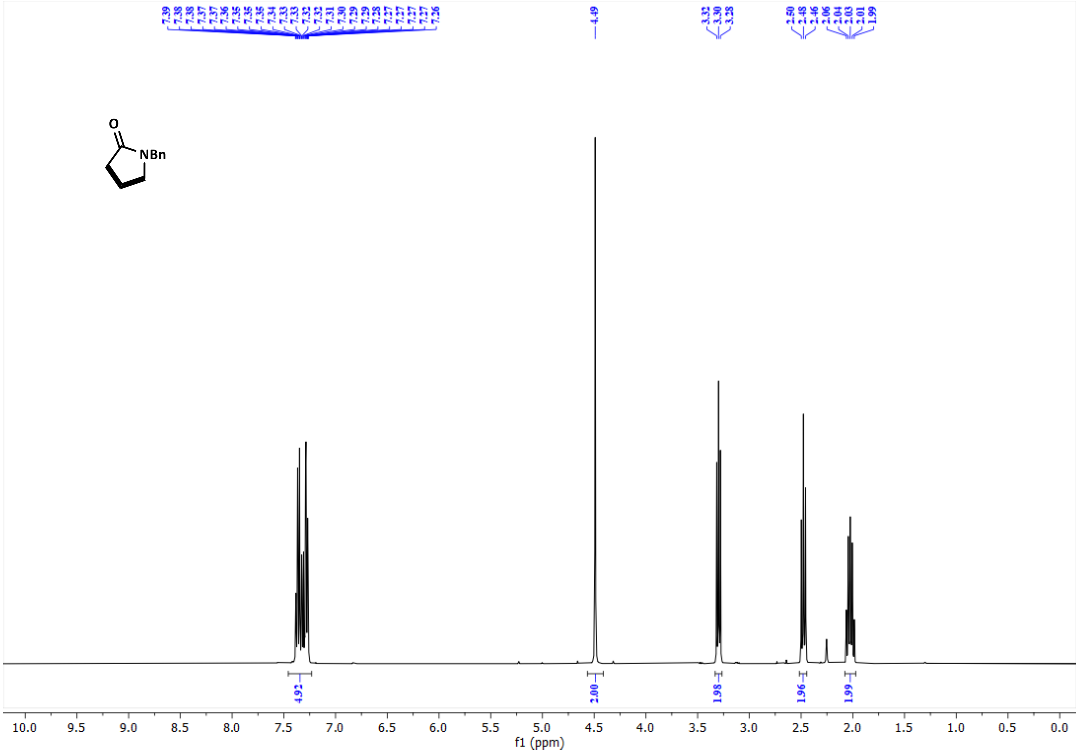

*Compound **9**. ^{13}C NMR (100 MHz, CHLOROFORM-D).*

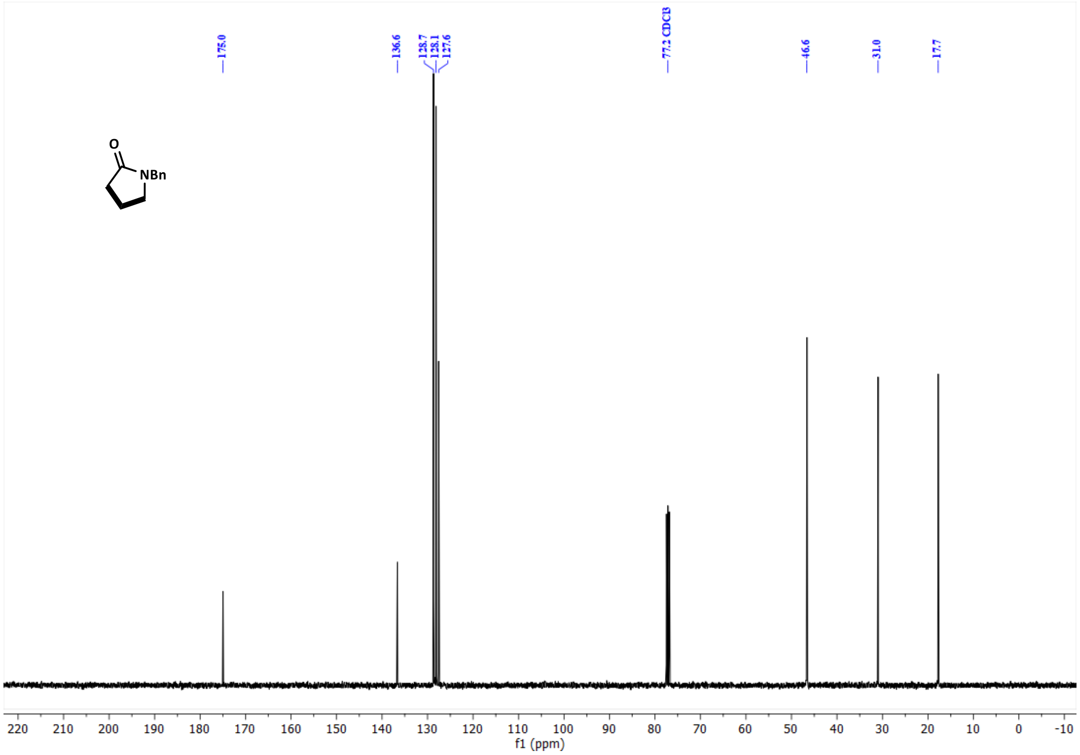

*Compound **10**. ^{1}H NMR (400 MHz, CHLOROFORM-D).*

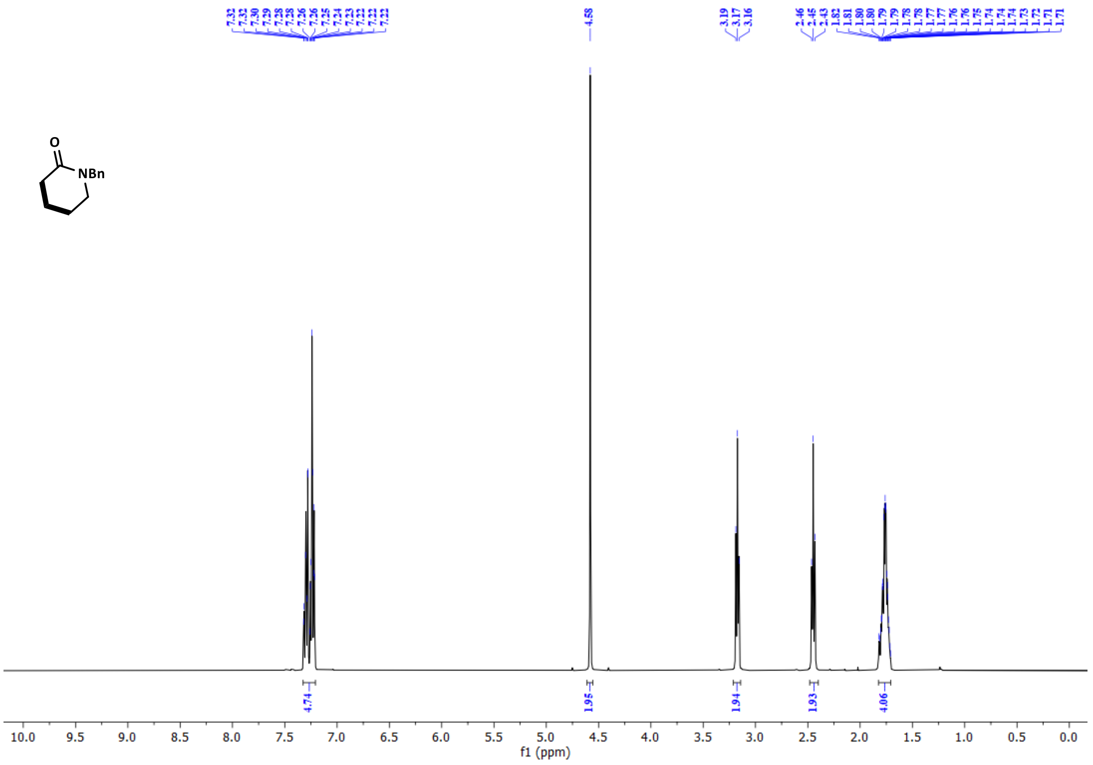

*Compound **10**. ^{13}C NMR (100 MHz, CHLOROFORM-D).*

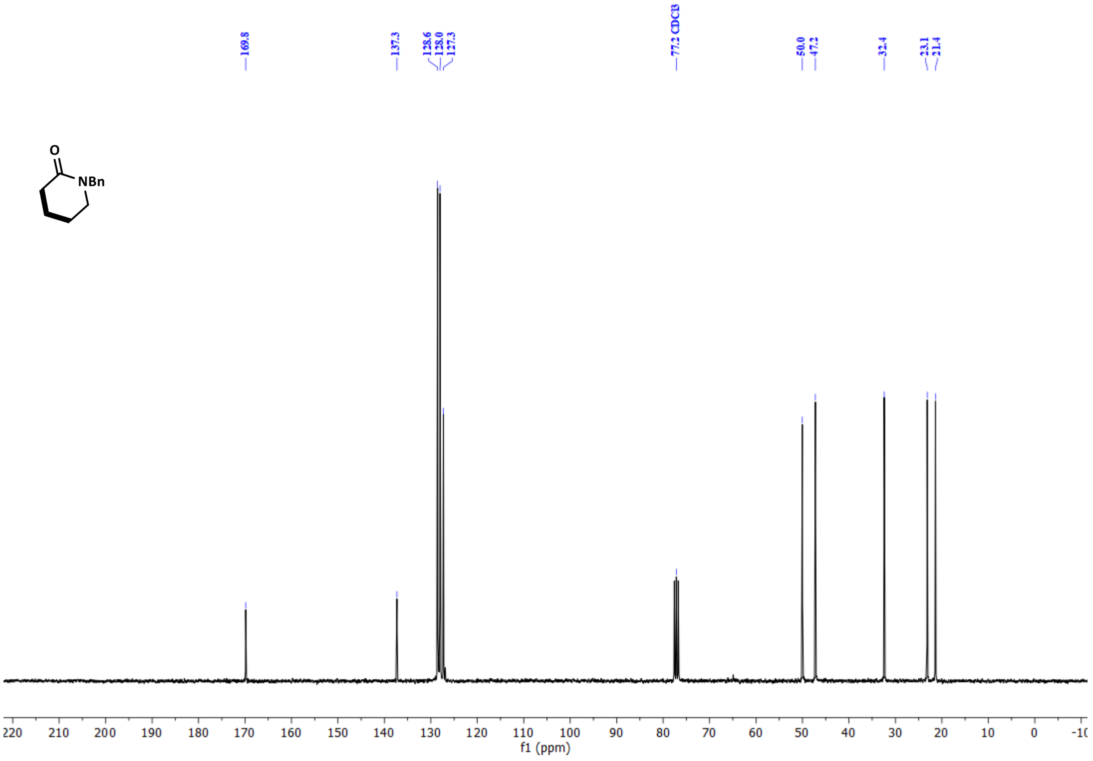

*Compound **11**. ^{1}H NMR (400 MHz, CHLOROFORM-D).*

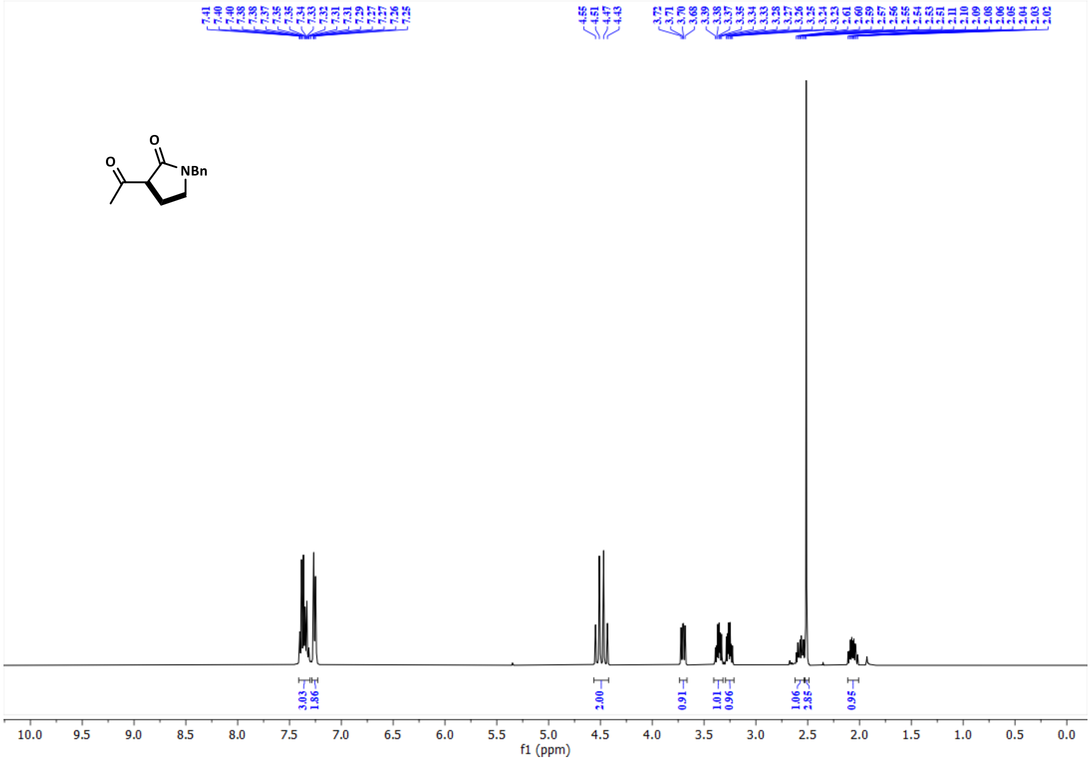

*Compound **11**. ^{13}C NMR (100 MHz, CHLOROFORM-D).*

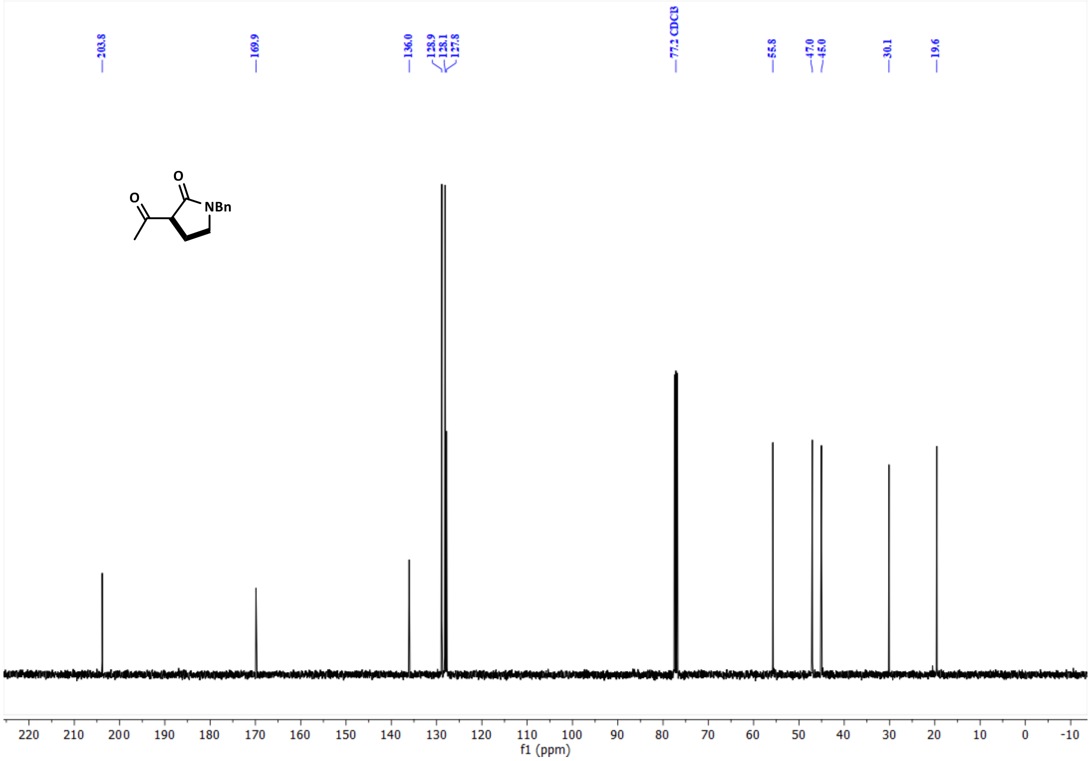

*Compound **12**. ^{1}H NMR (400 MHz, CHLOROFORM-D).*

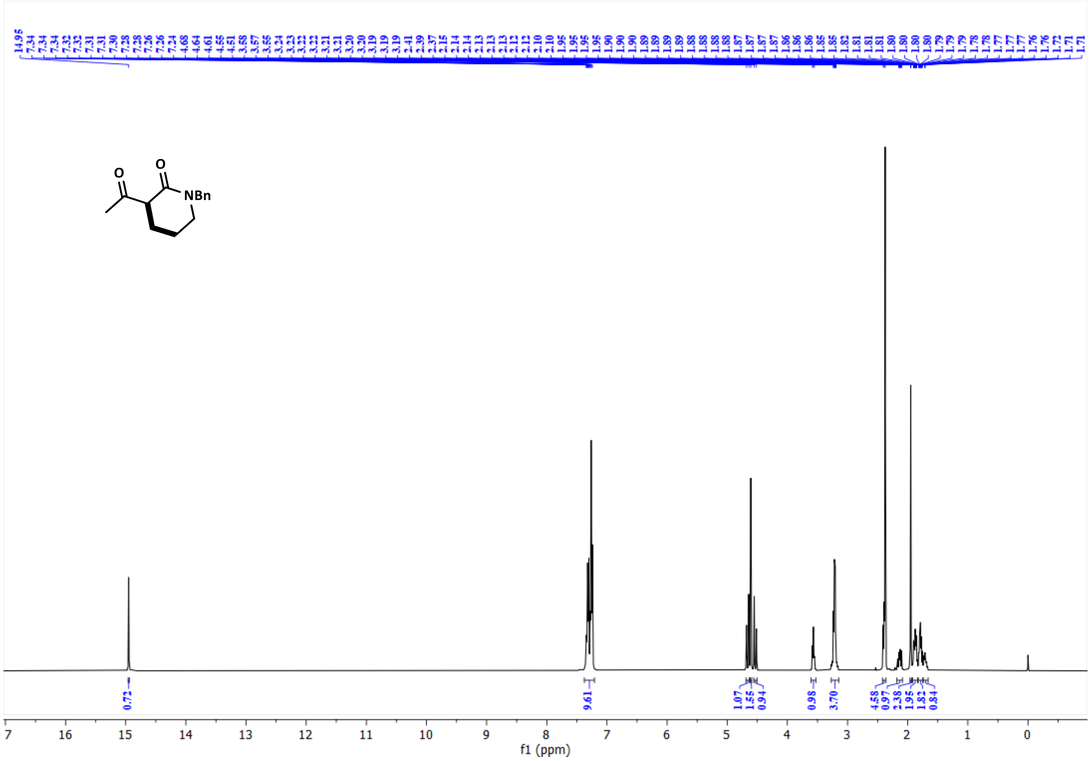

*Compound **12**. ^{13}C NMR (100 MHz, CHLOROFORM-D).*

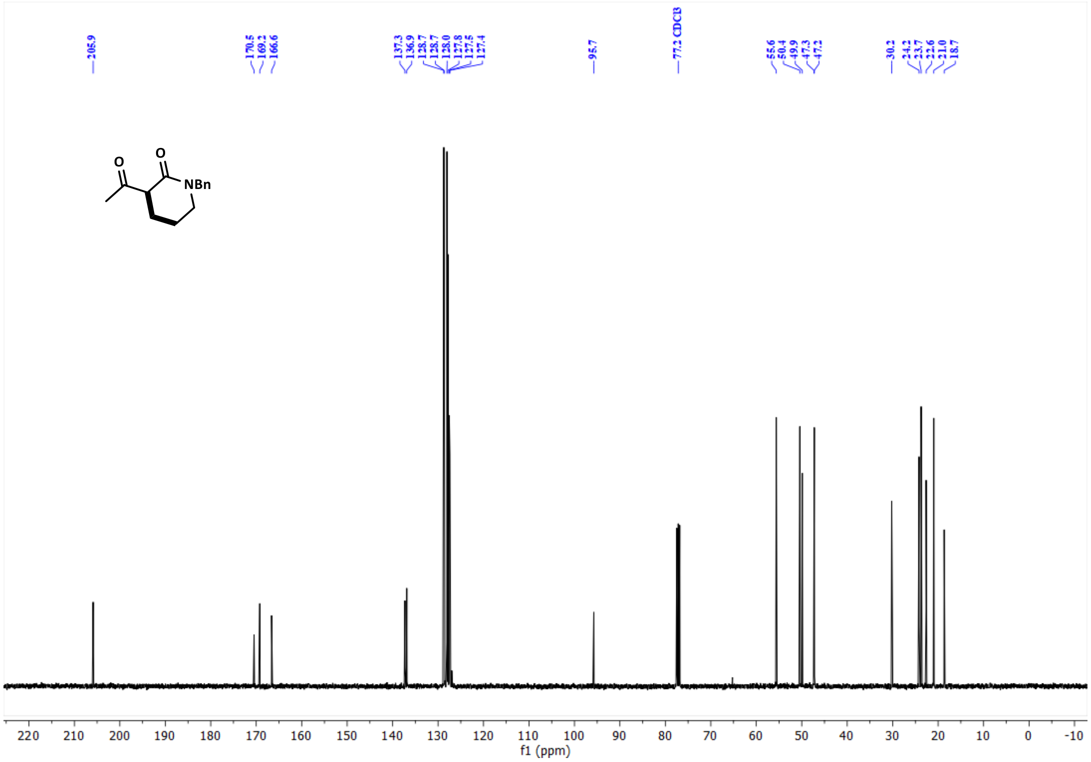

*Compound **13**. ¹H NMR (400 MHz, CHLOROFORM-D).*

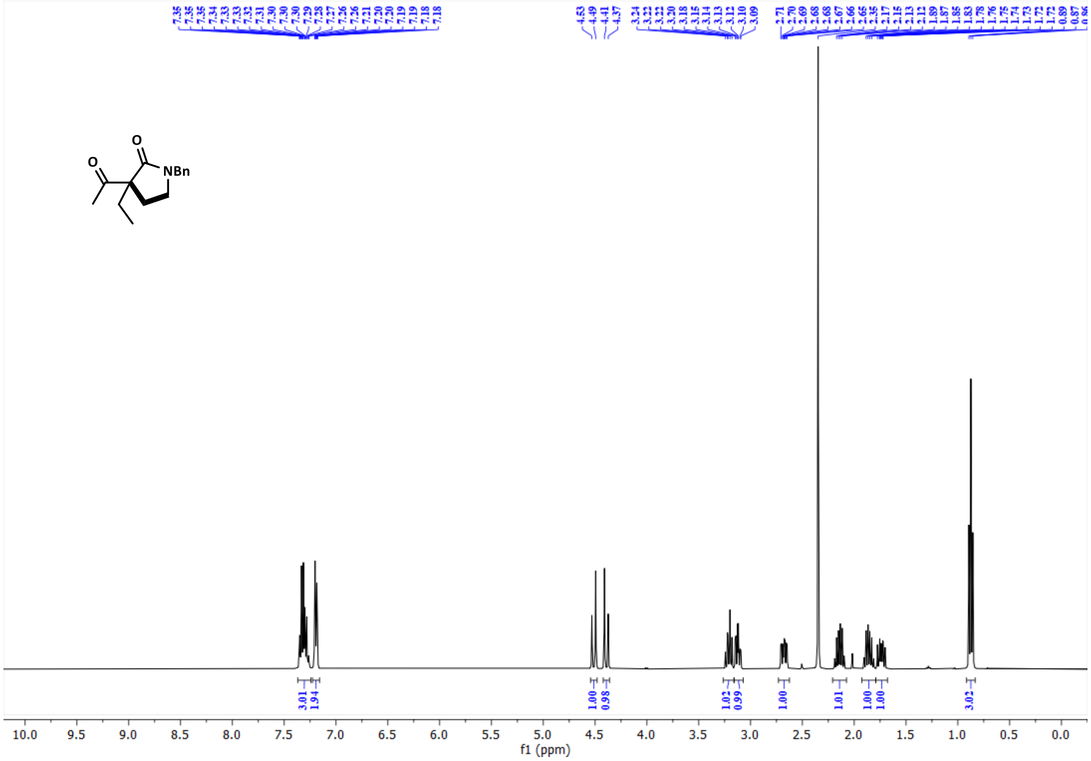

*Compound **13**. ¹³C NMR (100 MHz, CHLOROFORM-D).*

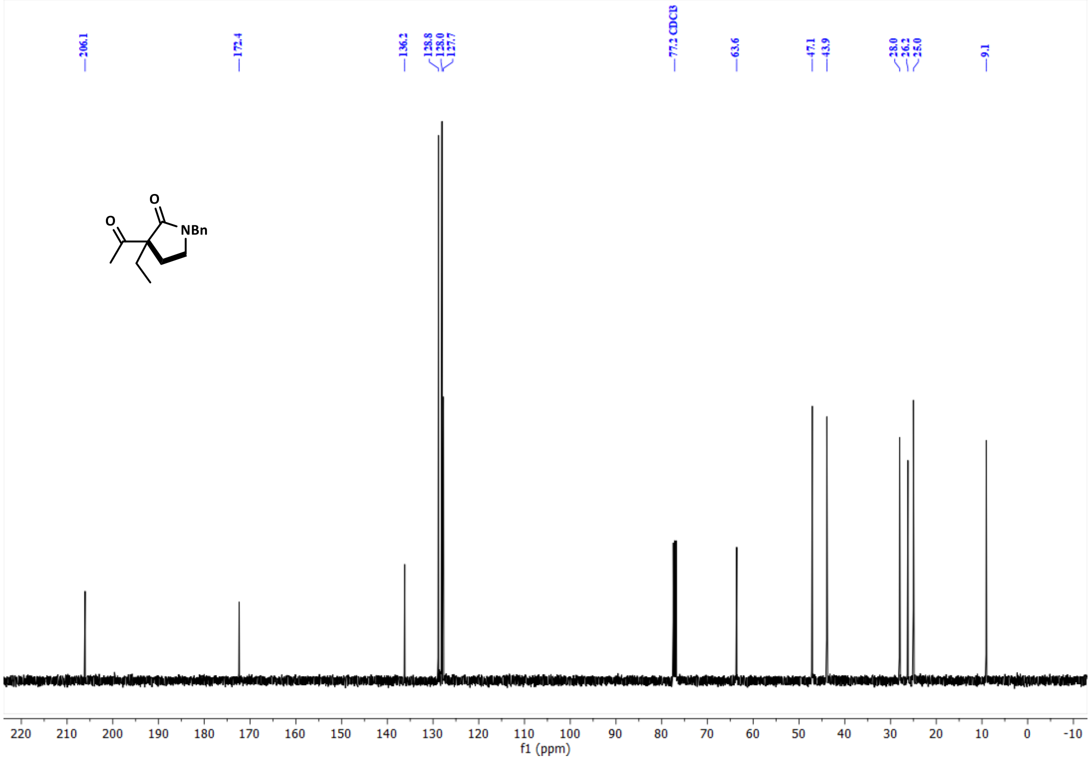

*Compound **14**. ^{1}H NMR (400 MHz, CHLOROFORM-D).*

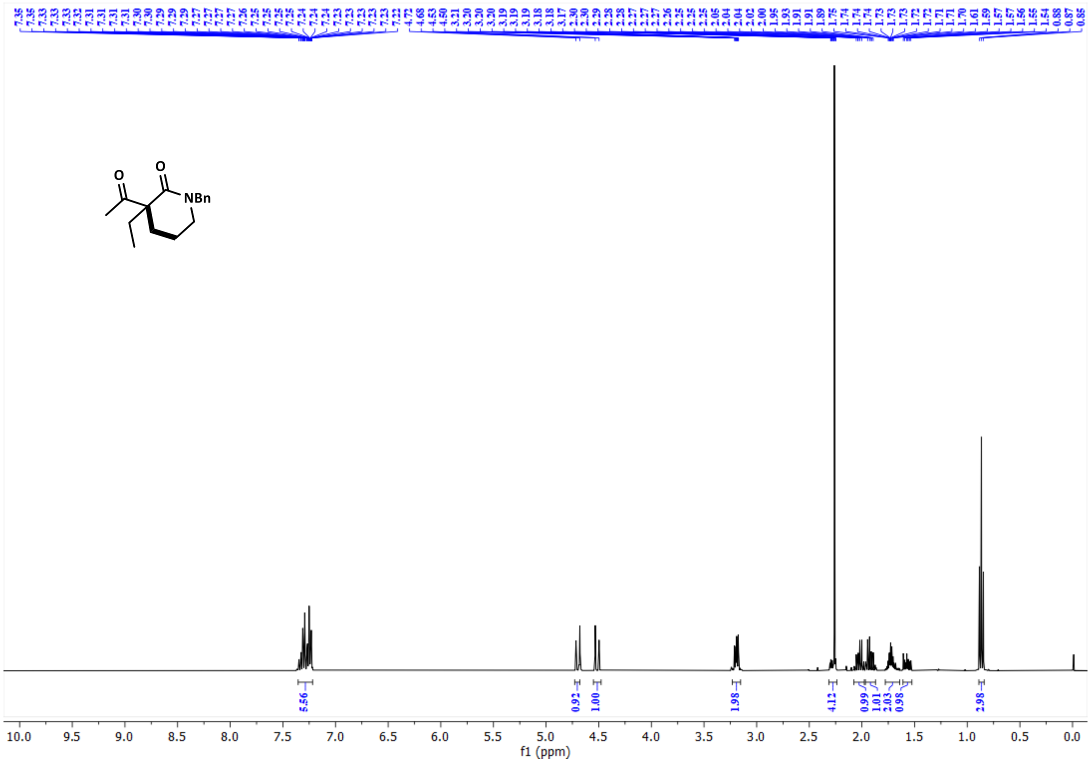

*Compound **14**. ^{13}C NMR (100 MHz, CHLOROFORM-D).*

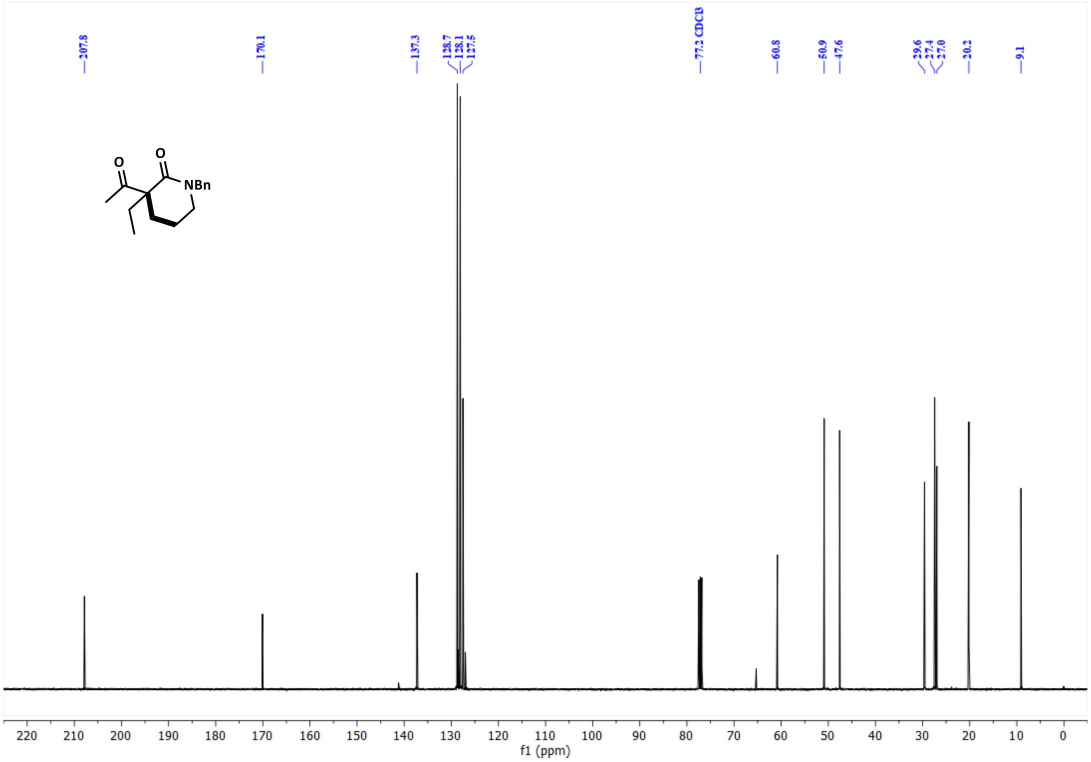

*Compound **15**. ^{1}H NMR (400 MHz, CHLOROFORM-D).*

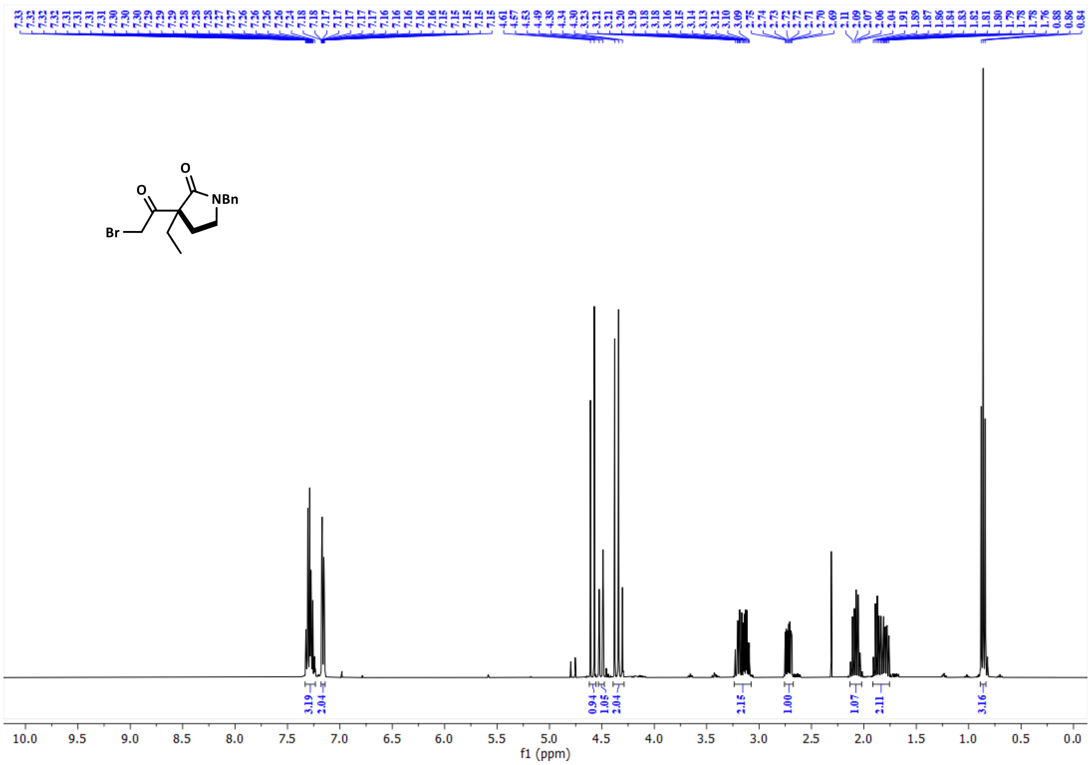

*Compound **15**. ^{13}C NMR (100 MHz, CHLOROFORM-D).*

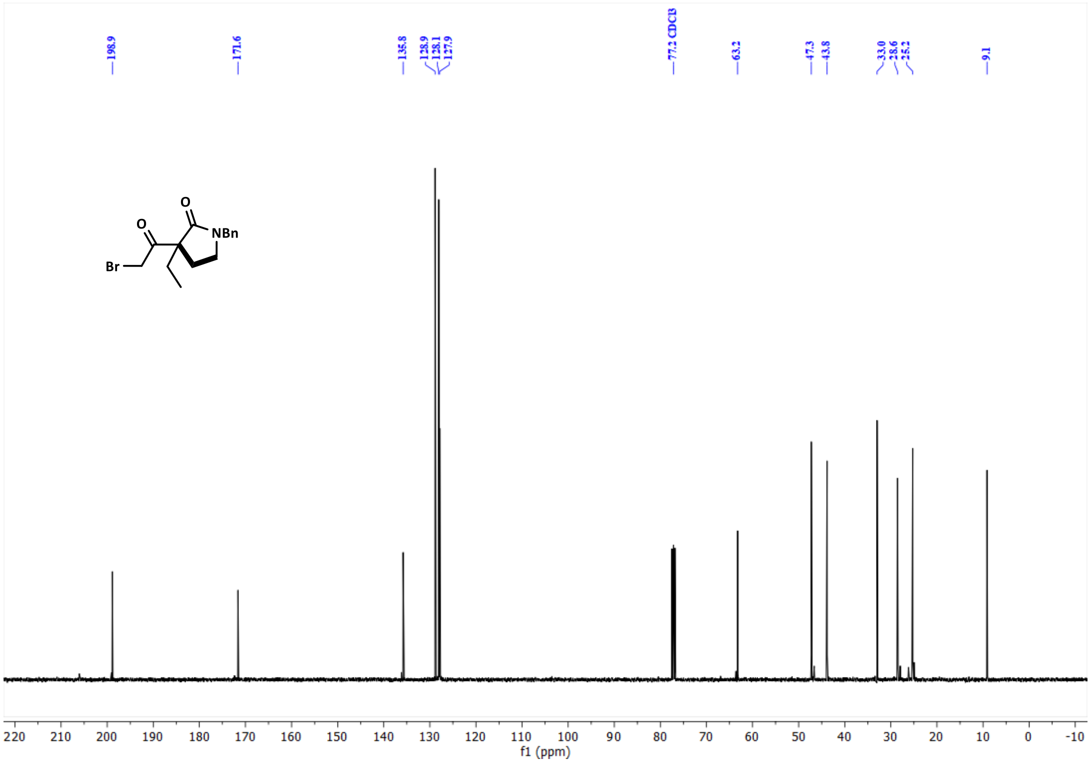

*Compound **16**. ^{1}H NMR (400 MHz, CHLOROFORM-D).*

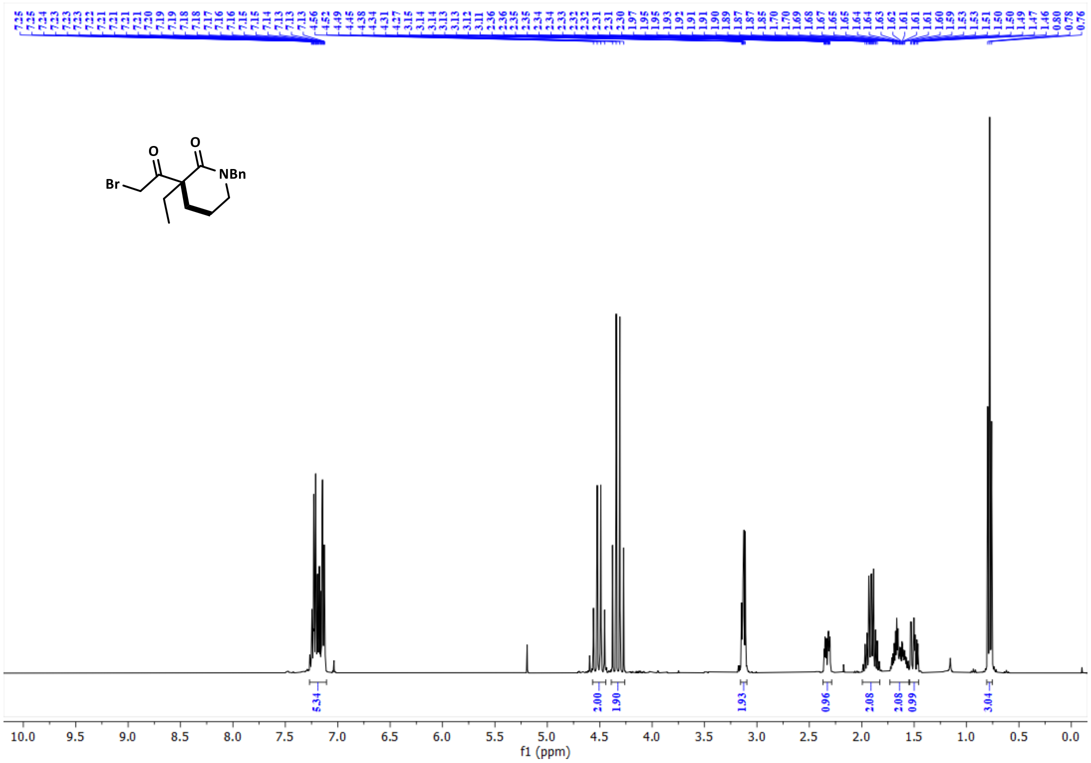

*Compound **16**. ^{13}C NMR (100 MHz, CHLOROFORM-D).*

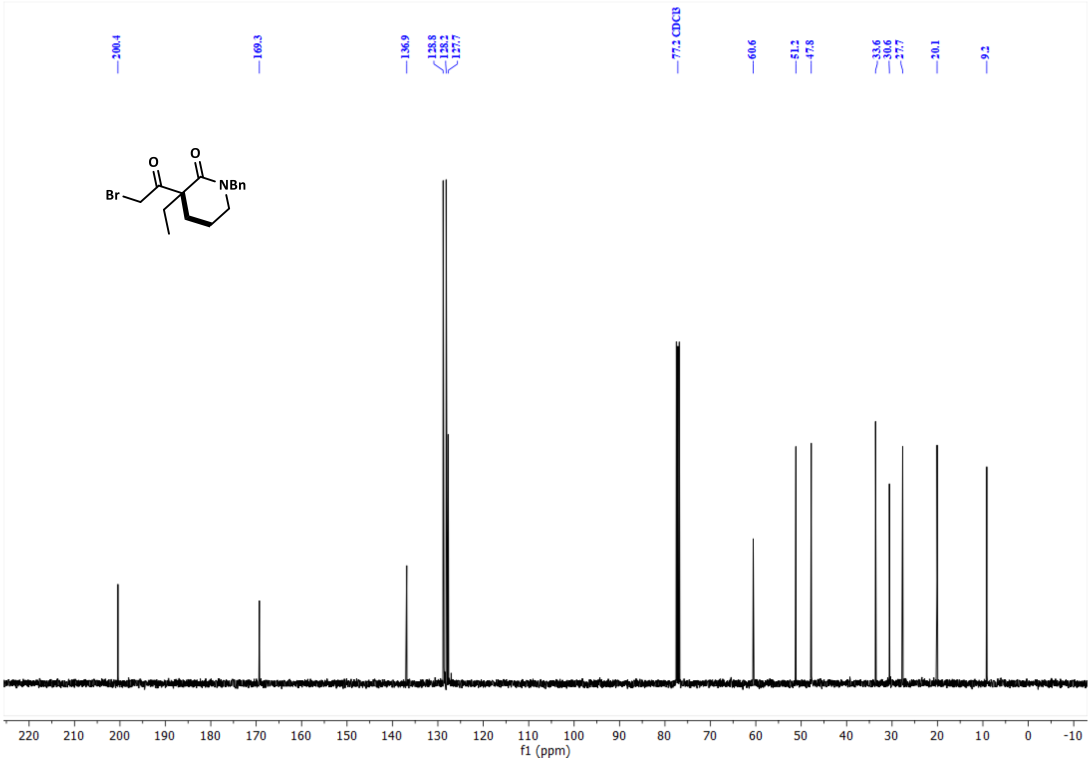

Compound ***17***. ^{1}H NMR (400 MHz, CHLOROFORM-D).

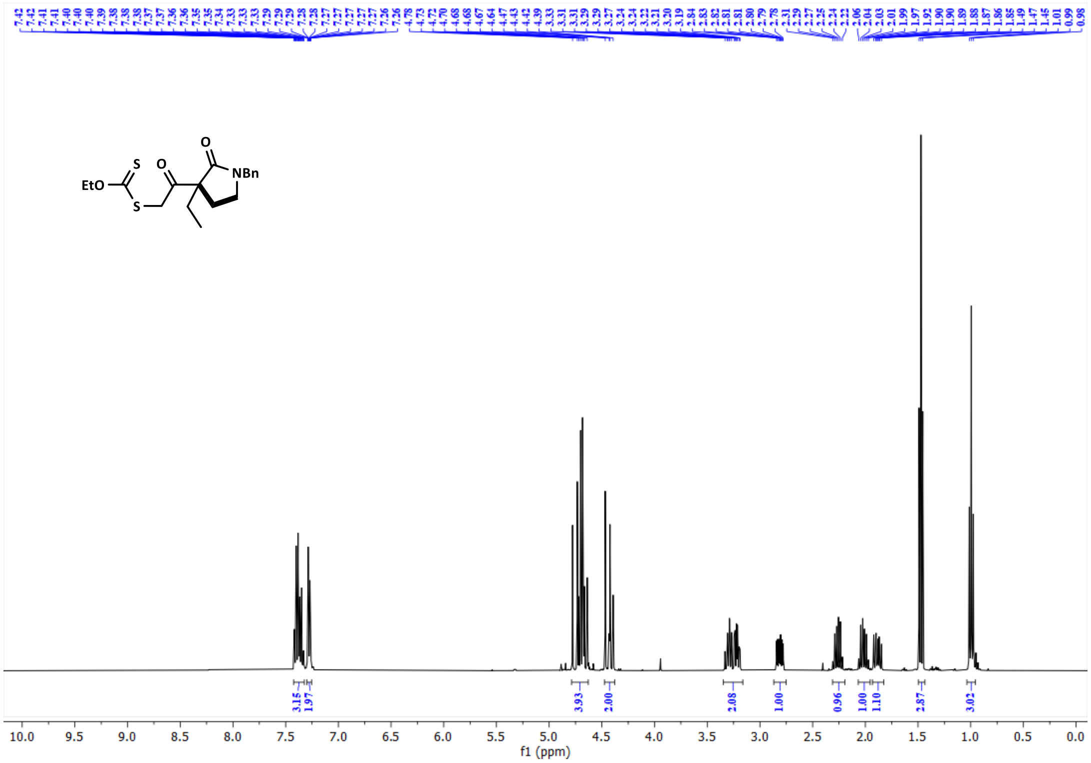

Compound ***17***. ^{13}C NMR (100 MHz, CHLOROFORM-D).

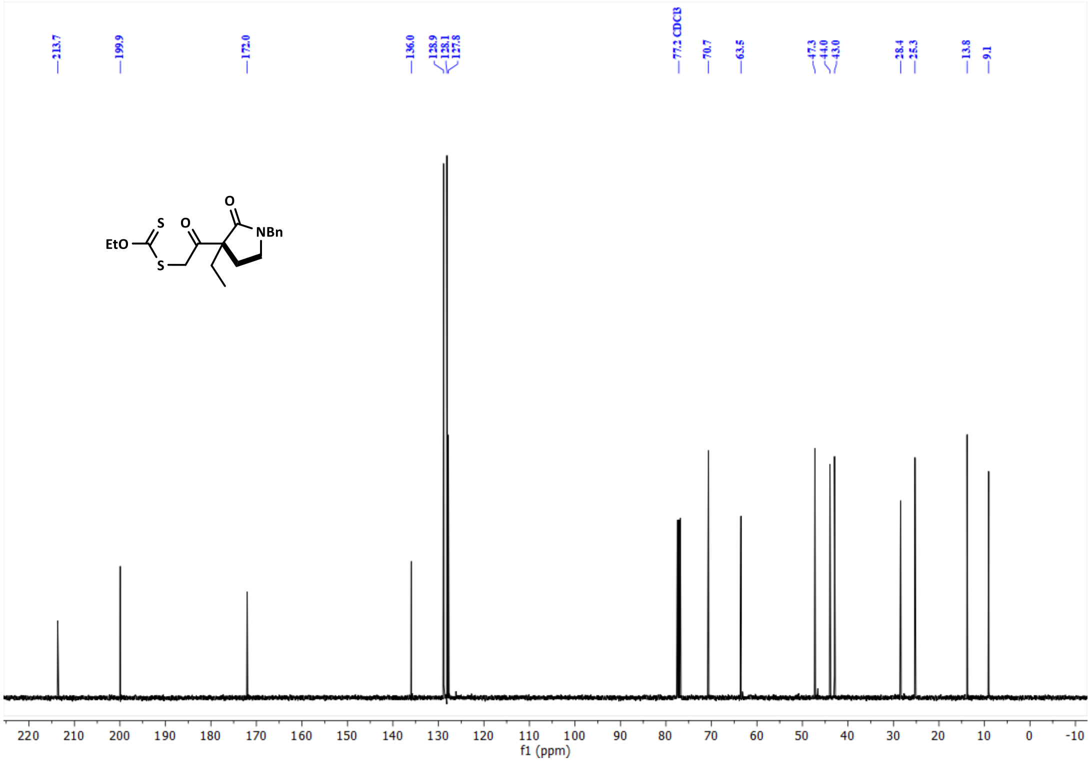

Compound **18**. ^{1}H NMR (400 MHz, CHLOROFORM-D).

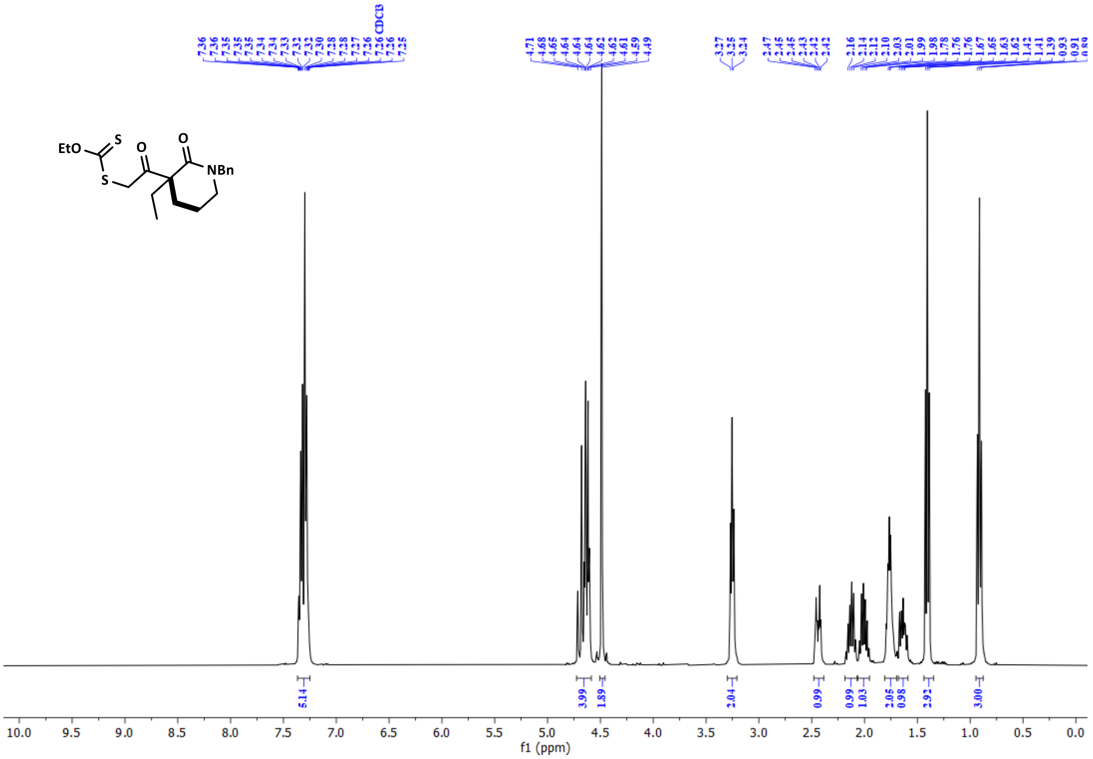

Compound **18**. ^{13}C NMR (100 MHz, CHLOROFORM-D).

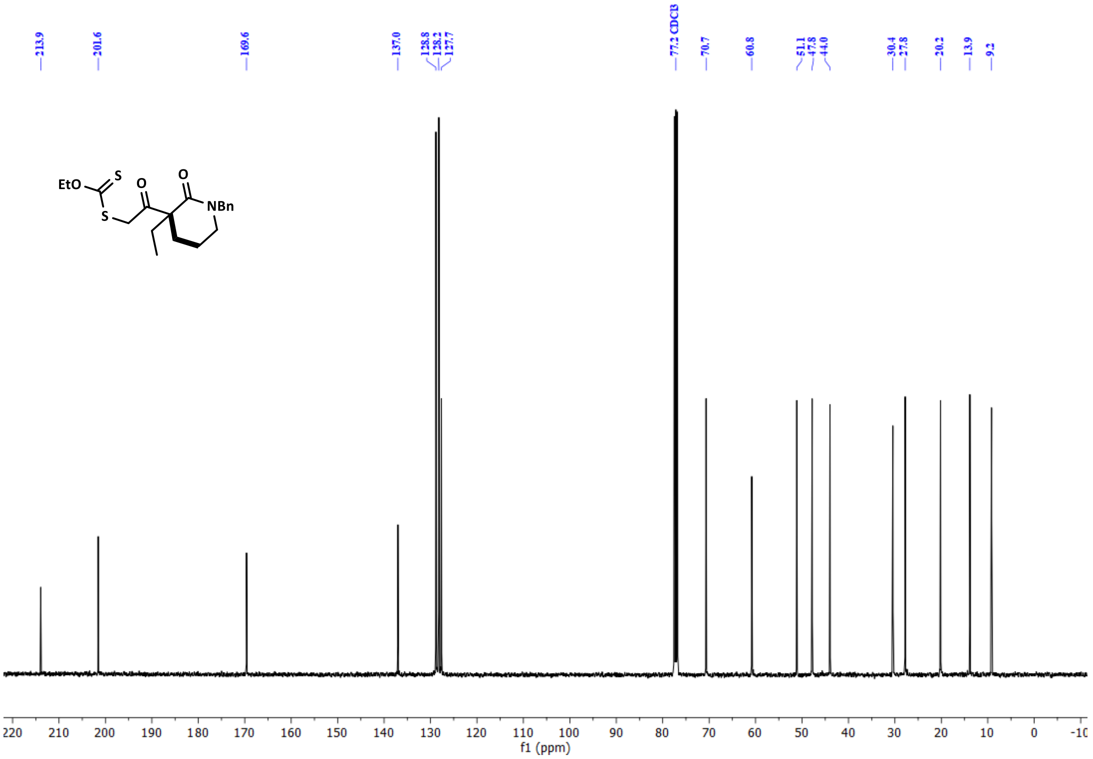

*Compound **19a**. ^{1}H NMR (400 MHz, CHLOROFORM-D).*

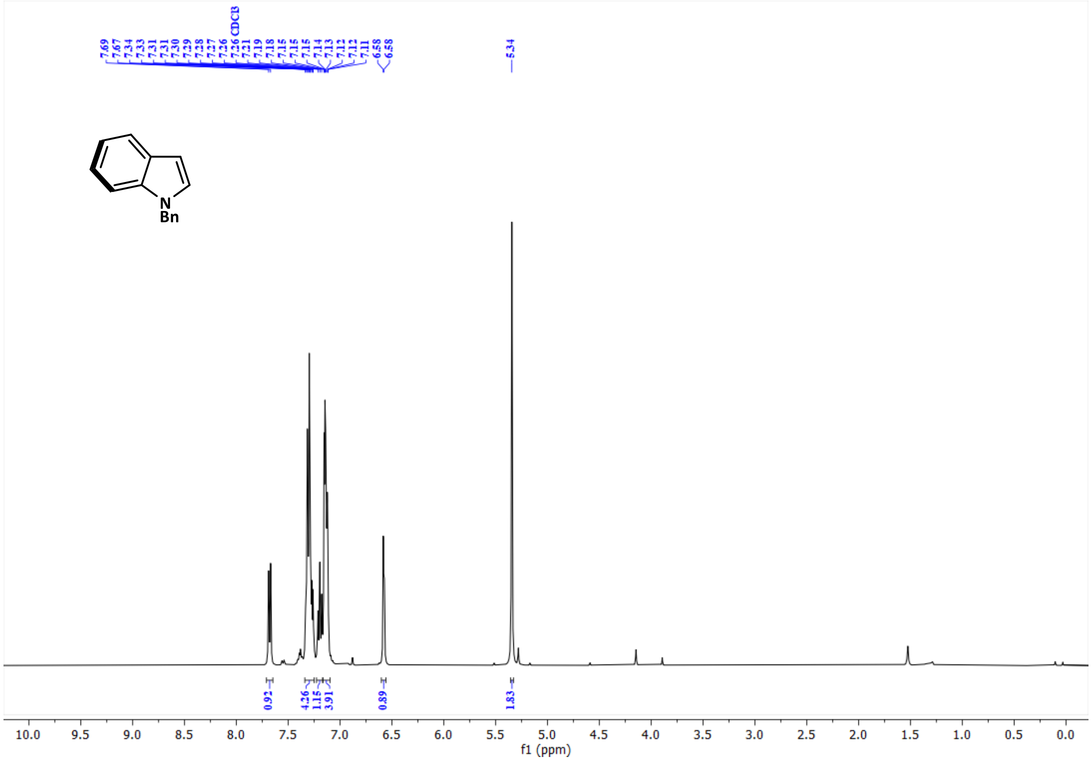

*Compound **19a**. ^{13}C NMR (100 MHz, CHLOROFORM-D).*

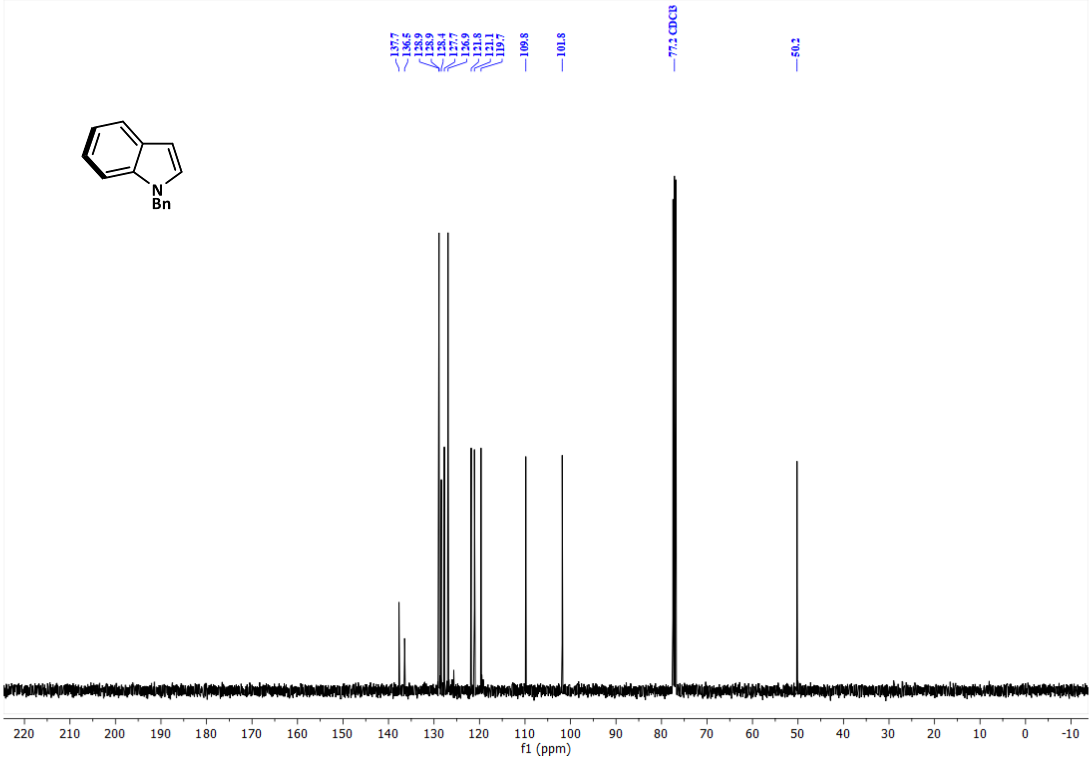

Compound **Tryptophol-TBS**. ^{1}H NMR (400 MHz, CHLOROFORM-D).

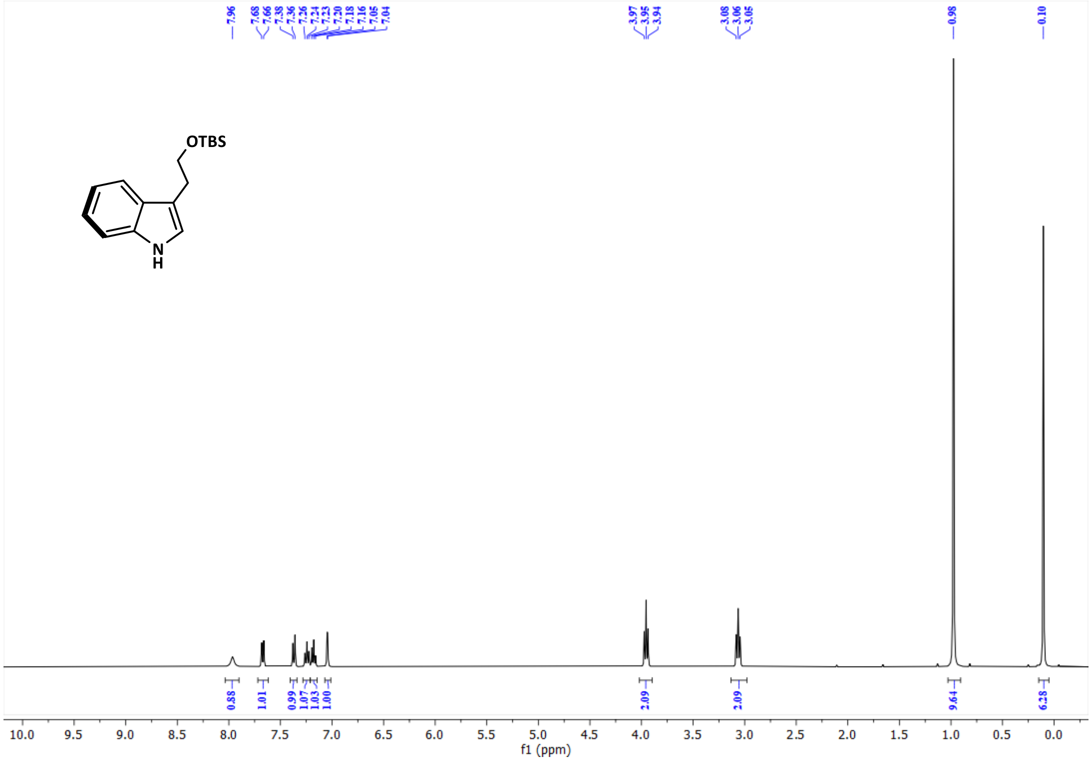

Compound **Tryptophol-TBS**. ^{13}C NMR (100 MHz, CHLOROFORM-D).

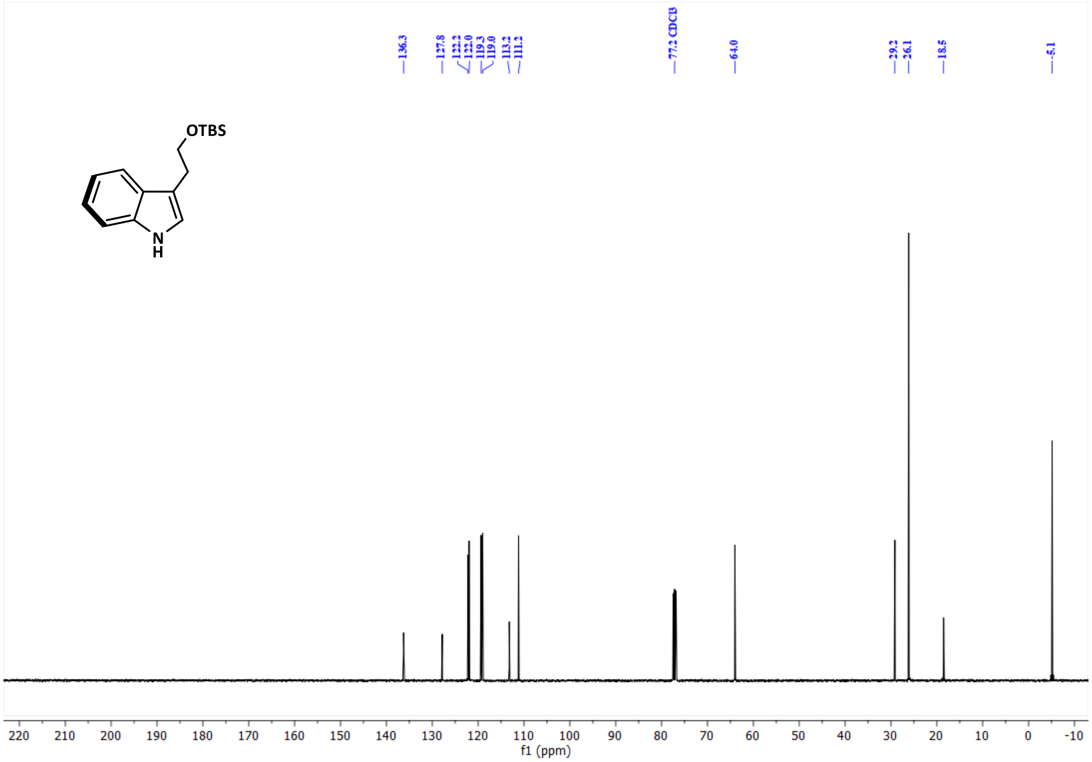

*Compound **19b**. ¹H NMR (400 MHz, CHLOROFORM-D).*

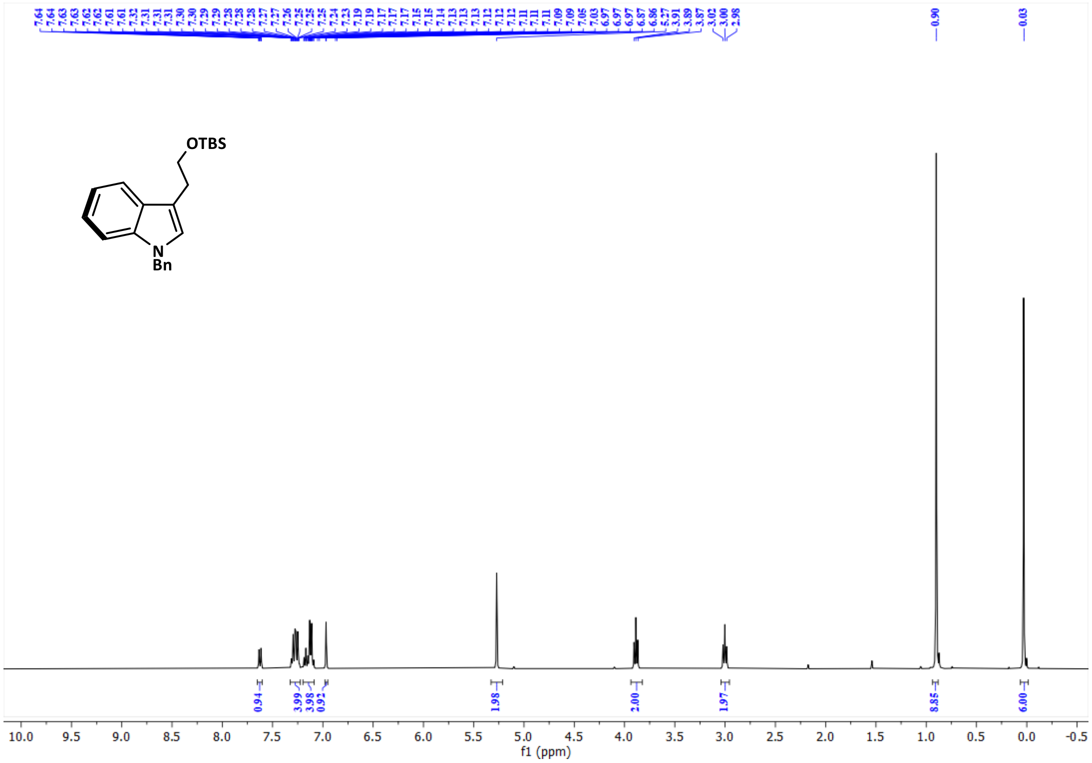

*Compound **19b**. ¹³C NMR (100 MHz, CHLOROFORM-D)*

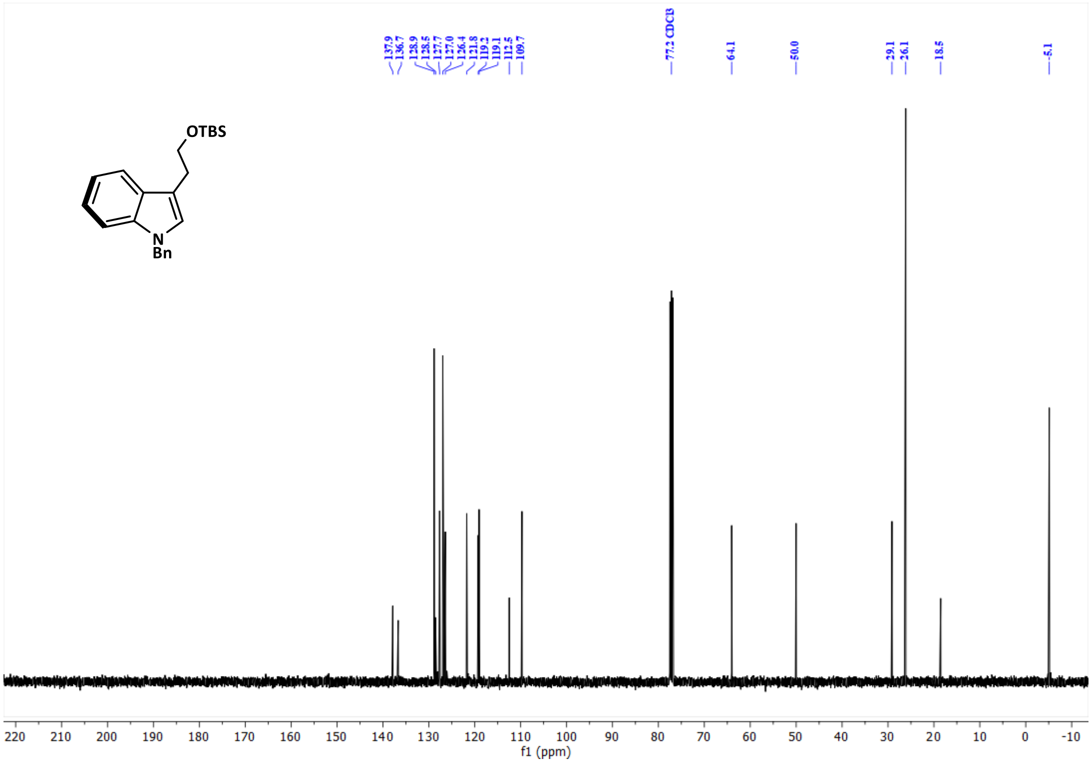

*Compound **Tryptamine-Boc**. ^{1}H NMR (400 MHz, CHLOROFORM-D).*

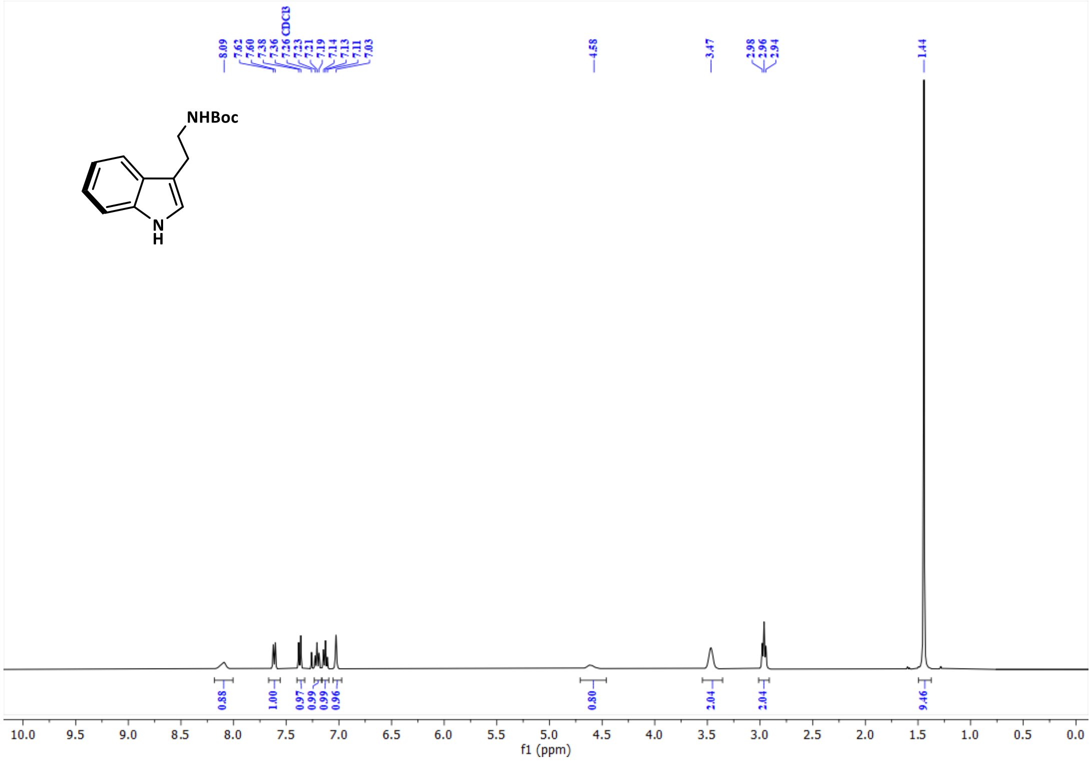

*Compound **Tryptamine-Boc**. ^{13}C NMR (100 MHz, CHLOROFORM-D).*

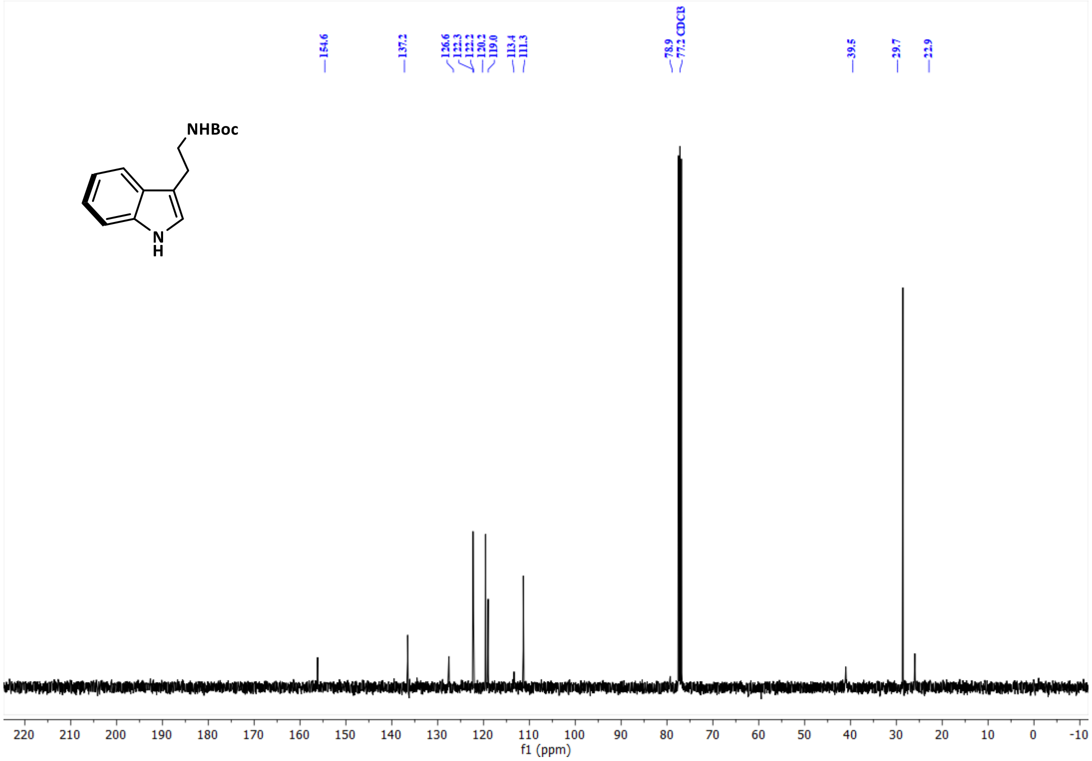

*Compound **19c**. ^{1}H NMR (400 MHz, CHLOROFORM-D).*

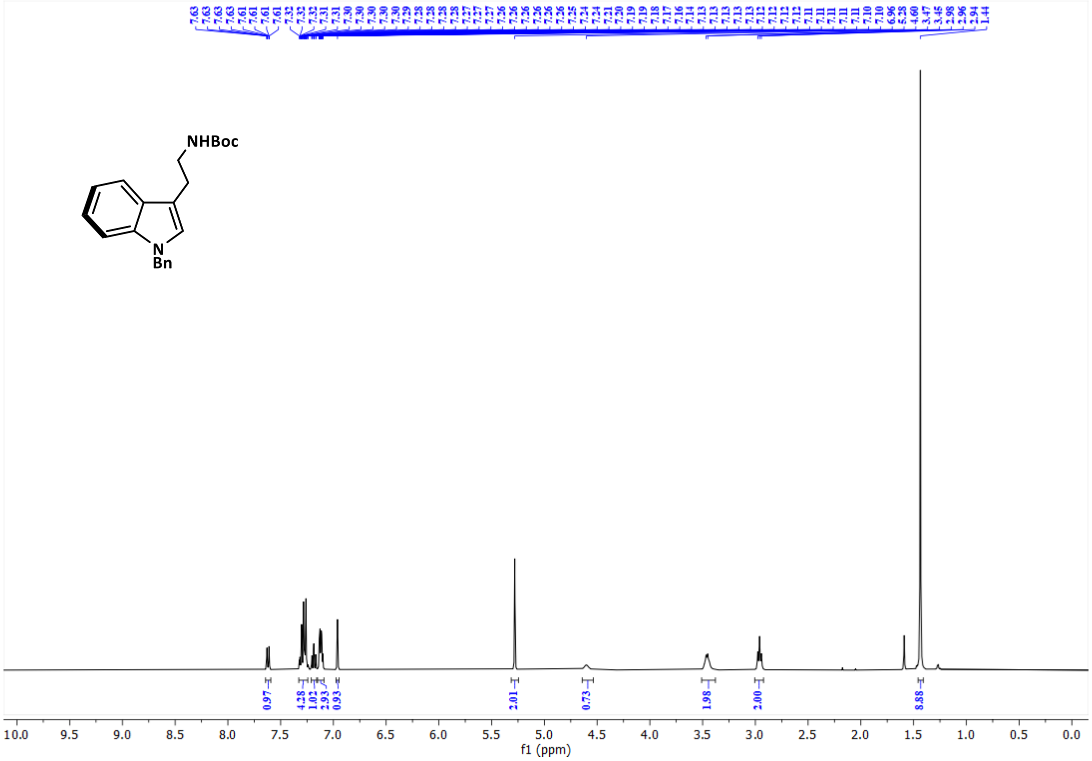

*Compound **19c**. ^{13}C NMR (100 MHz, CHLOROFORM-D).*

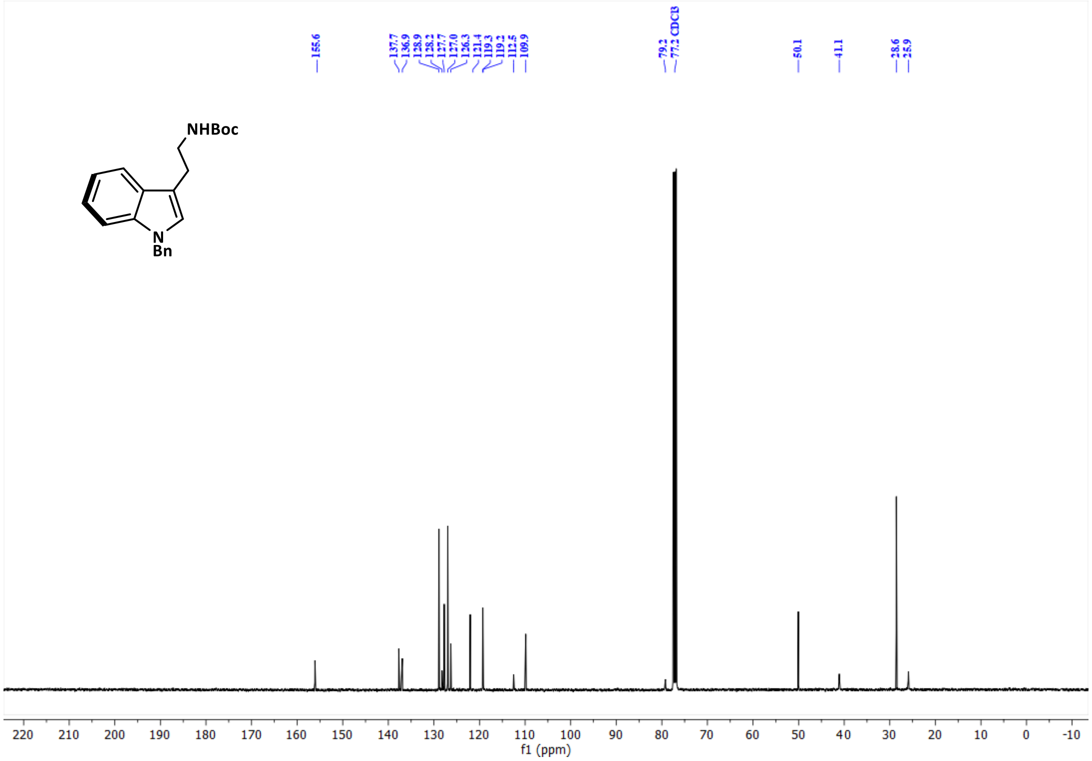

*Compound **19d**. ^{1}H NMR (400 MHz, CHLOROFORM-D).*

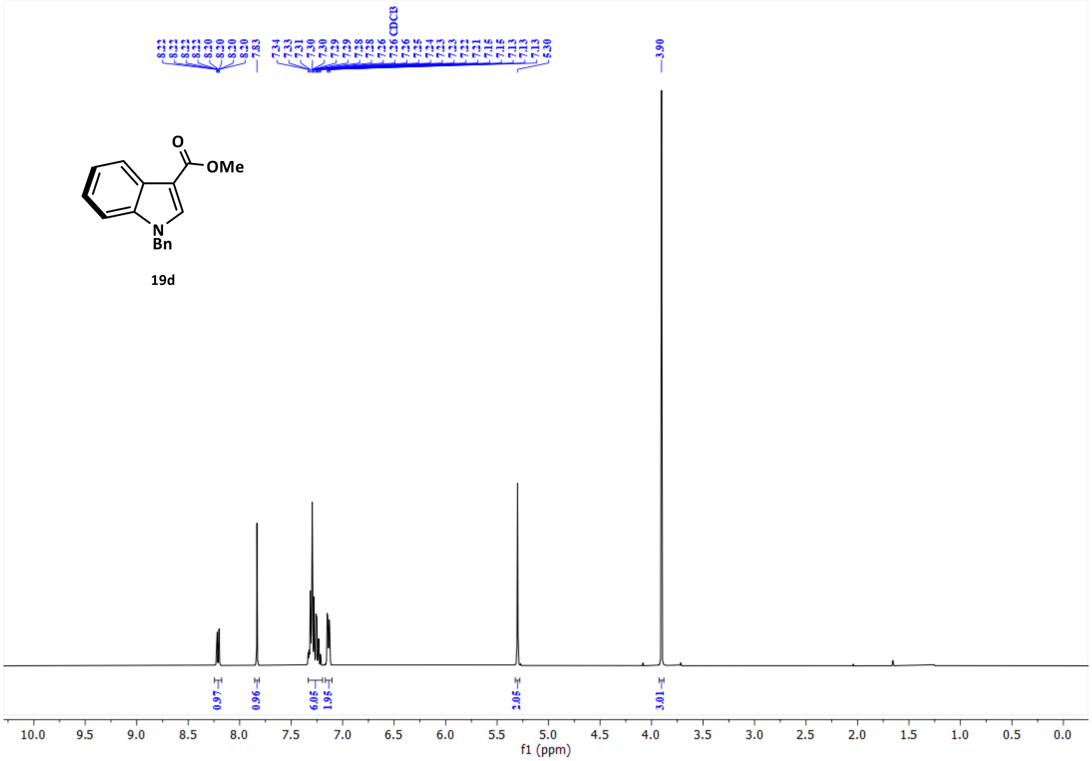

*Compound **19d**. ^{13}C NMR (100 MHz, CHLOROFORM-D).*

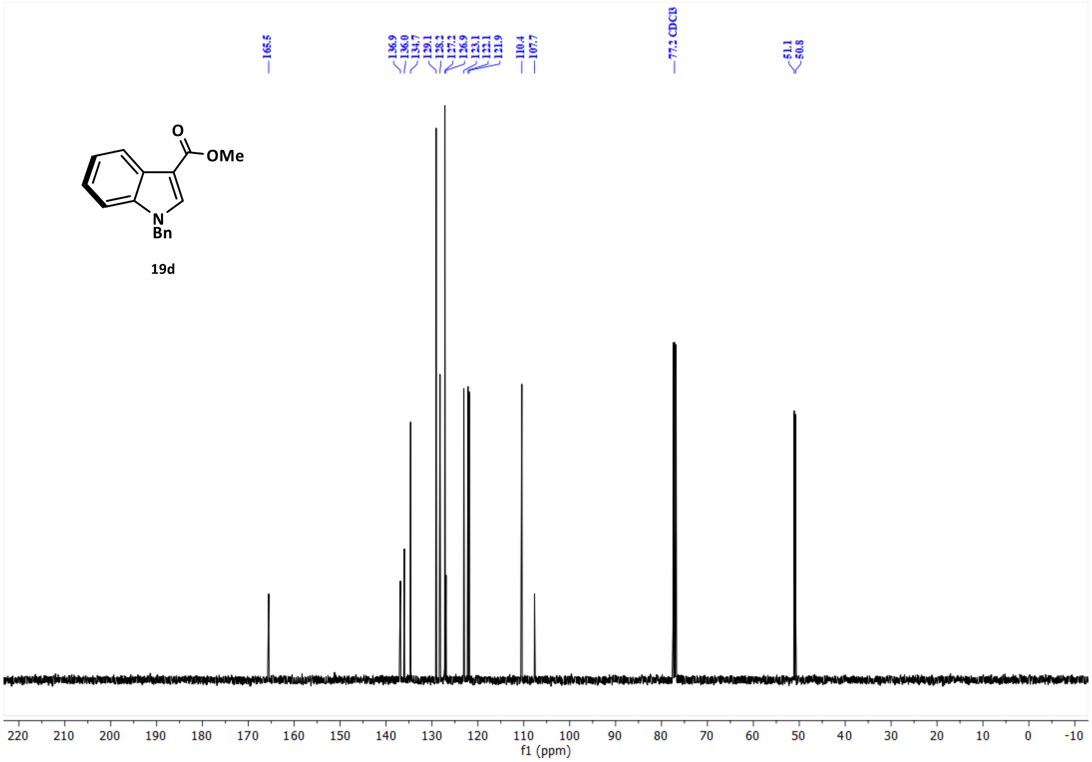

*Compound **19e**. ^{1}H NMR (400 MHz, CHLOROFORM-D).*

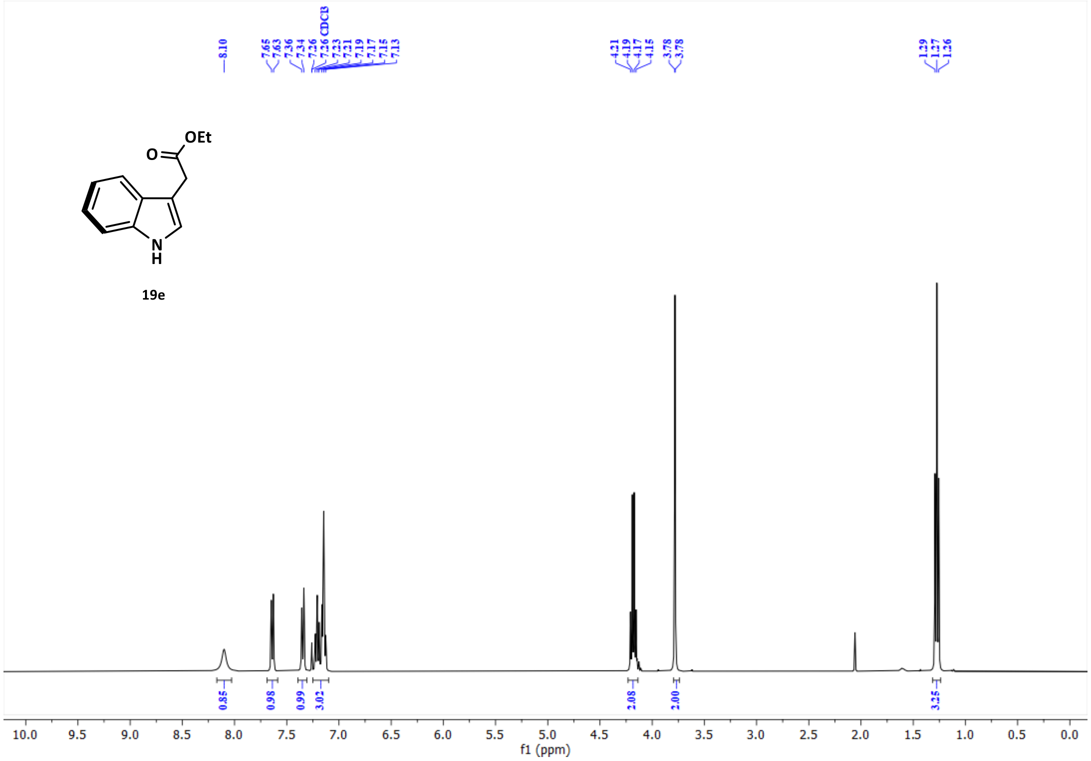

*Compound **19e**. ^{13}C NMR (100 MHz, CHLOROFORM-D).*

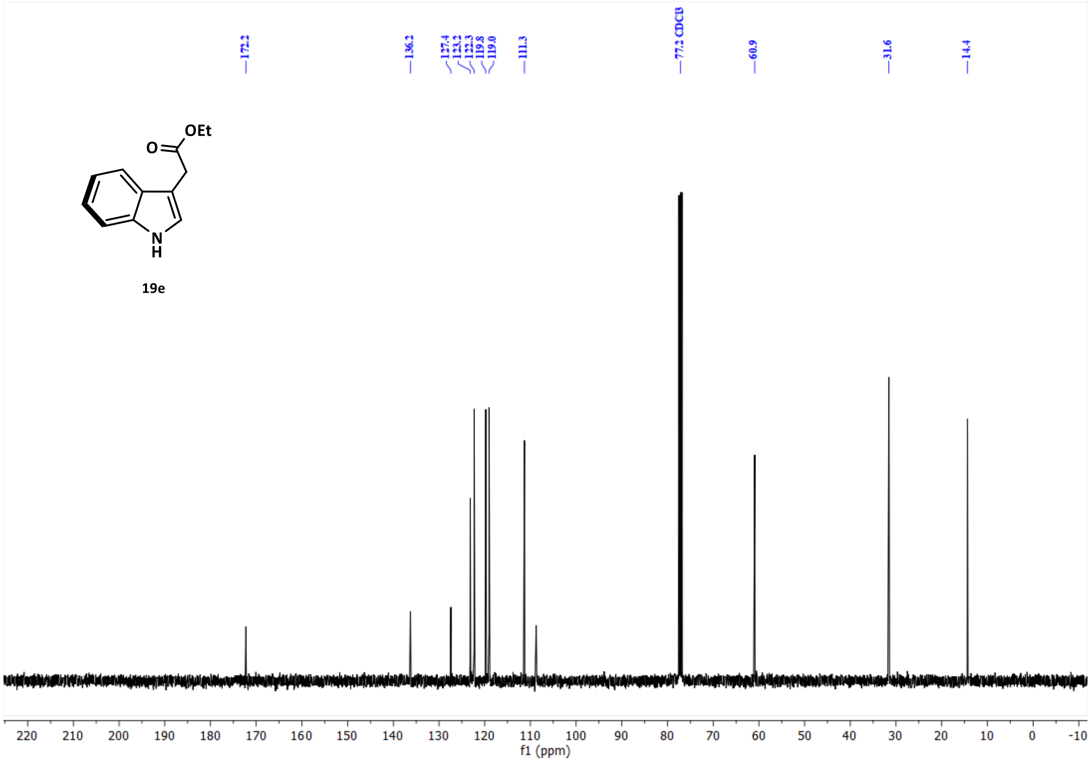

*Compound **20**. ^{1}H NMR (400 MHz, ACETONE-D_6).*

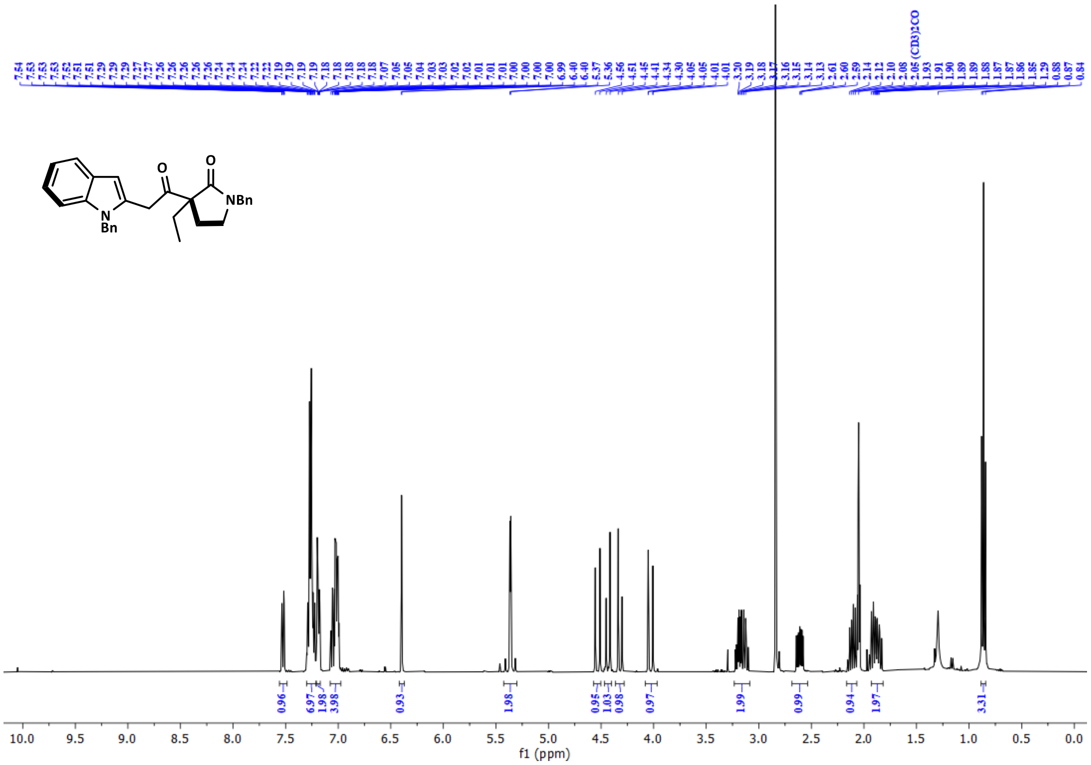

*Compound **20**. ^{13}C NMR (100 MHz, ACETONE-D_6).*

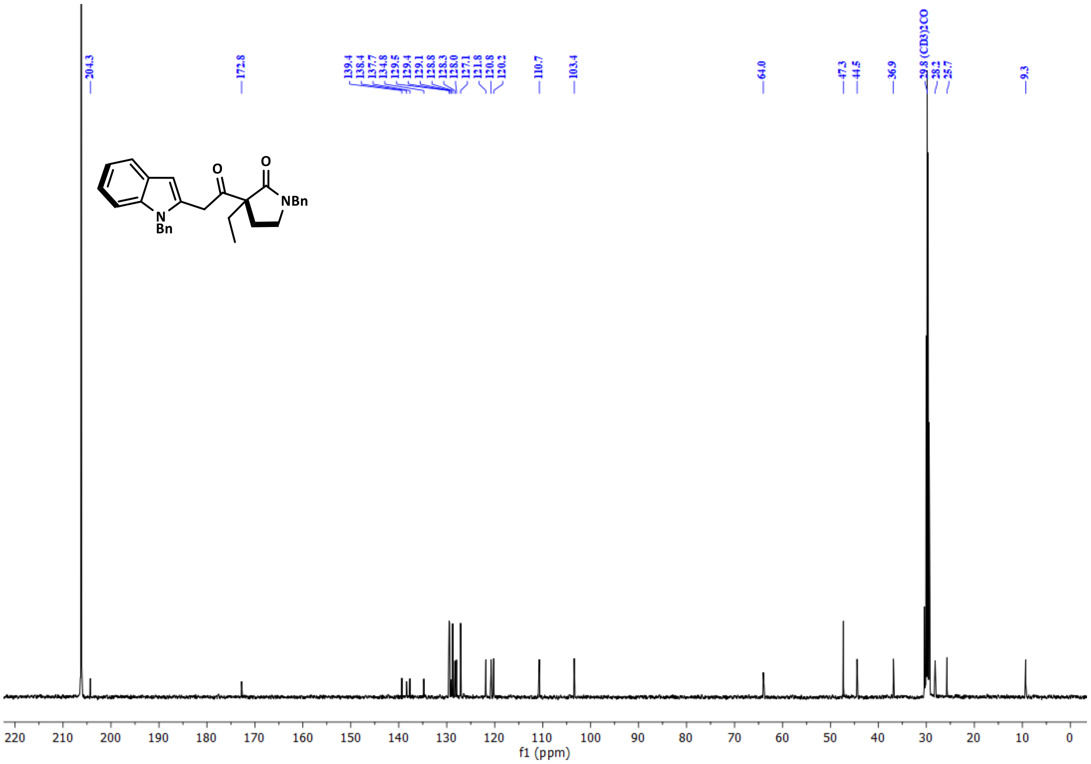

*Compound **21**. ^{1}H NMR (400 MHz, CHLOROFORM-D).*

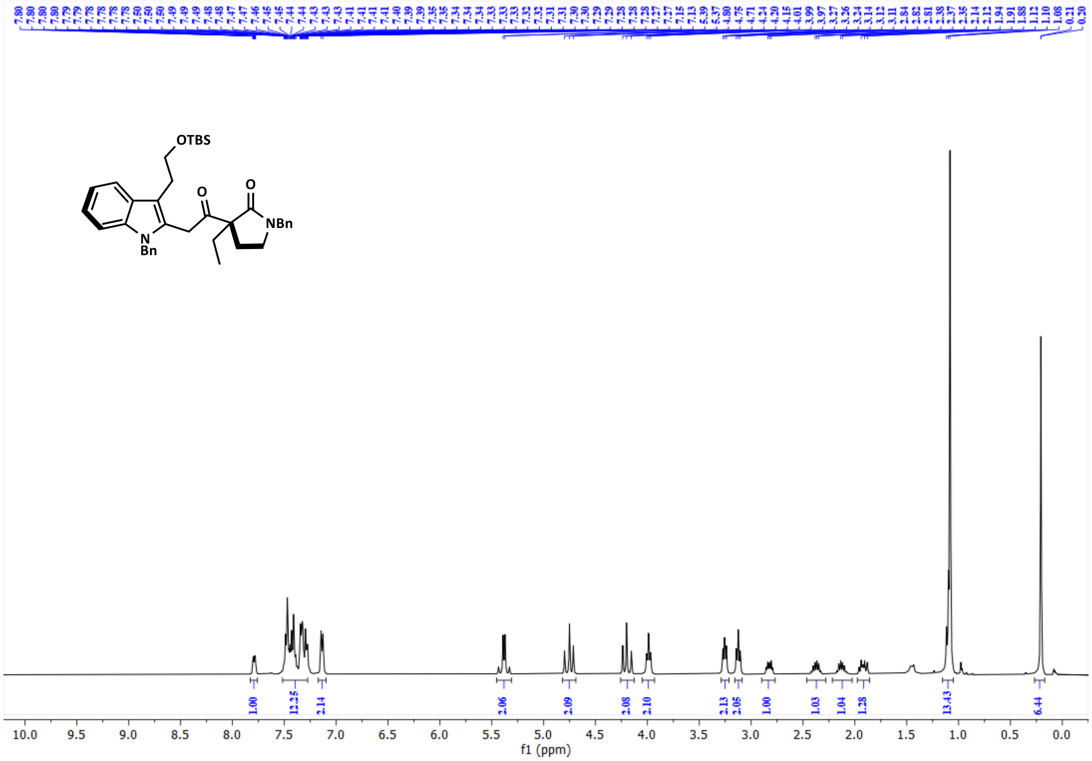

*Compound **21**. ^{13}C NMR (100 MHz, CHLOROFORM-D).*

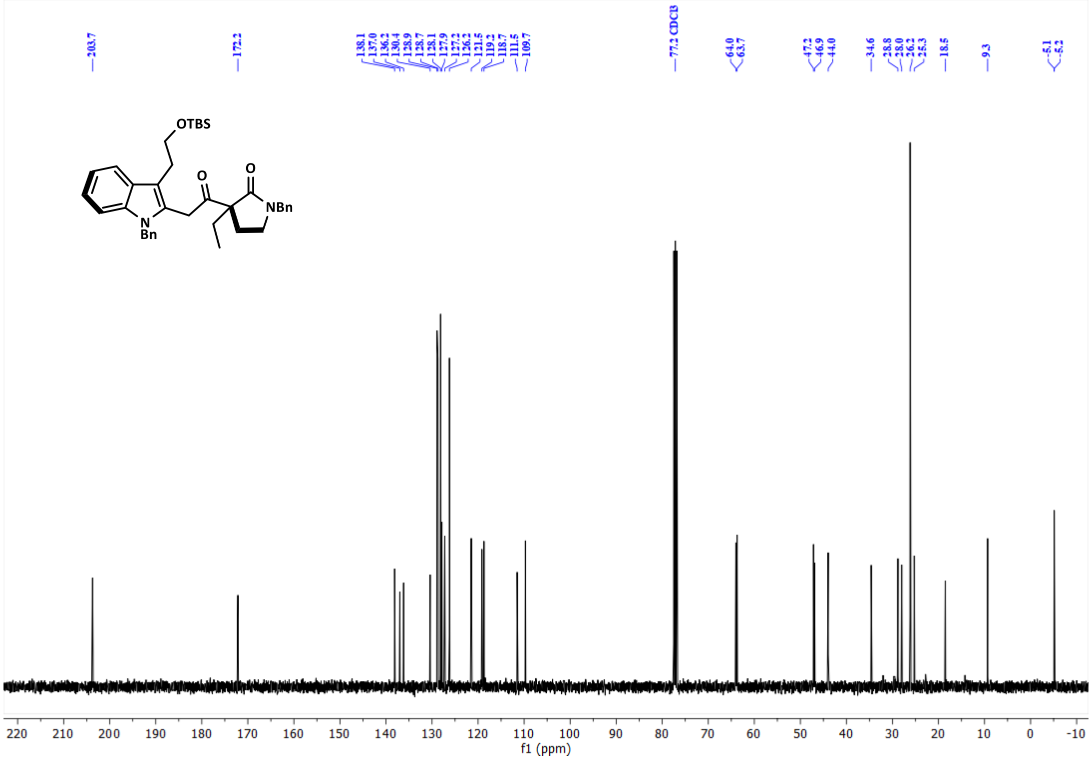

*Compound **22**. ^{1}H NMR (400 MHz, CHLOROFORM-D).*

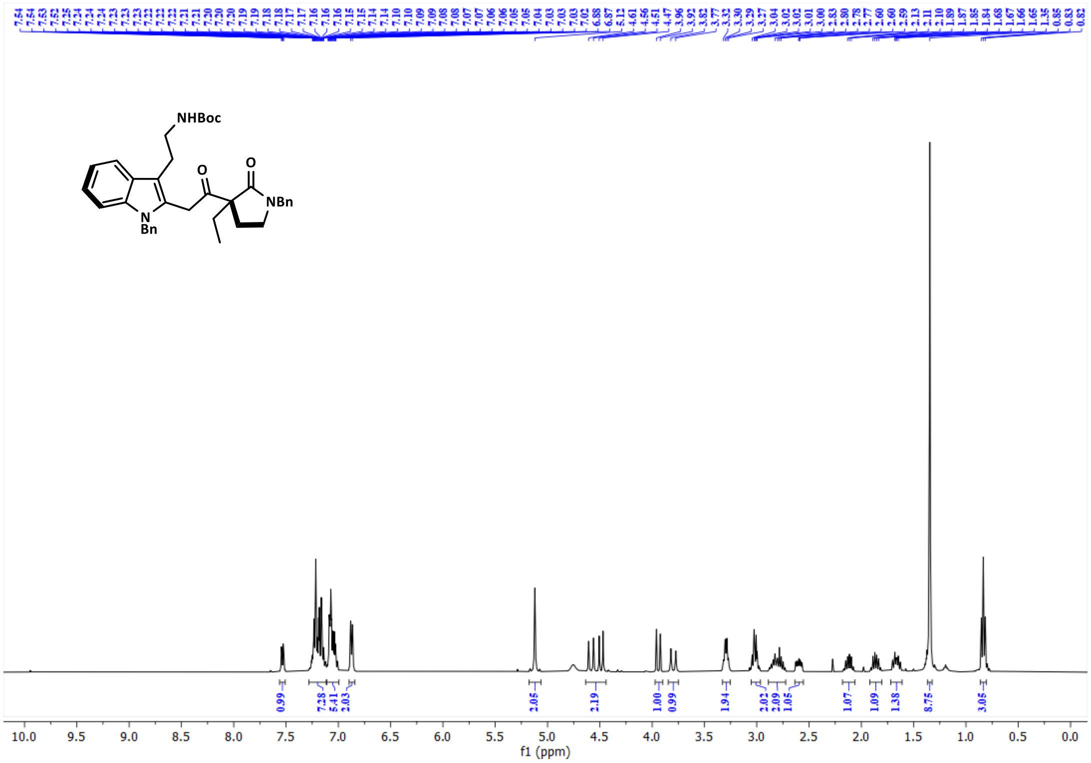

*Compound **22**. ^{13}C NMR (100 MHz, CHLOROFORM-D).*

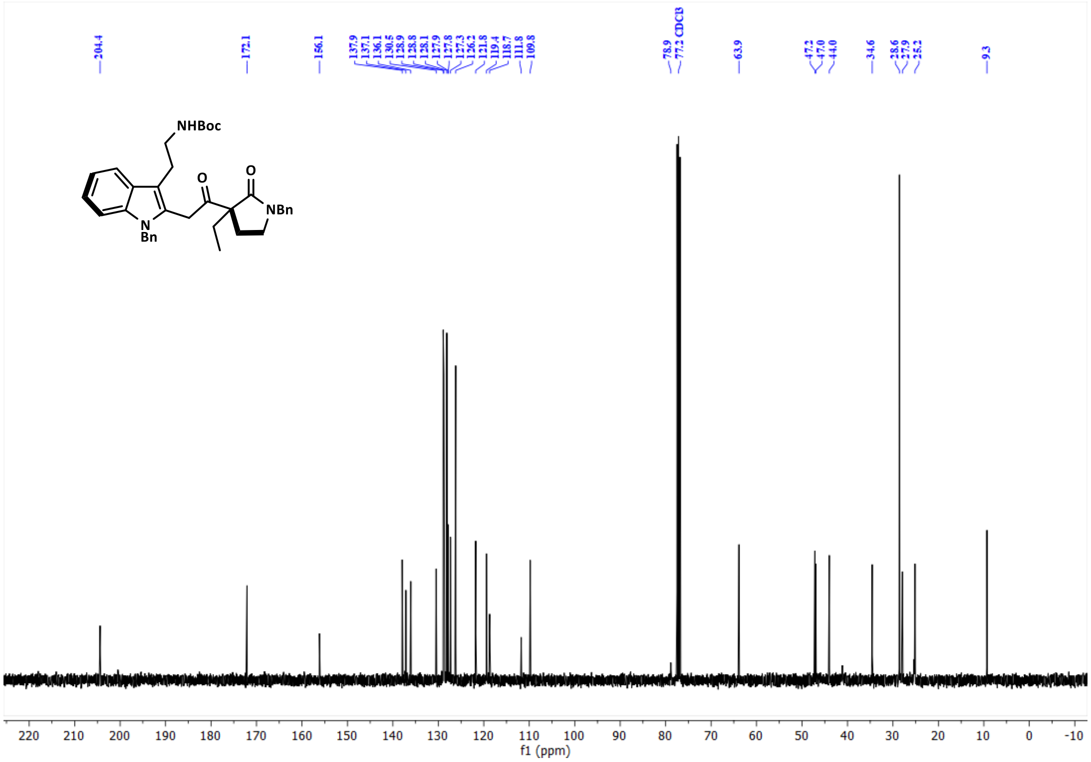

*Compound **23**. ^{1}H NMR (400 MHz, CHLOROFORM-D).*

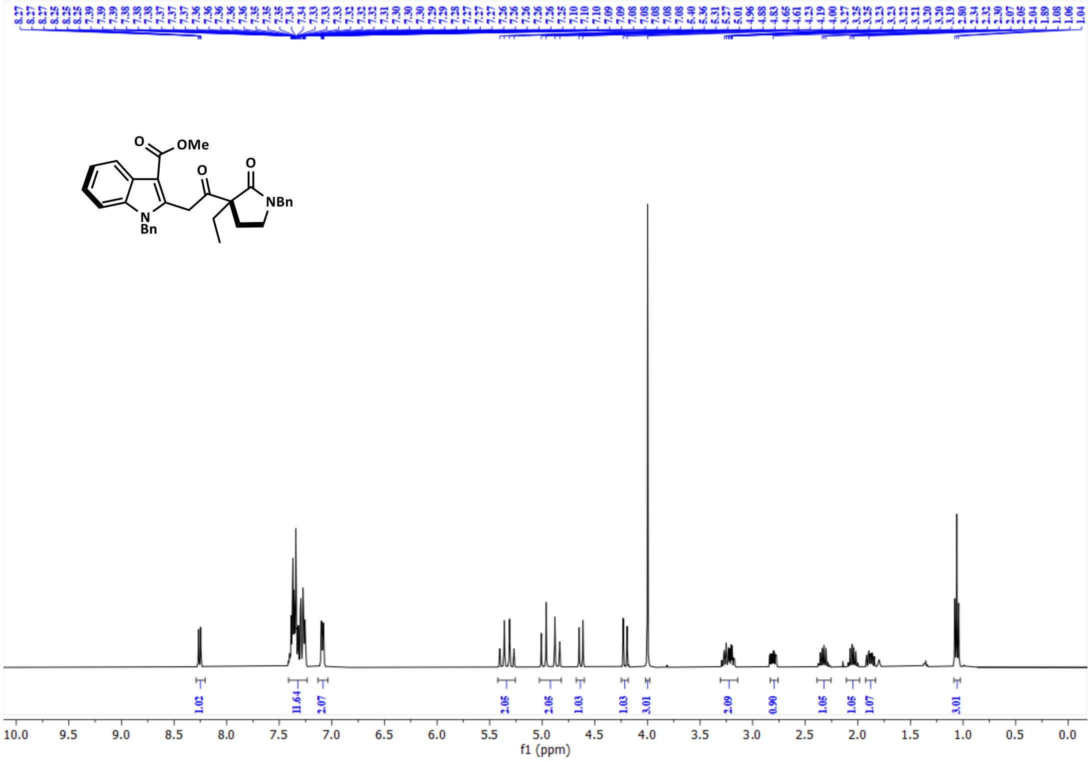

*Compound **23**. ^{13}C NMR (100 MHz, CHLOROFORM-D).*

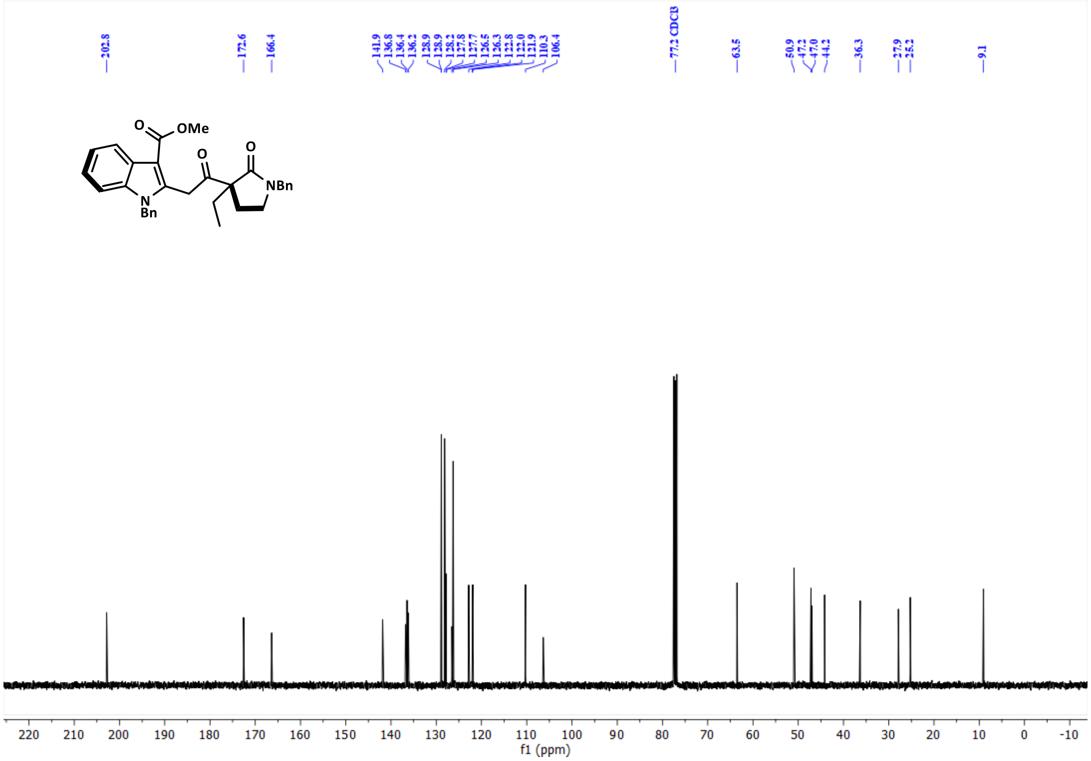

*Compound **24**. ^{1}H NMR (400 MHz, ACETONE-D$_6$).*

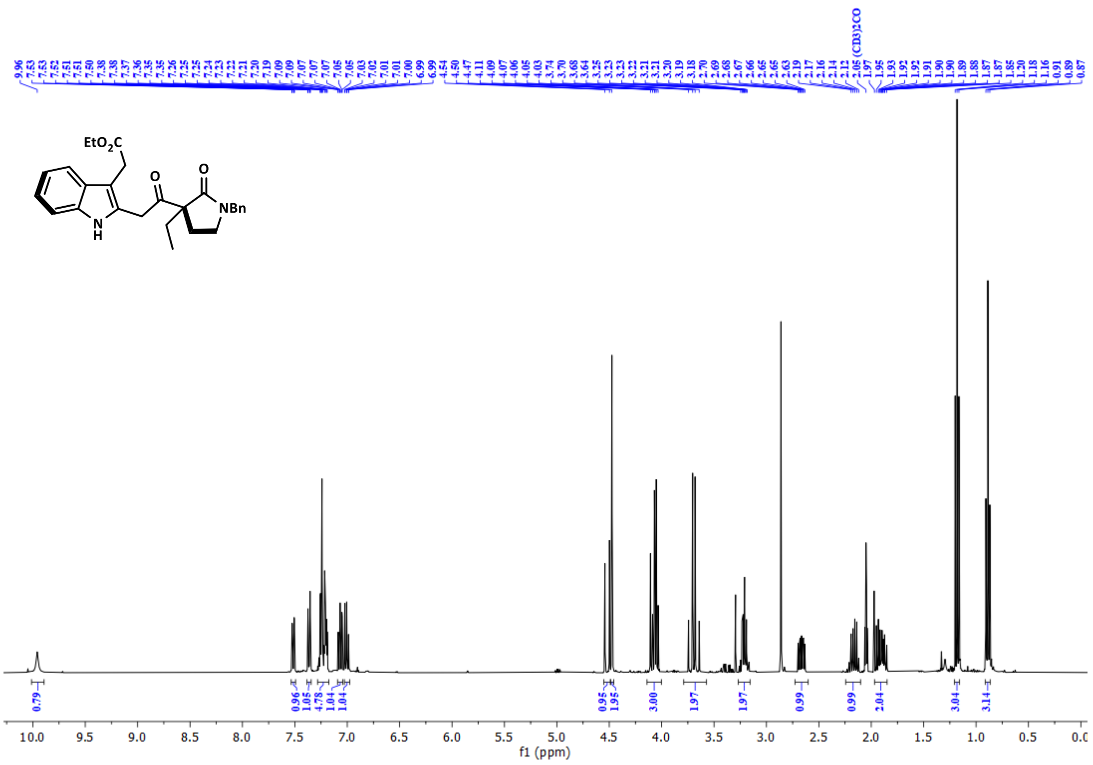

*Compound **24**. ^{13}C NMR (100 MHz, ACETONE-D$_6$).*

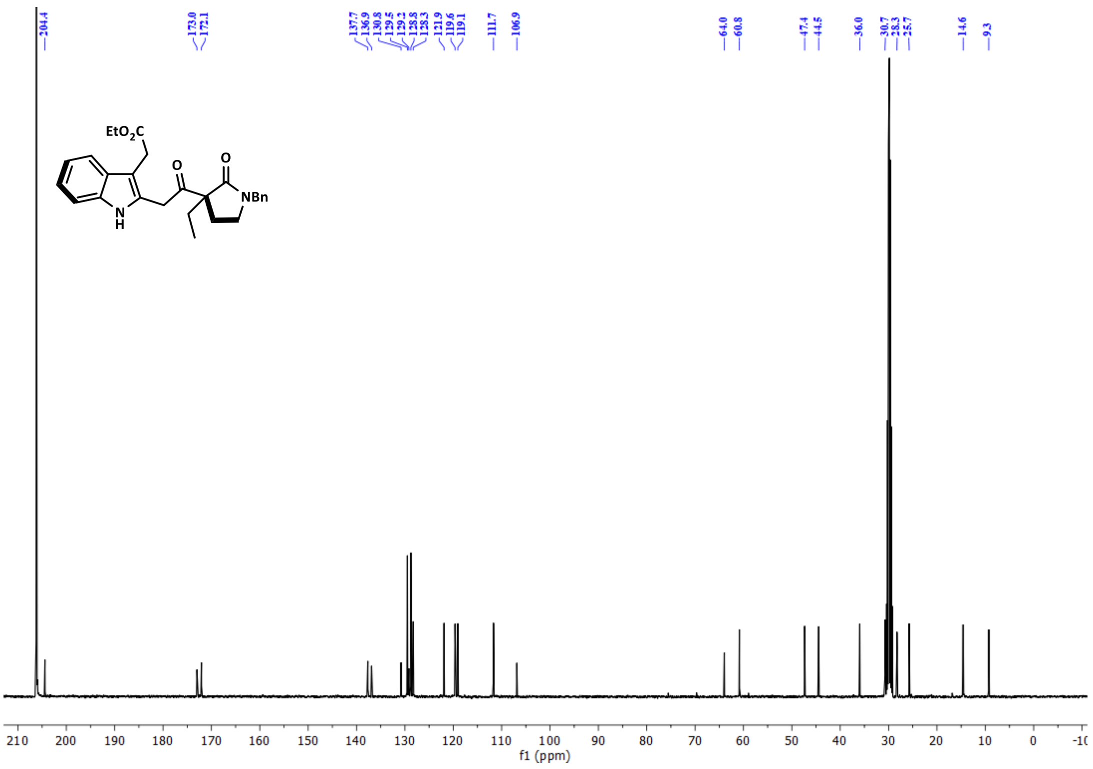

*Compound **25**. ^{1}H NMR (400 MHz, CHLOROFORM-D).*

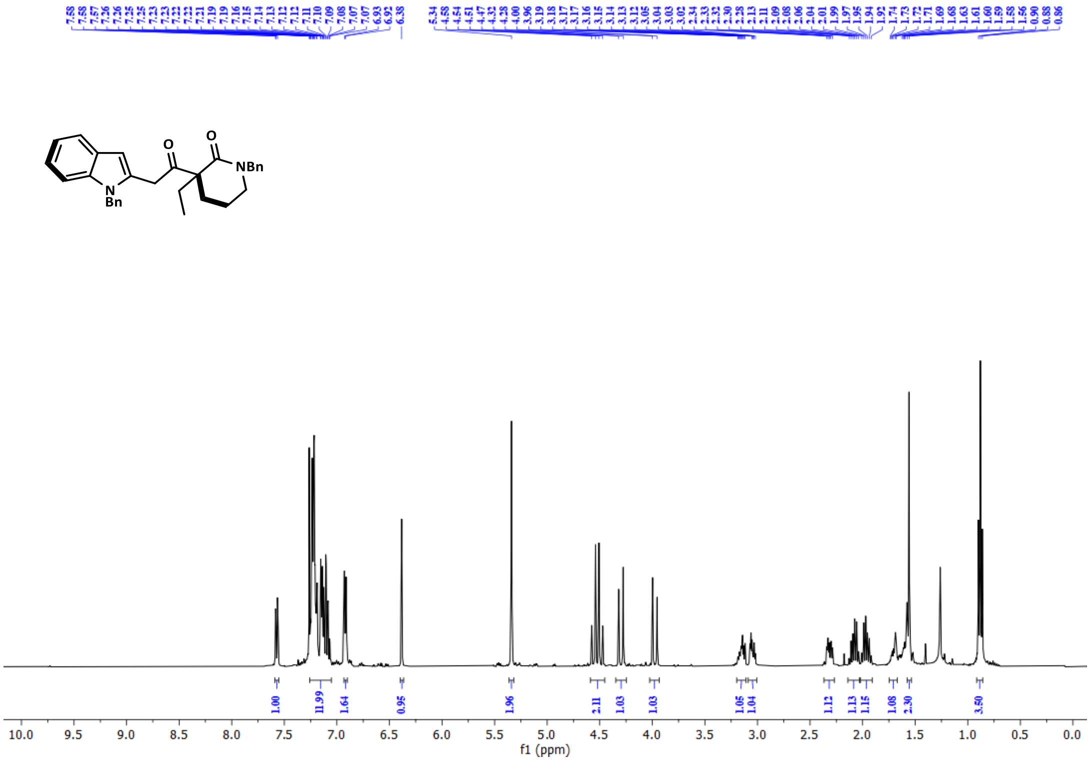

*Compound **25**. ^{13}C NMR (100 MHz, CHLOROFORM-D).*

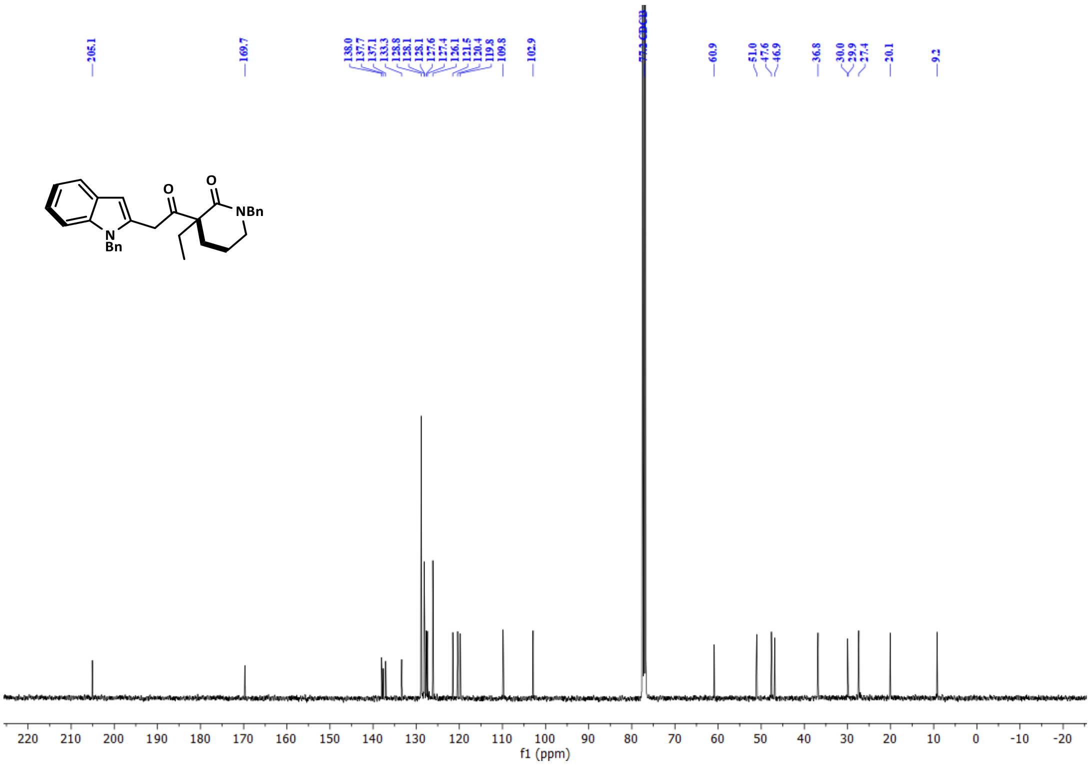

*Compound **26**. ^{1}H NMR (400 MHz, CHLOROFORM-D).*

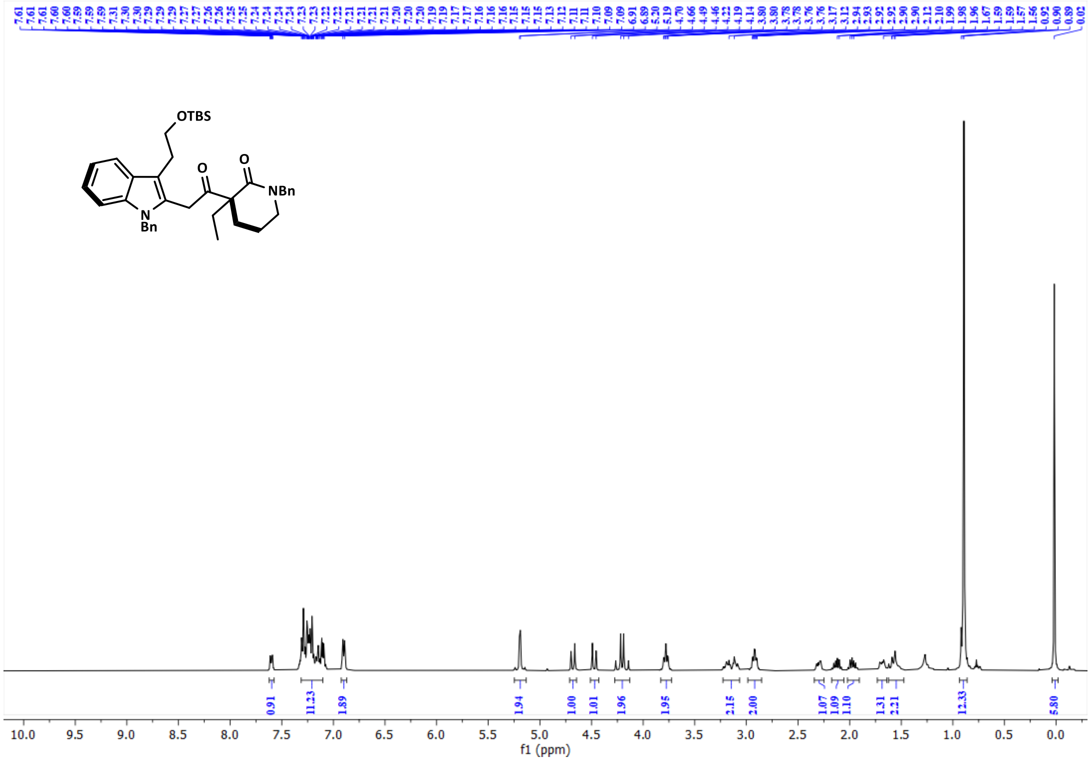

*Compound **26**. ^{13}C NMR (100 MHz, CHLOROFORM-D).*

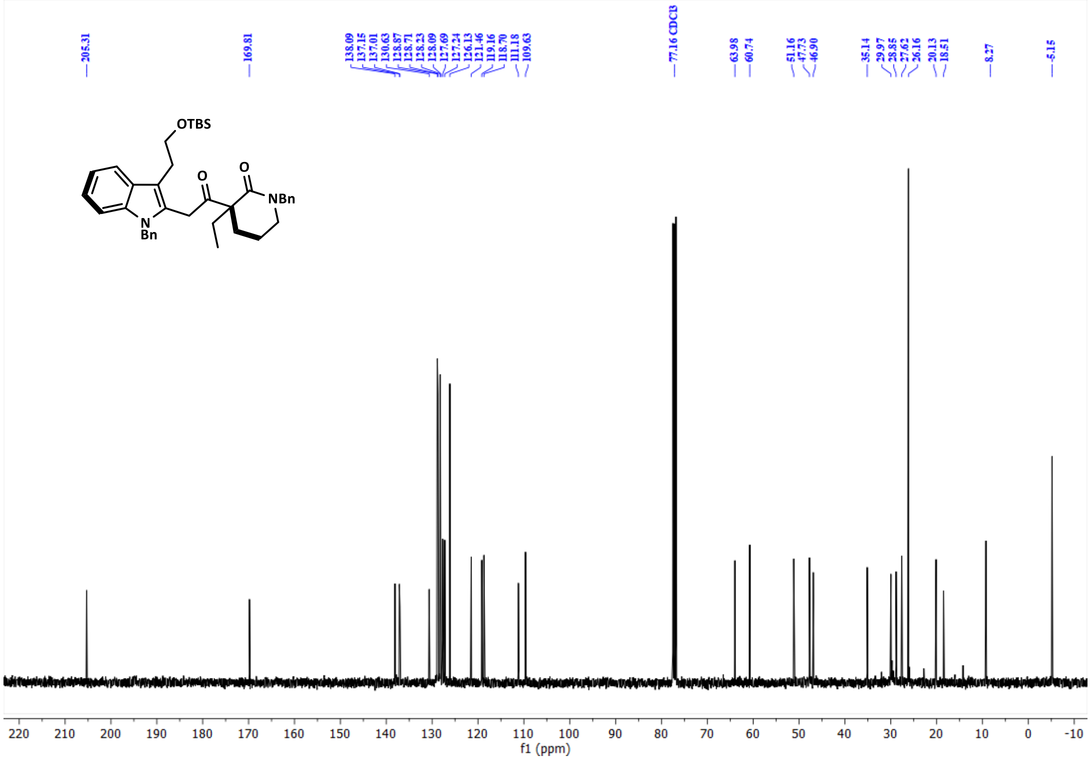

*Compound **27**.* ^{1}*H NMR (400 MHz, CHLOROFORM-D).*

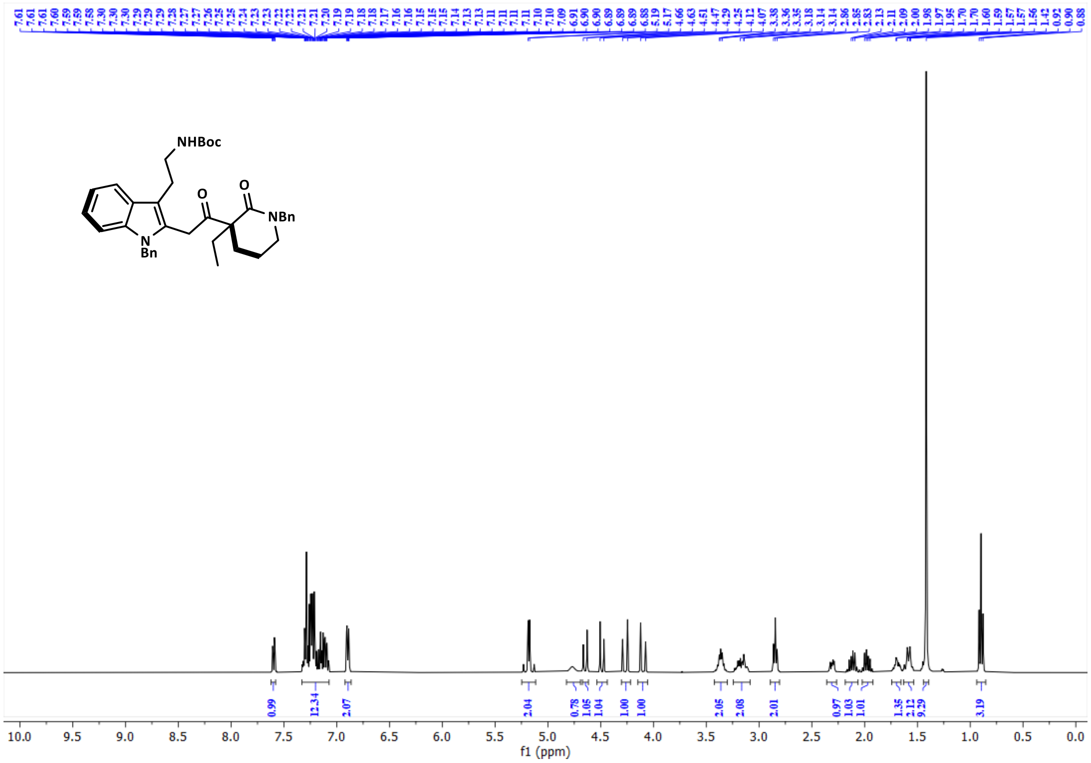

*Compound **27**.* ^{13}*C NMR (100 MHz, CHLOROFORM-D).*

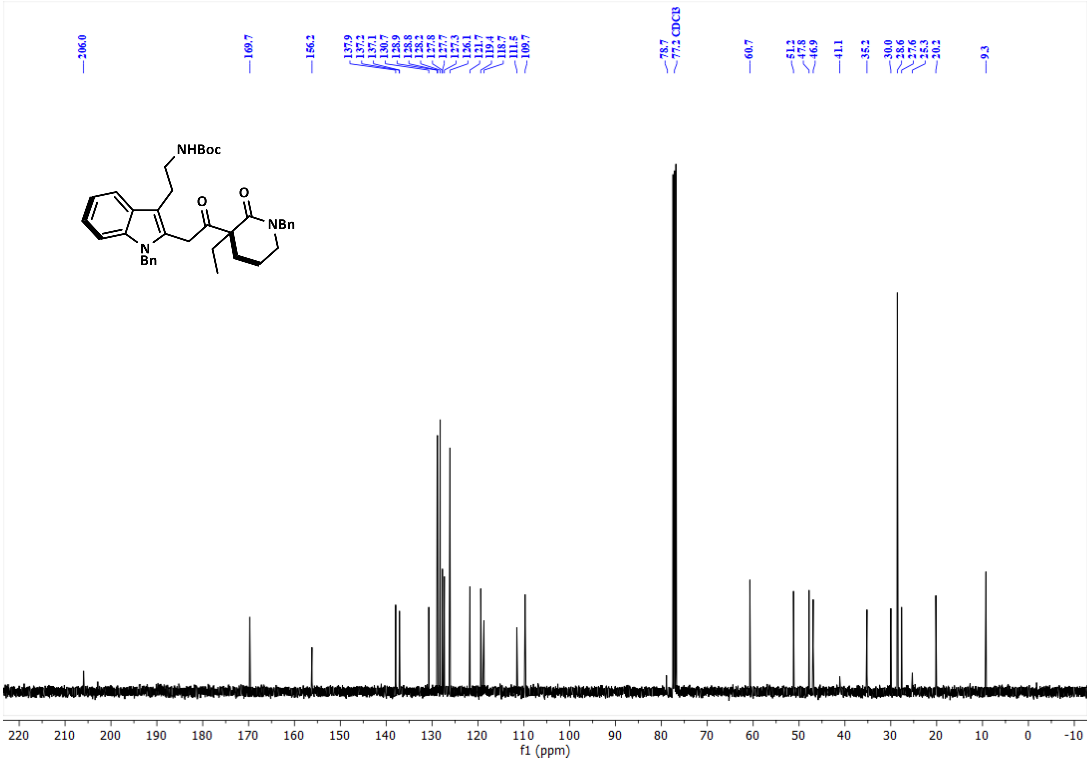

*Compound **28**. ^{1}H NMR (400 MHz, CHLOROFORM-D).*

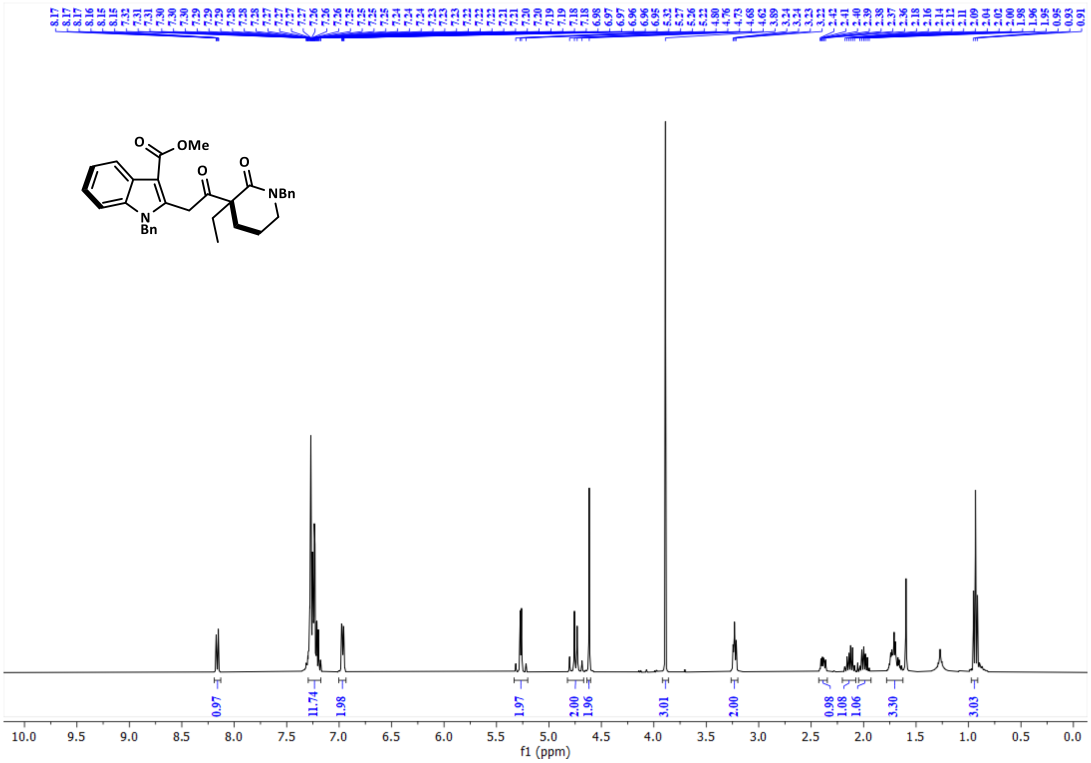

*Compound **28**. ^{13}C NMR (100 MHz, CHLOROFORM-D).*

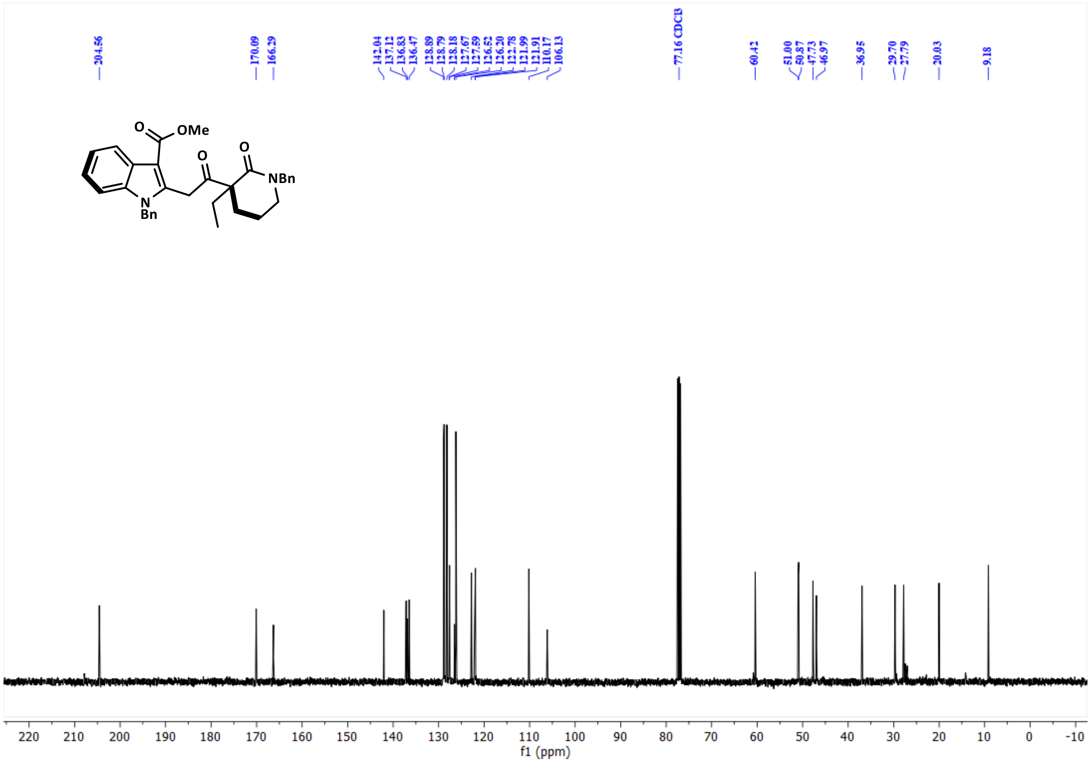

*Compound **29**.* ^{1}*H NMR (400 MHz, CHLOROFORM-D).*

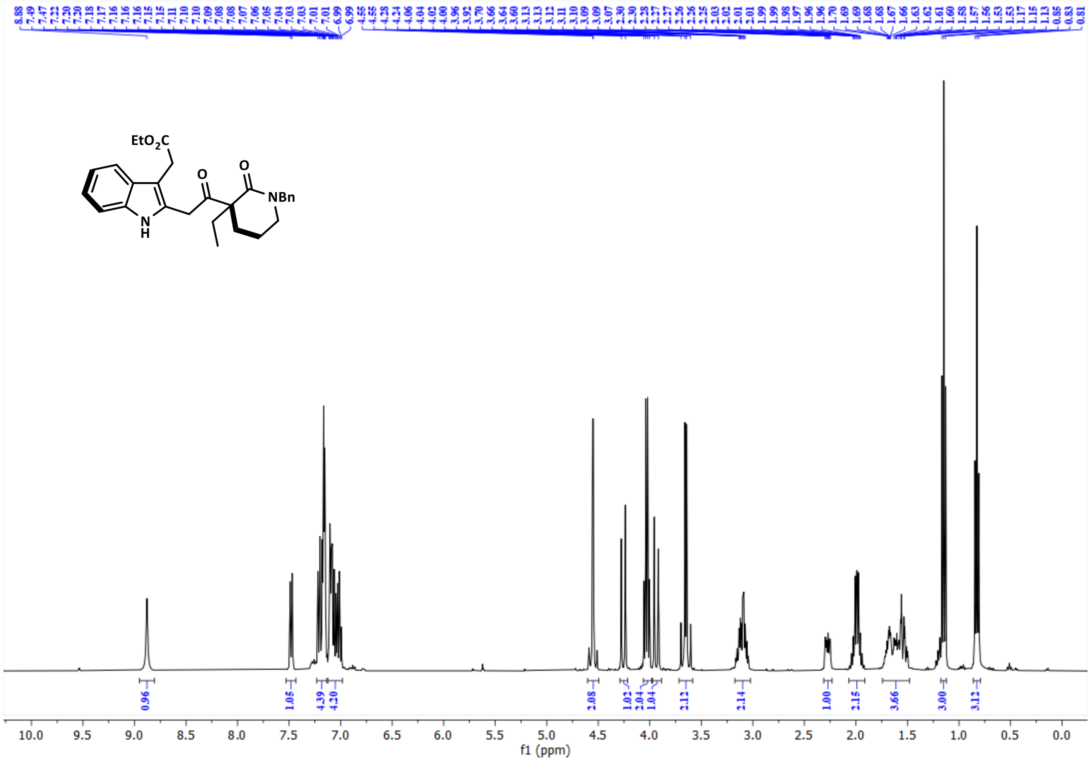

*Compound **29**.* ^{13}*C NMR (100 MHz, CHLOROFORM-D).*

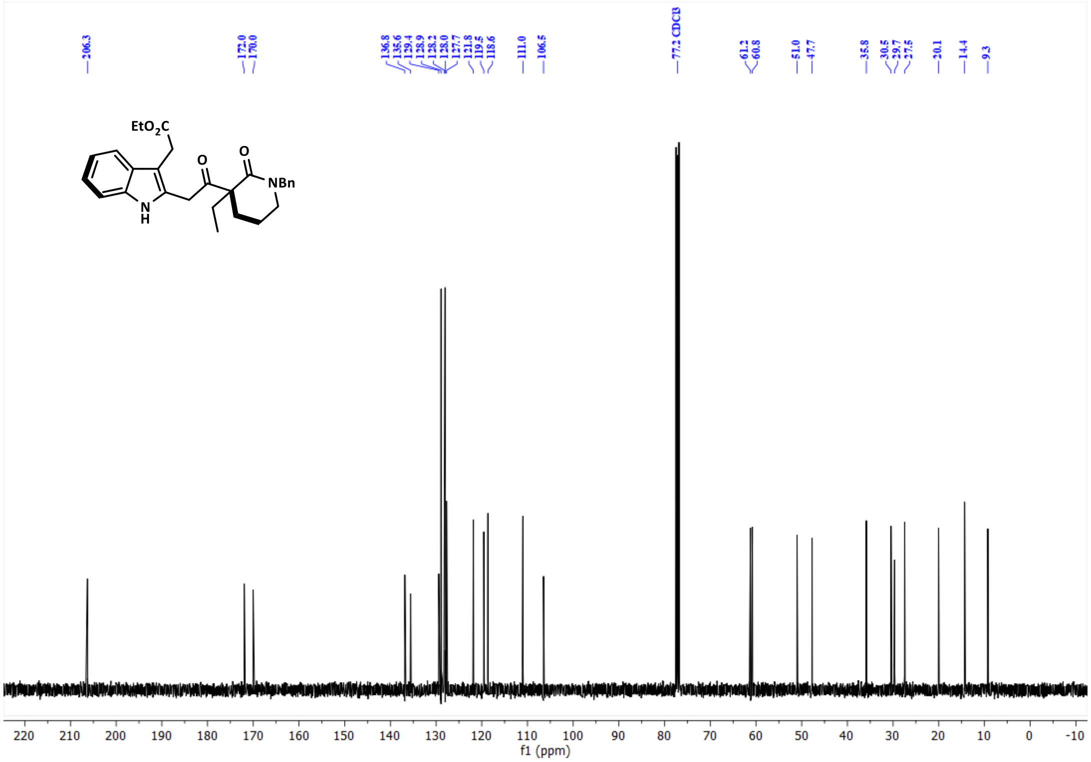

Compound ***30, diasteromer 1****.* *^{1}H NMR (400 MHz, CHLOROFORM-D).*

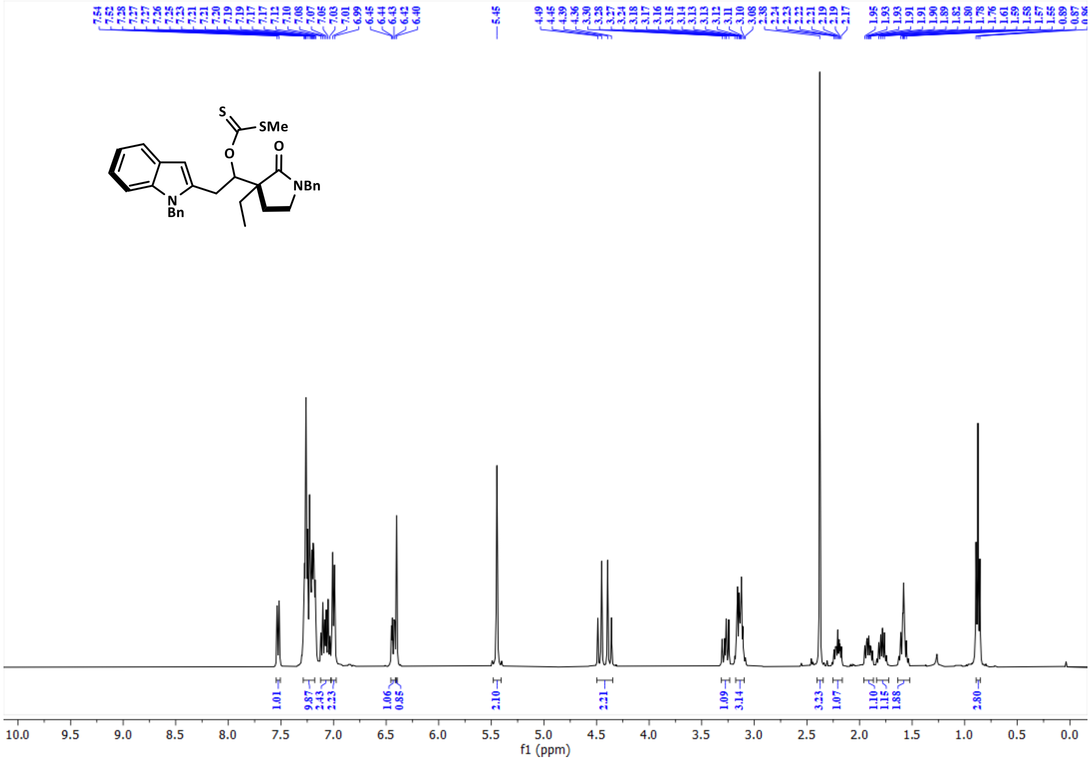

Compound ***30, diasteromer 2****.* *^{1}H NMR (400 MHz, CHLOROFORM-D).*

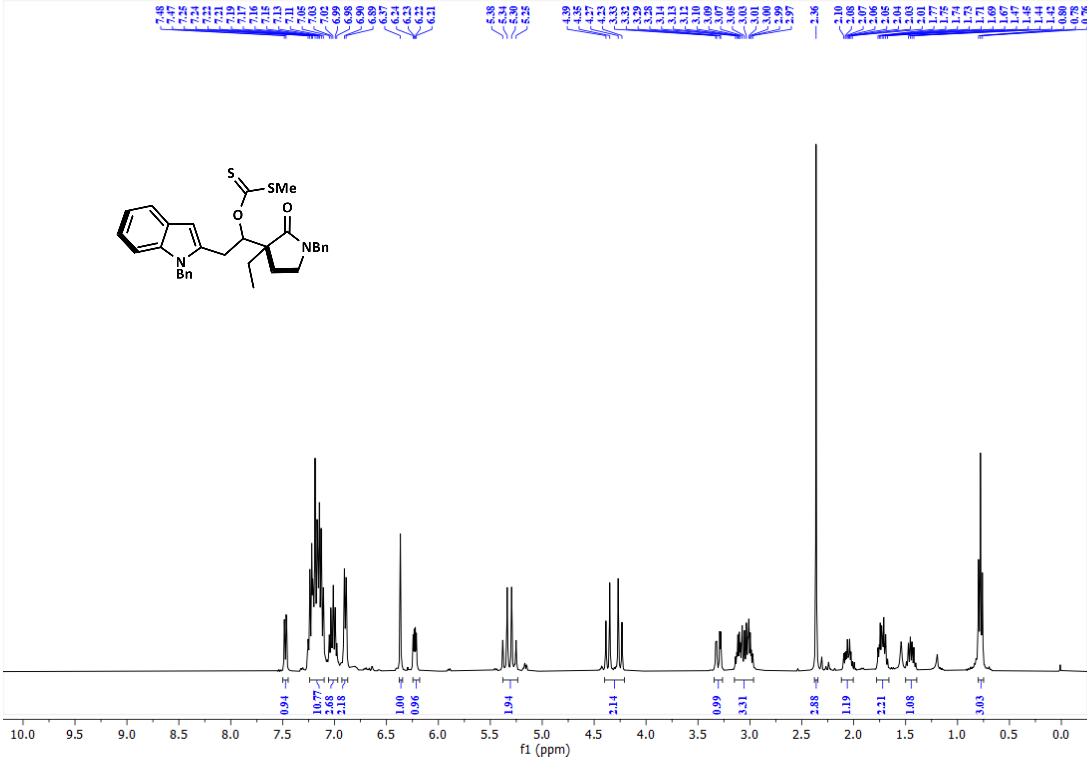

*Compound **30, diasteromer 1**.* *^{13}C NMR (100 MHz, CHLOROFORM-D).*

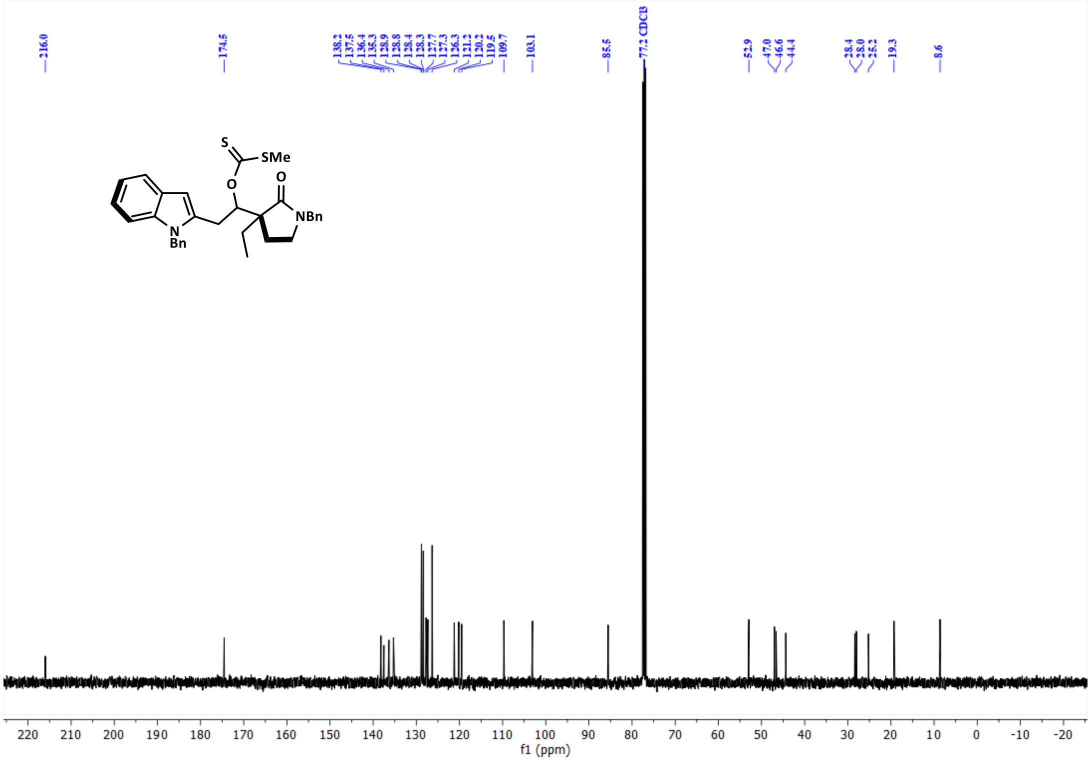

*Compound **30, diasteromer 2**.* *^{13}C NMR (100 MHz, CHLOROFORM-D).*

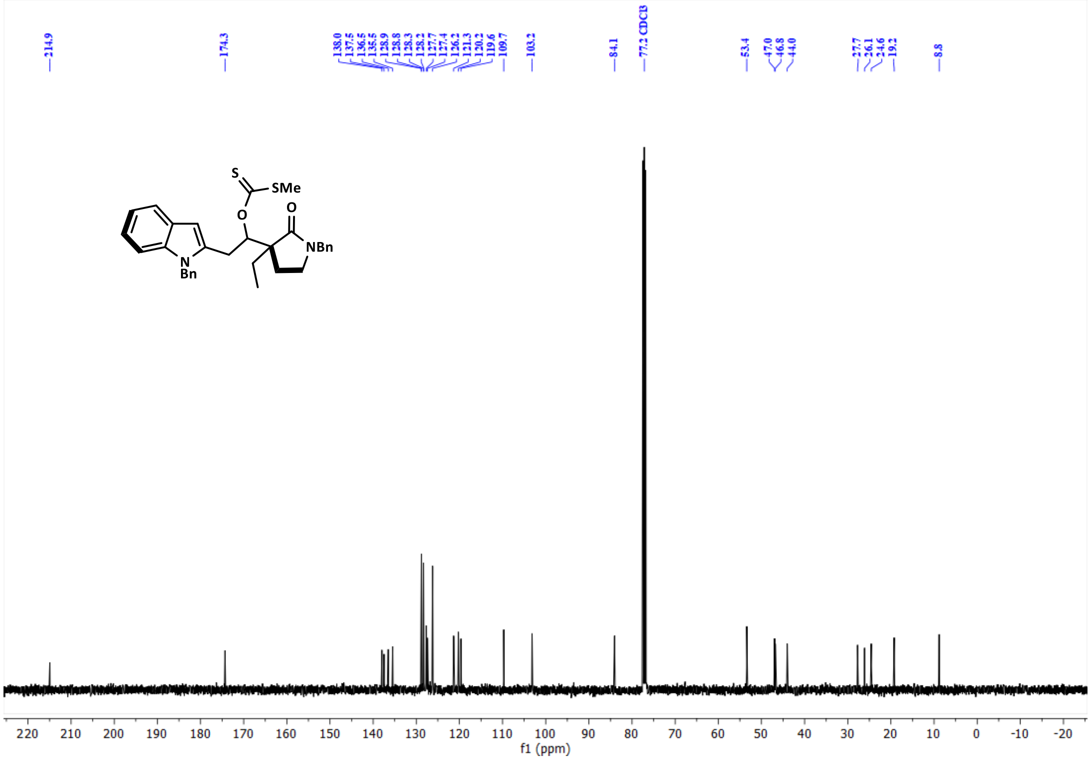

*Compound **31, diasterome r 1**. ^{1}H NMR (400 MHz, ACETONE-D$_6$).*

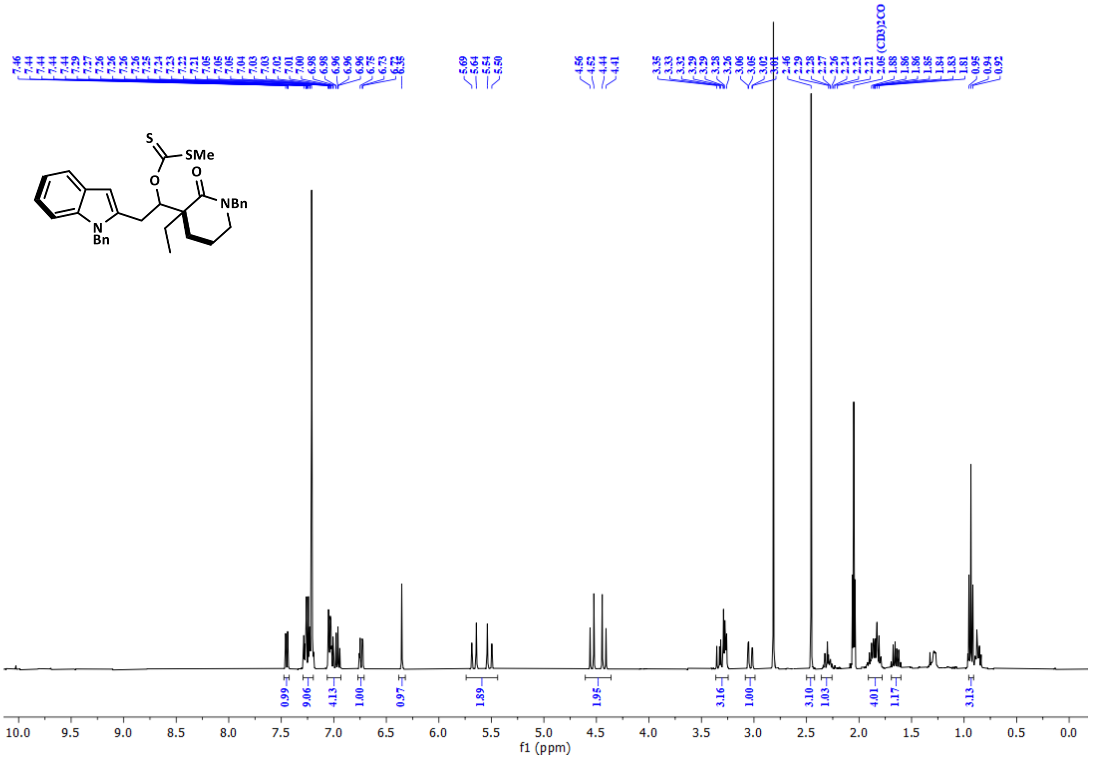

*Compound **31, diastereomer 2**. ^{1}H NMR (400 MHz, ACETONE-D$_6$).*

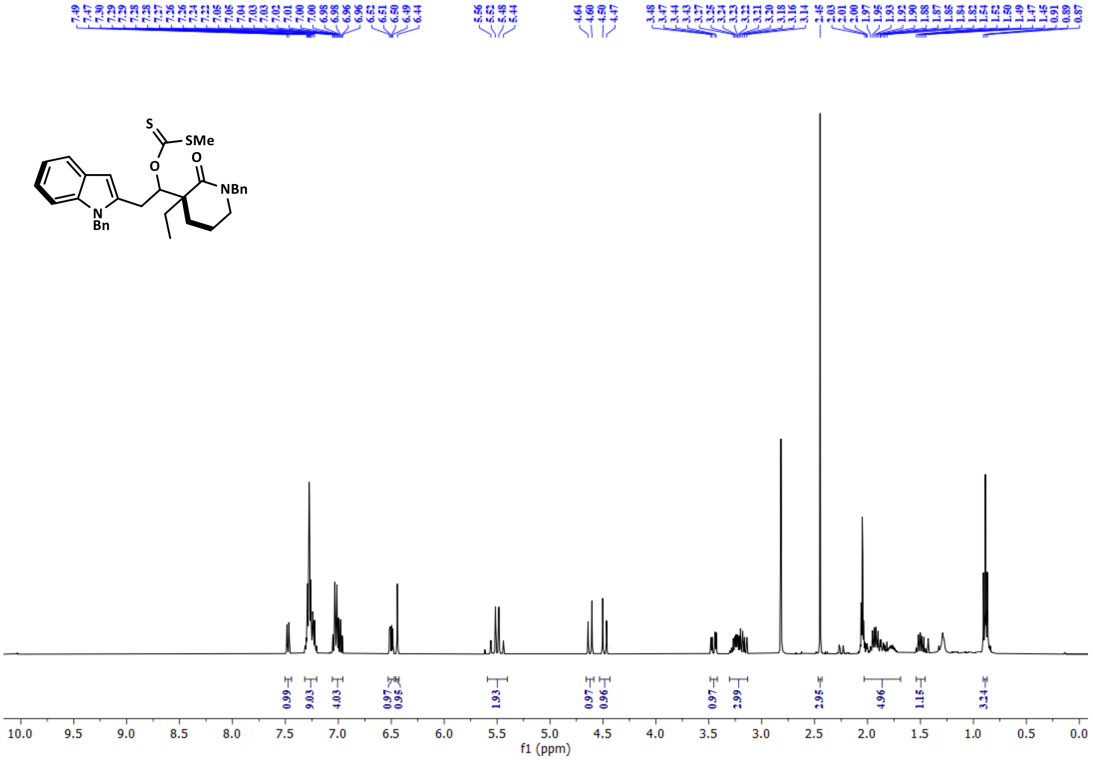

*Compound **31, diasteromer 1**.* *^{13}C NMR (100 MHz, ACETONE-D$_6$).*

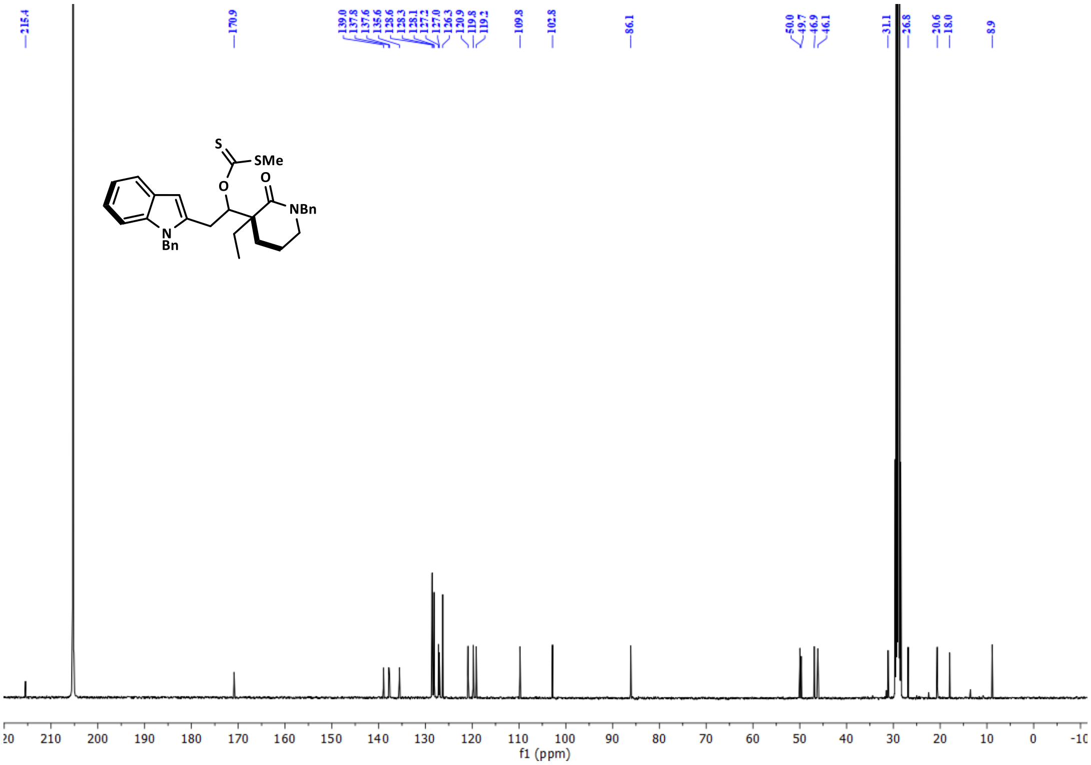

*Compound **31, diasteromer 2**.* *^{13}C NMR (100 MHz, ACETONE-D$_6$).*

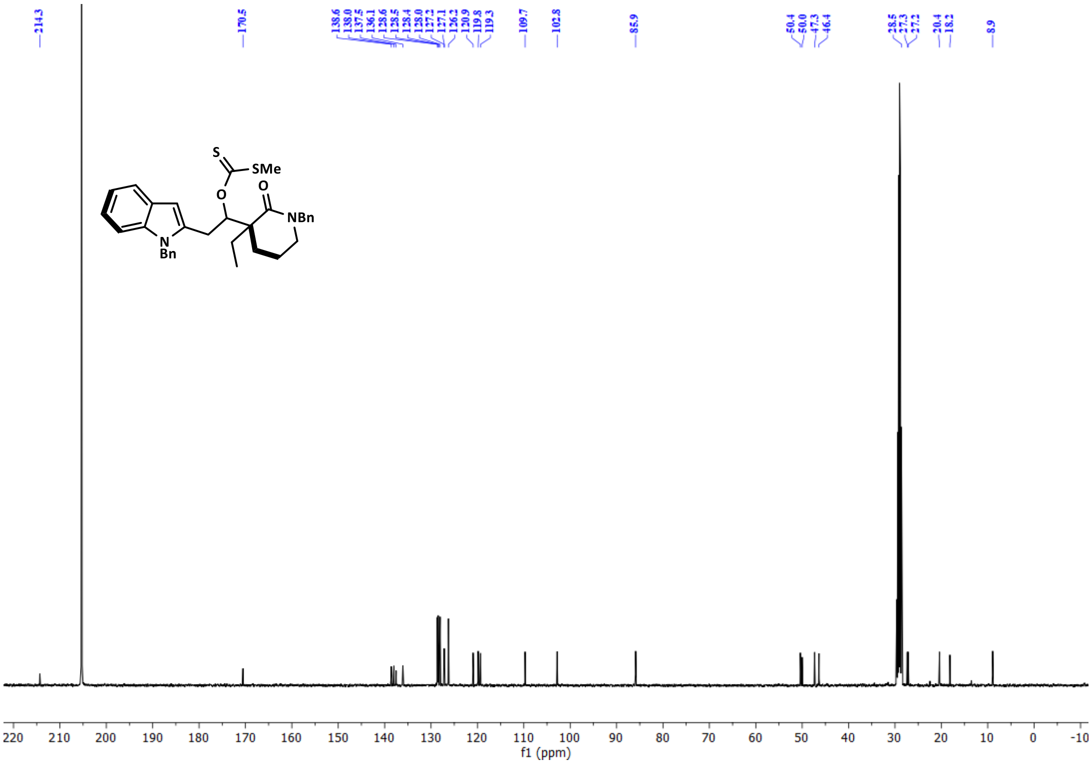

*Compound **32, diasterome r 1**. ^{1}H NMR (400 MHz, CHLOROFORM-D).*

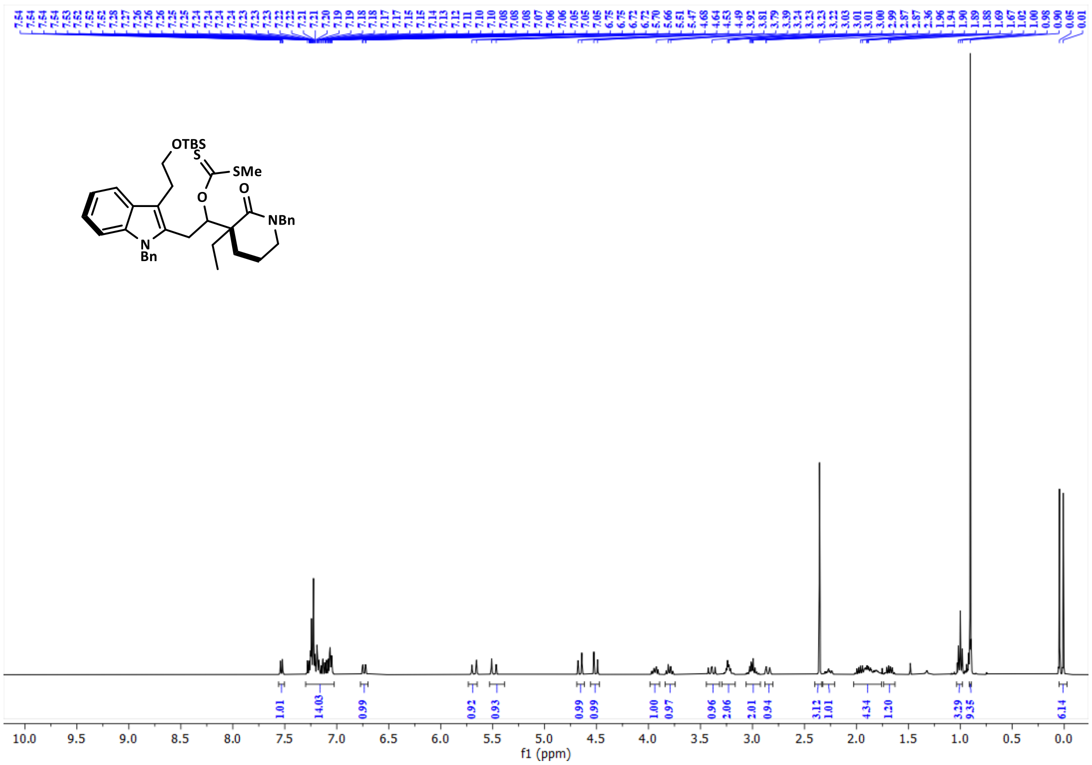

*Compound **32, diastereomer 1**. ^{1}H NMR (400 MHz, CHLOROFORM-D).*

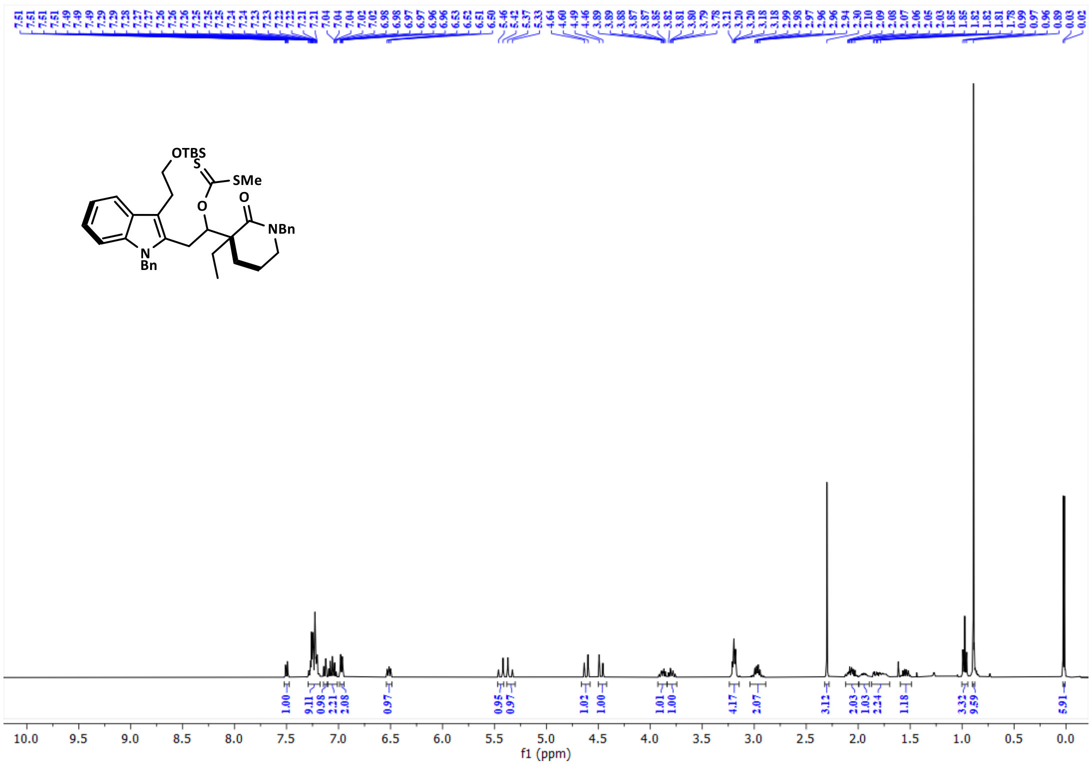

*Compound **32, diasteromer 2**.* *^{13}C NMR (100 MHz, CHLOROFORM-D).*

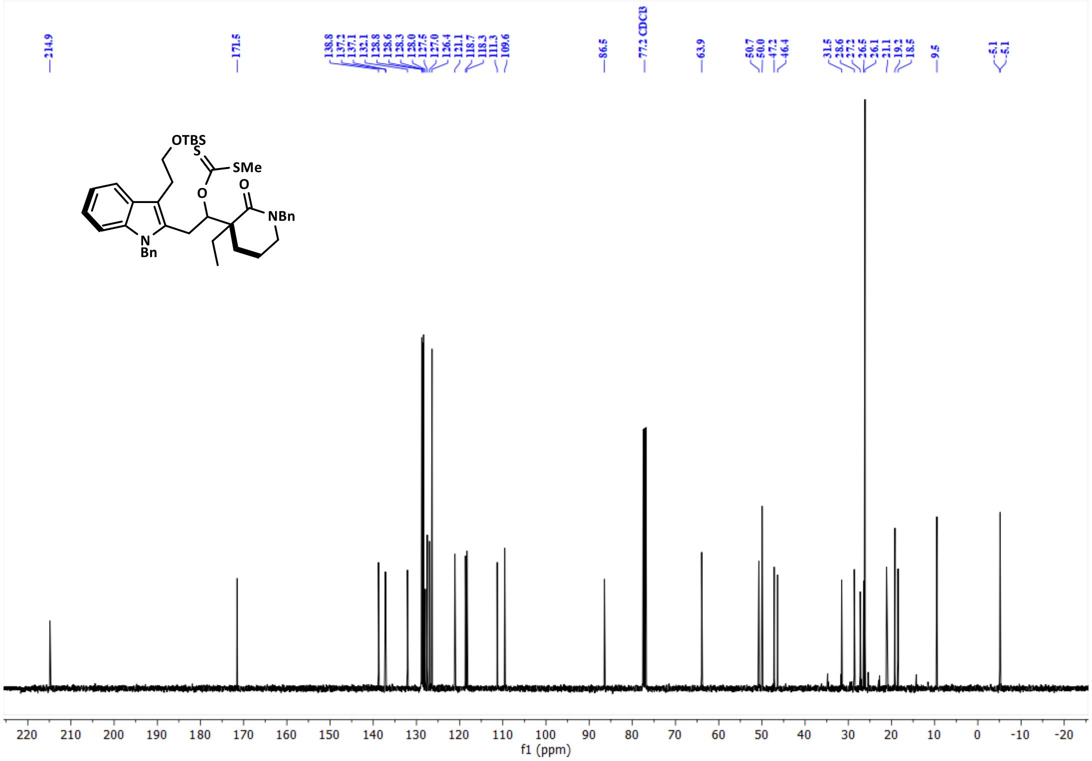

*Compound **32, diasteromer 2**.* *^{13}C NMR (100 MHz, CHLOROFORM-D).*

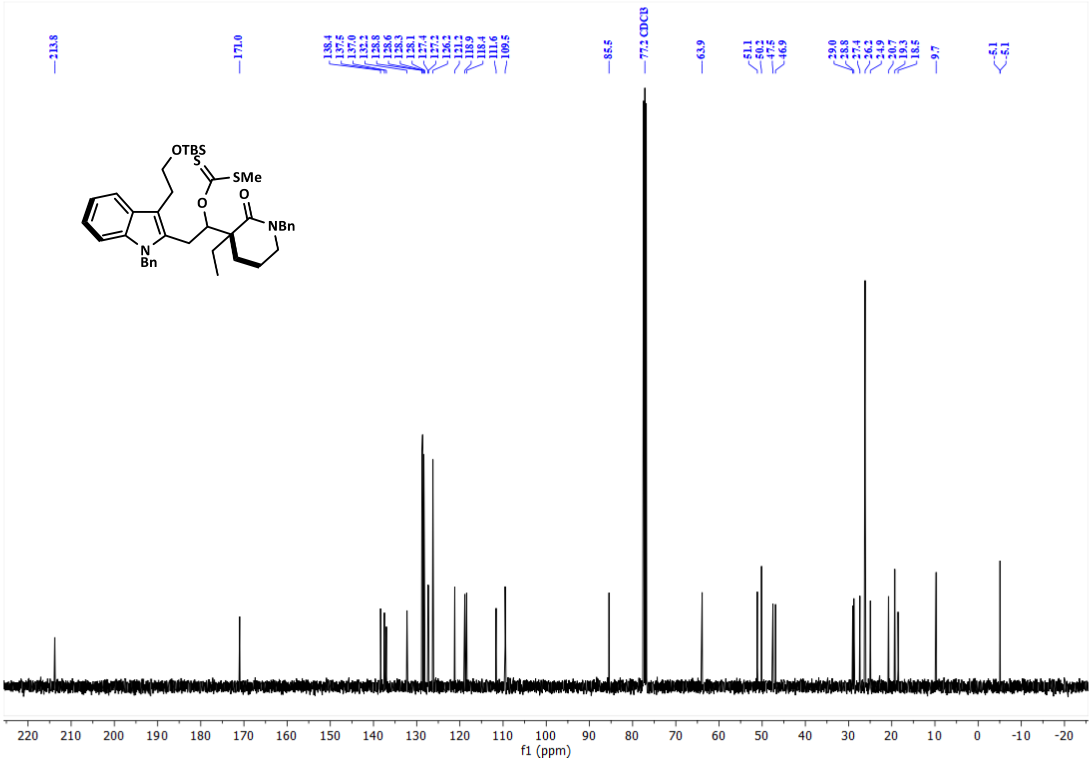

*Compound **33**. ^{1}H NMR (400 MHz, CHLOROFORM-D).*

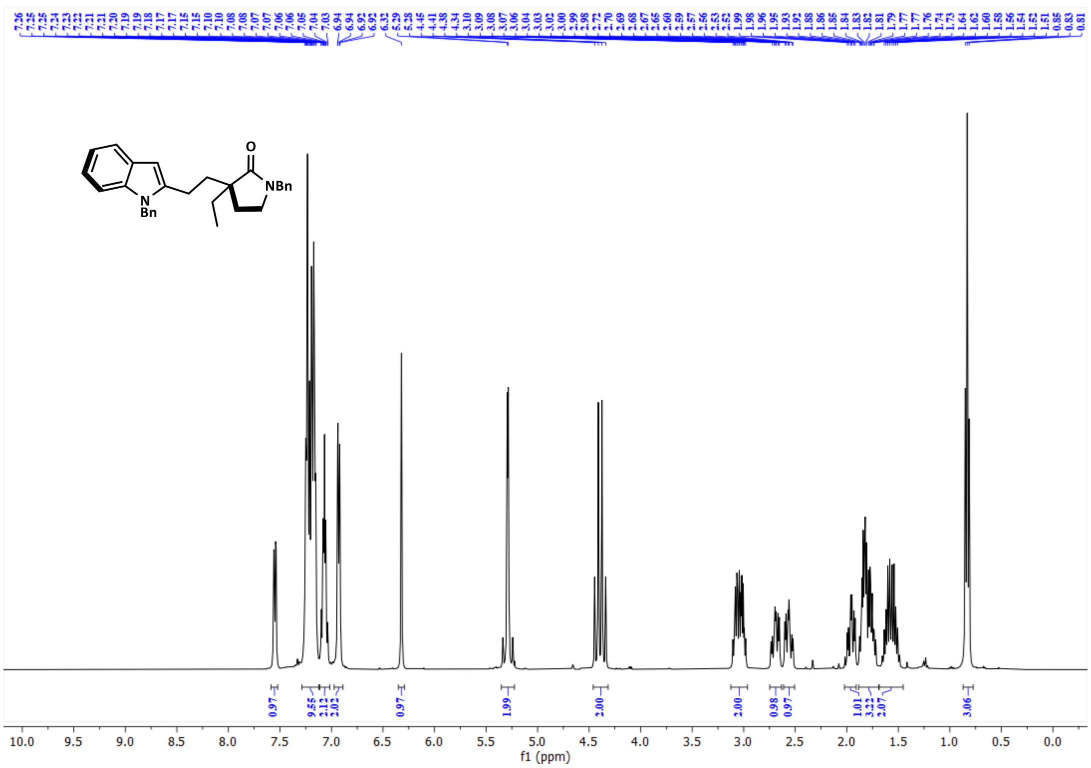

*Compound **33**. ^{13}C NMR (100 MHz, CHLOROFORM-D).*

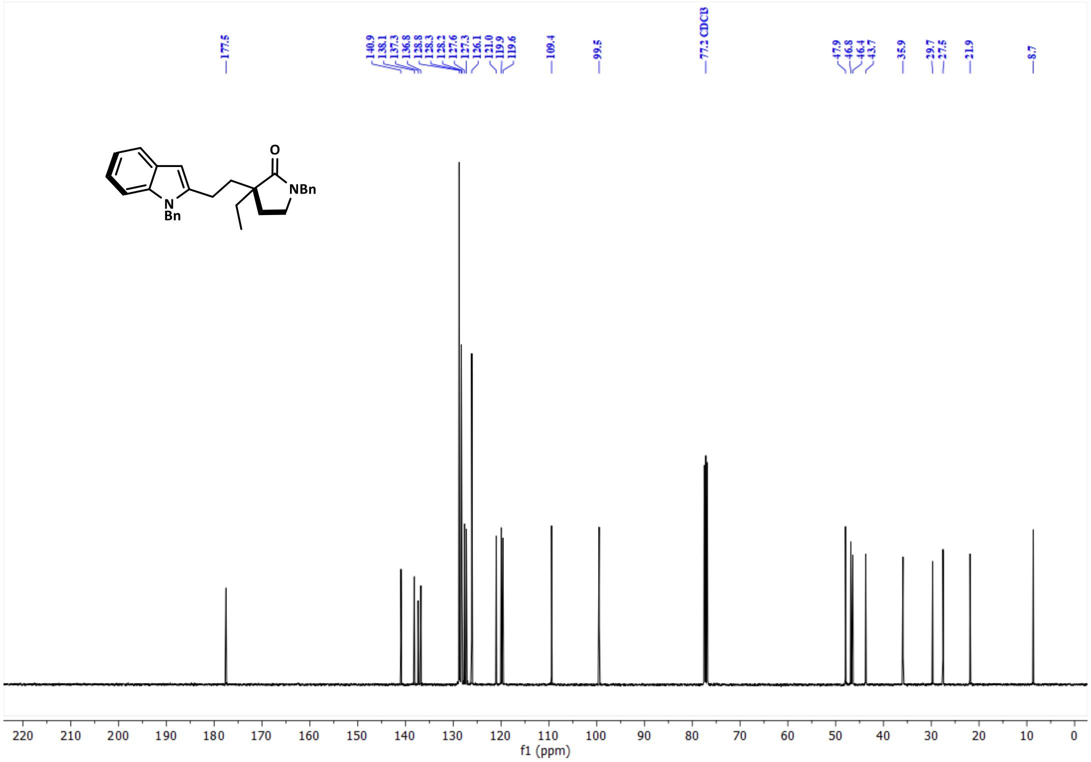

*Compound **34**. ^{1}H NMR (400 MHz, CHLOROFORM-D).*

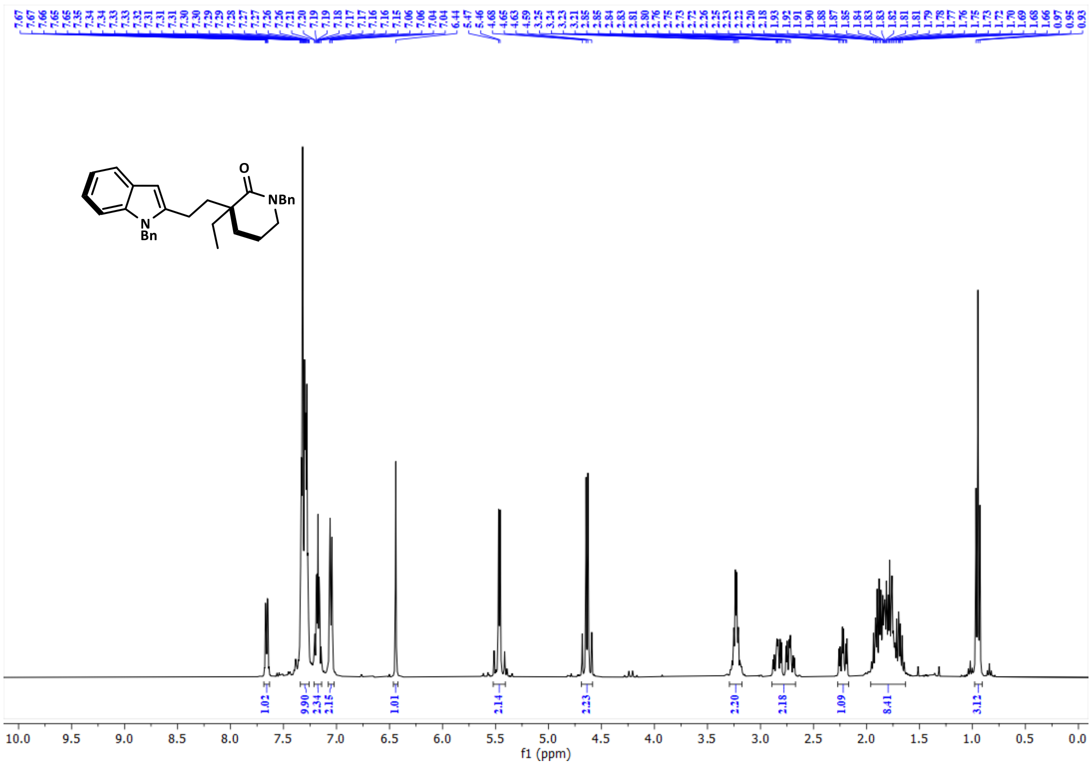

*Compound **34**. ^{13}C NMR (100 MHz, CHLOROFORM-D).*

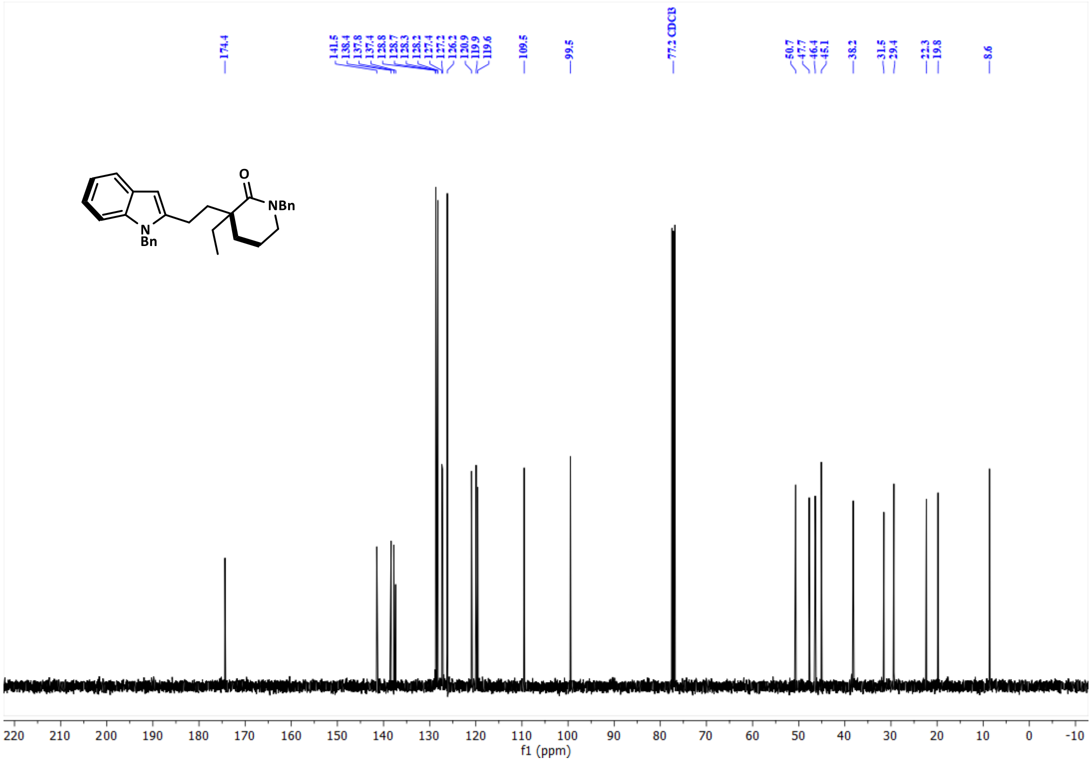

*Compound **35**. ^{1}H NMR (400 MHz, CHLOROFORM-D).*

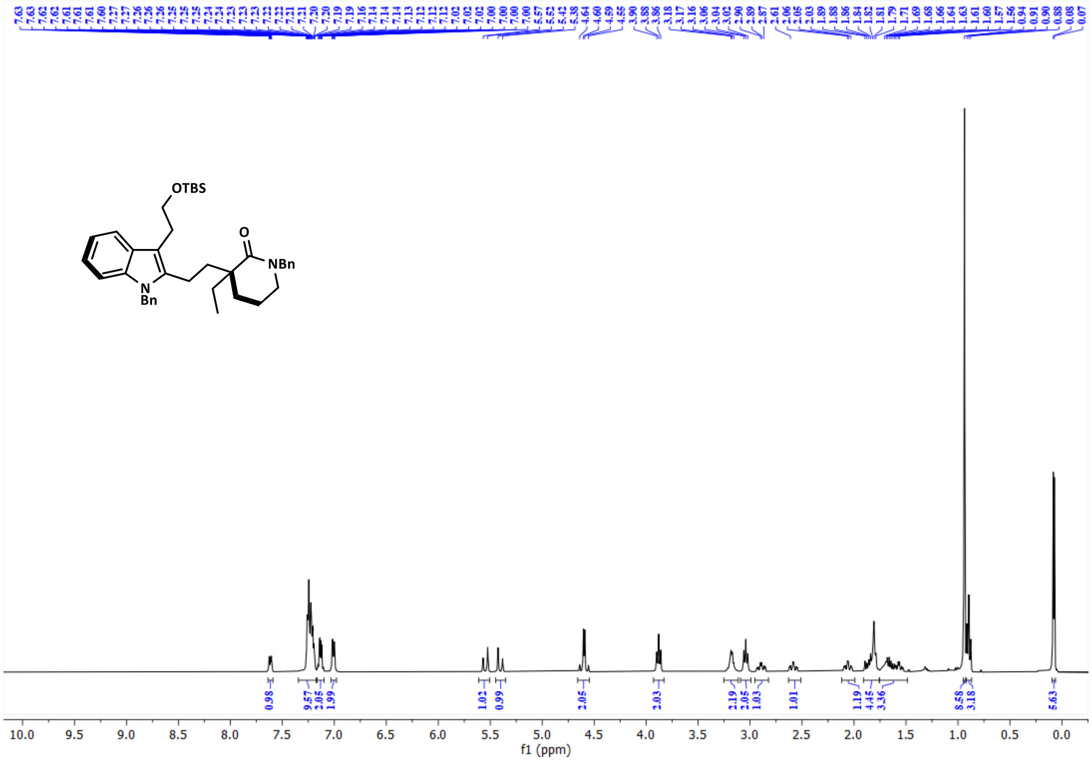

*Compound **35**. ^{13}C NMR (100 MHz, CHLOROFORM-D).*

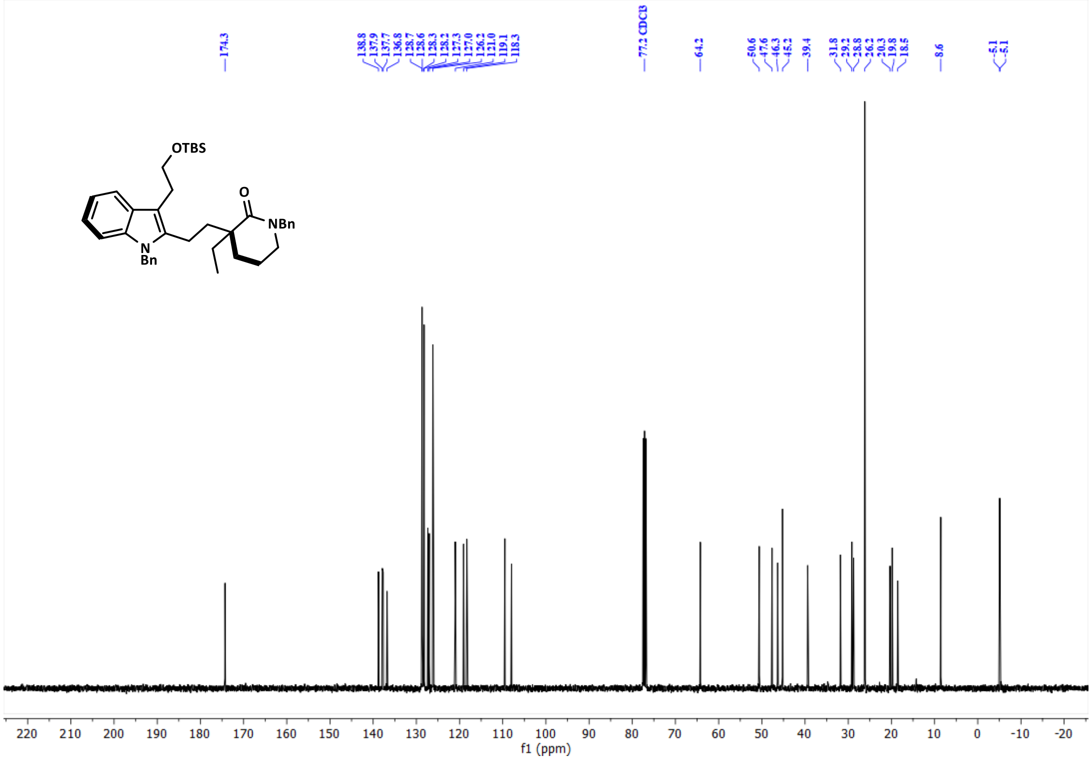

*Compound **36**. ^{1}H NMR (400 MHz, CHLOROFORM-D).*

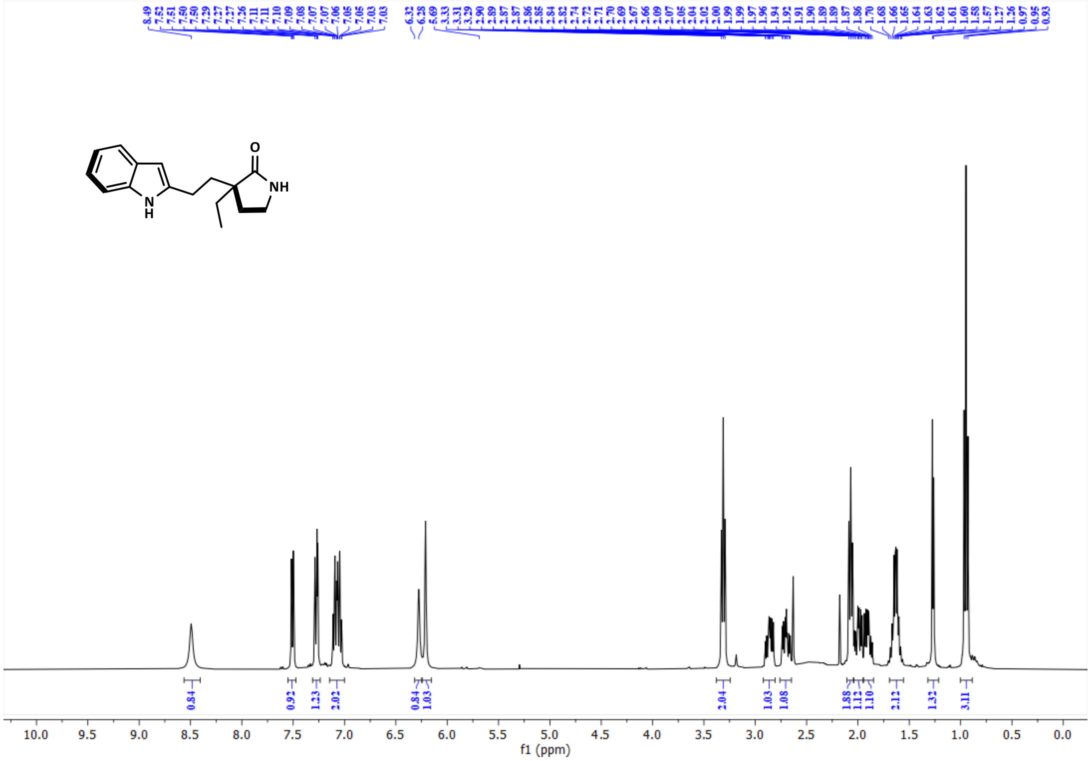

*Compound **36**. ^{13}C NMR (100 MHz, CHLOROFORM-D).*

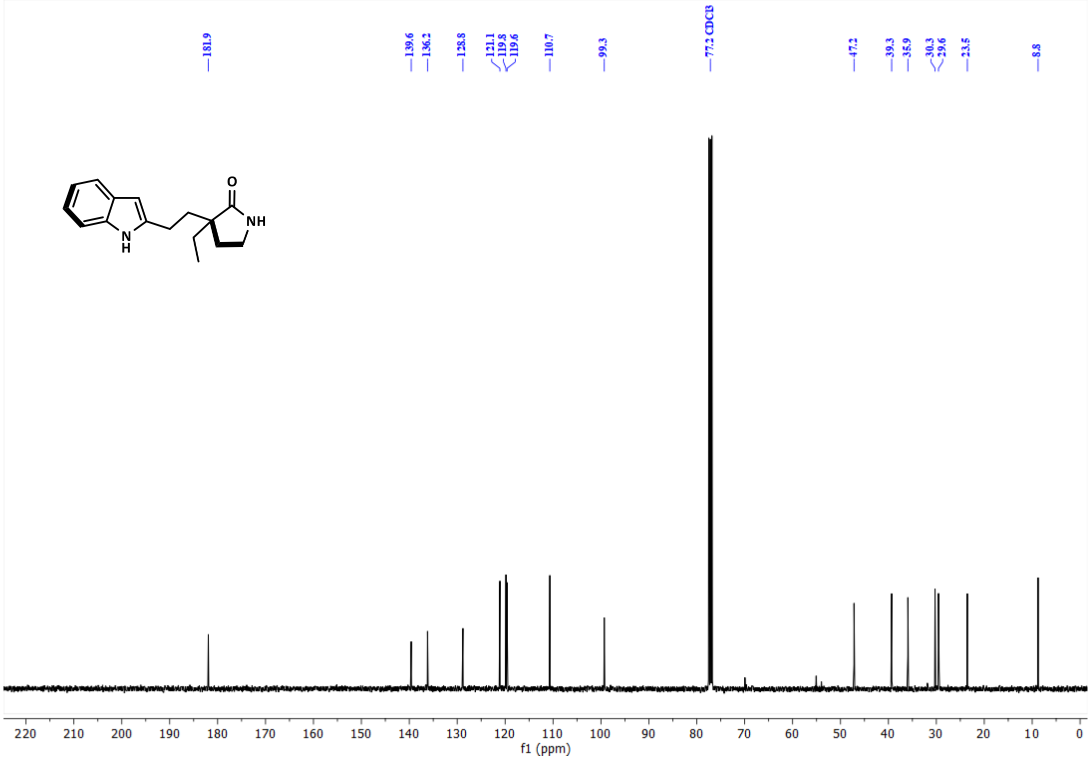

Compound **37**. ^{1}H NMR (400 MHz, CHLOROFORM-D).

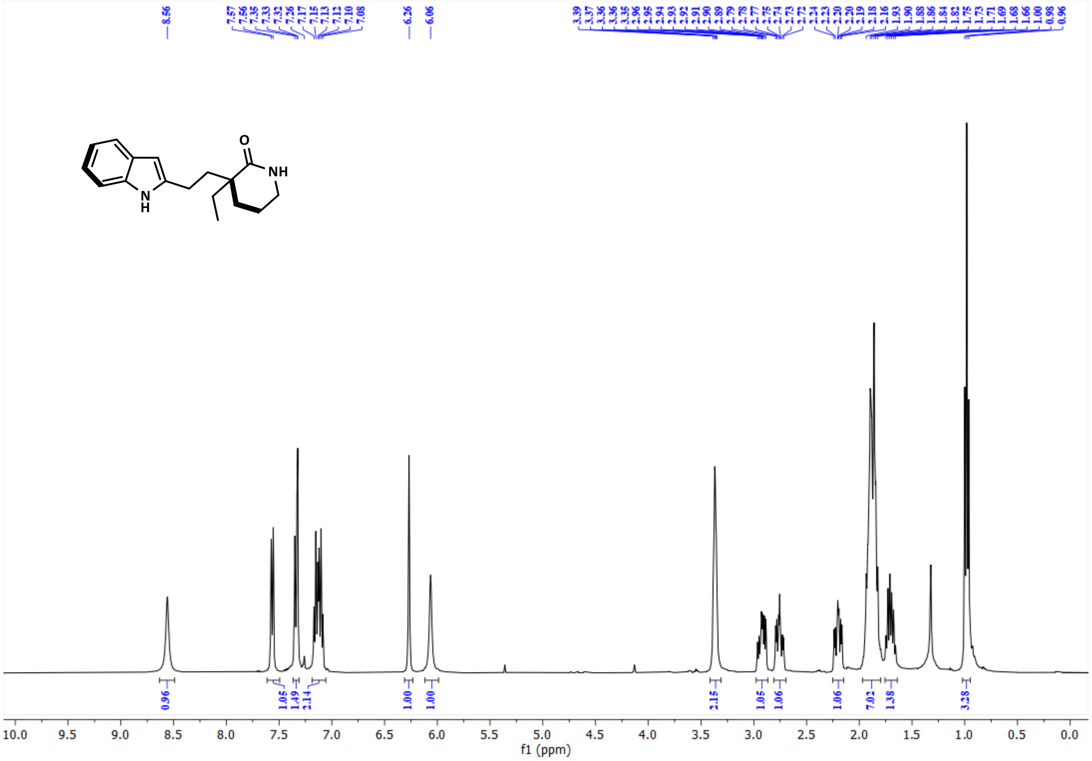

Compound **37**. ^{13}C NMR (100 MHz, CHLOROFORM-D).

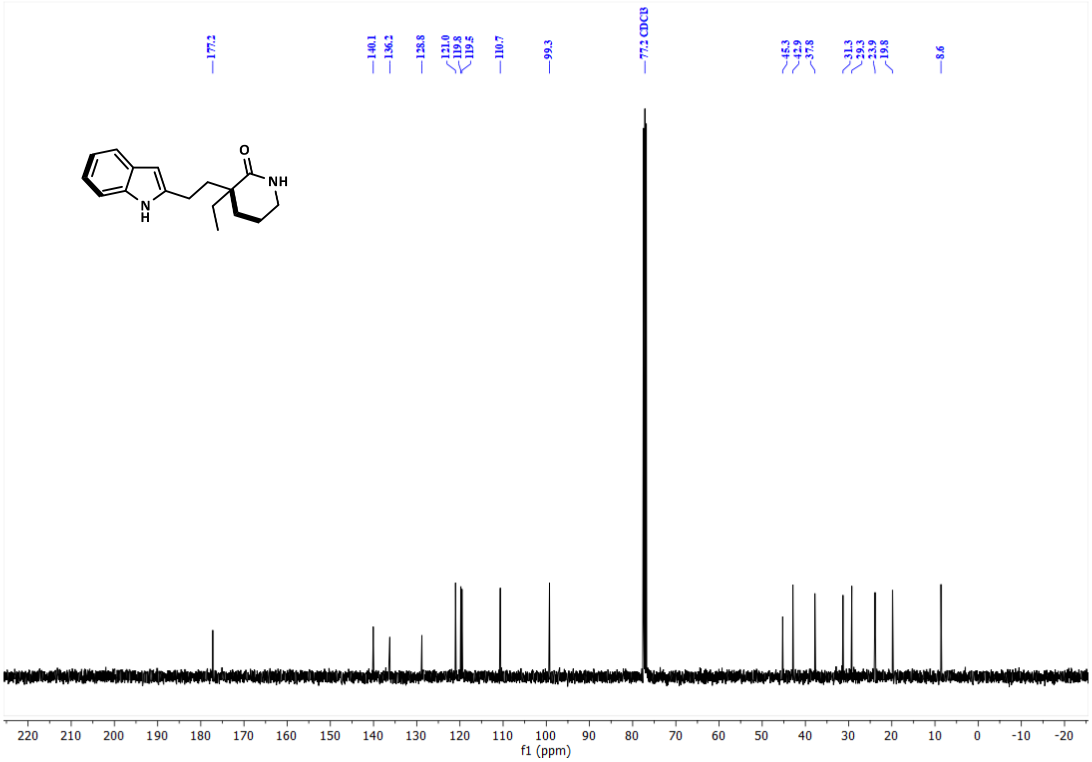

*Compound **38**. ^{1}H NMR (400 MHz, CHLOROFORM-D).*

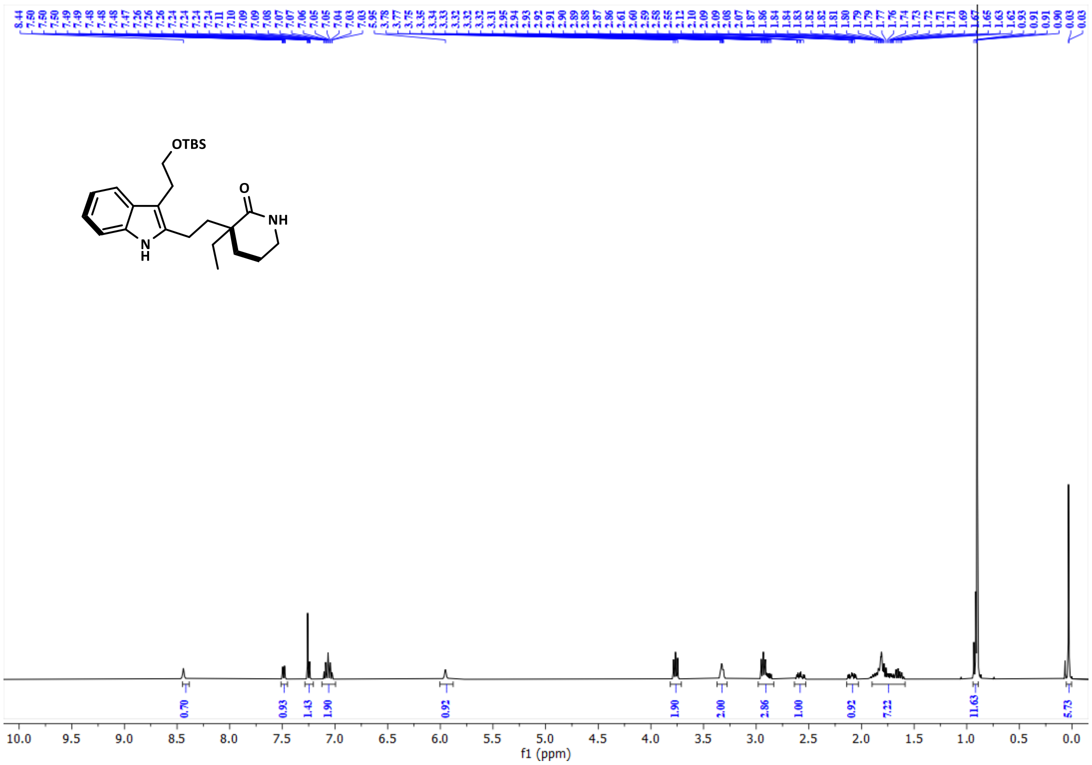

*Compound **38**. ^{13}C NMR (100 MHz, CHLOROFORM-D).*

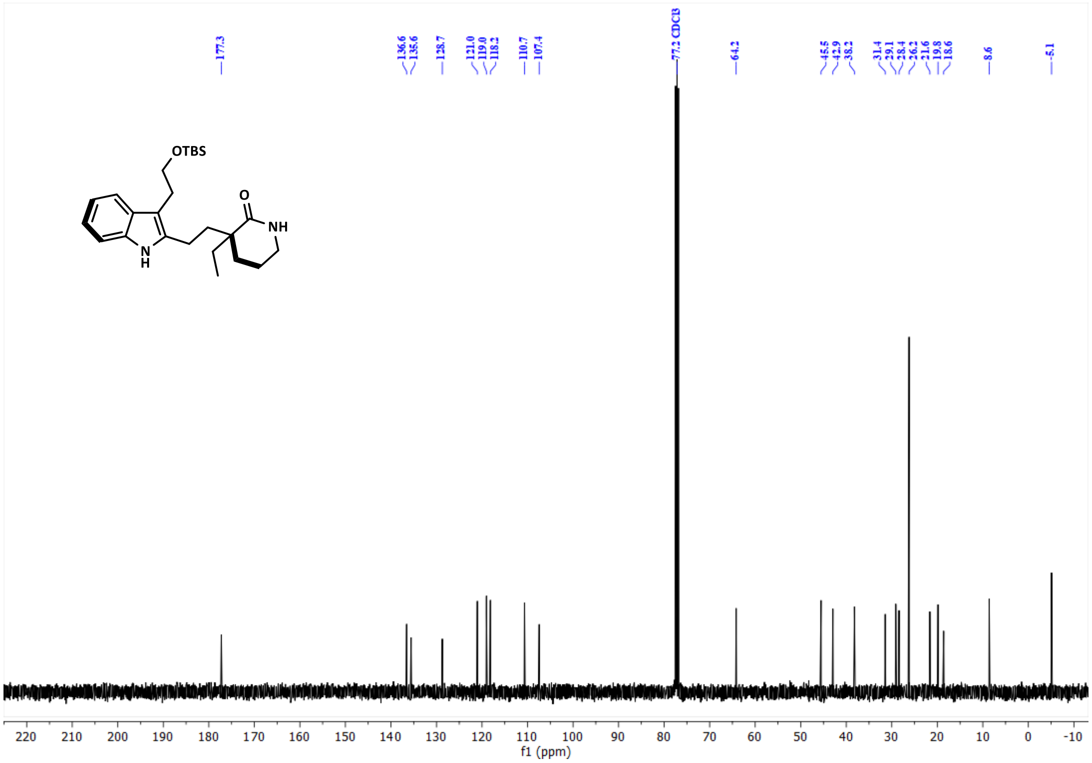

*Compound **39**. ^{1}H NMR (400 MHz, CHLOROFORM-D).*

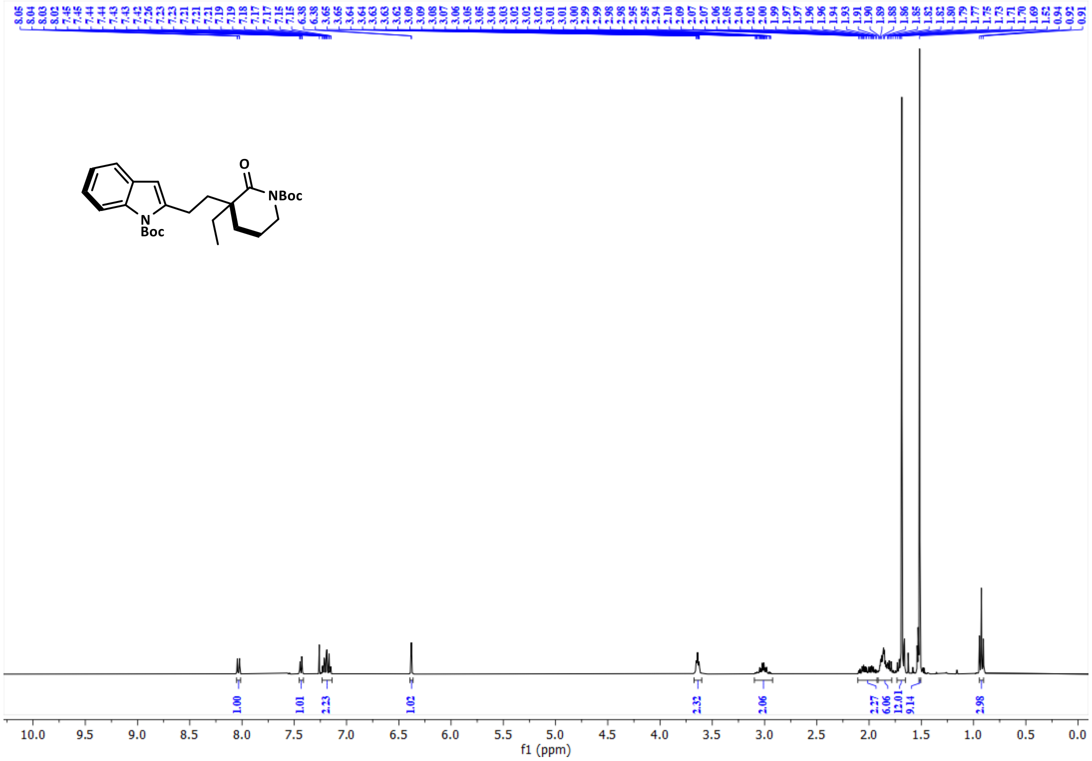

*Compound **39**. ^{13}C NMR (100 MHz, CHLOROFORM-D).*

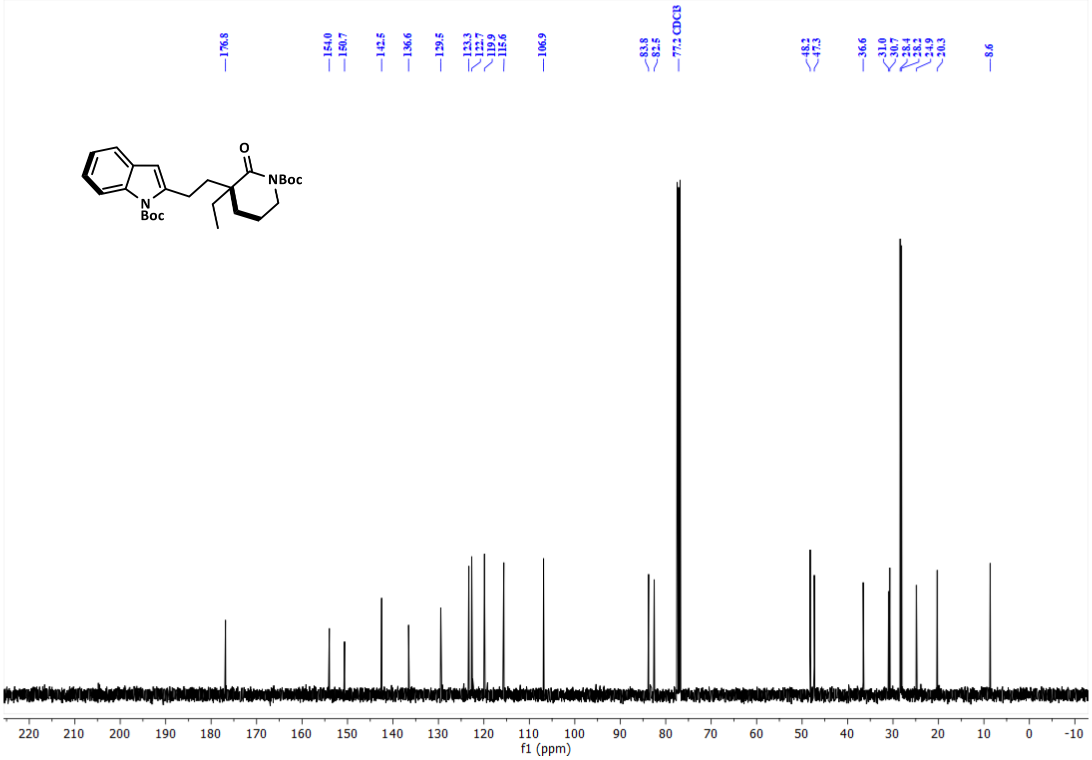

*Compound **40**. ¹H NMR (400 MHz, CHLOROFORM-D).*

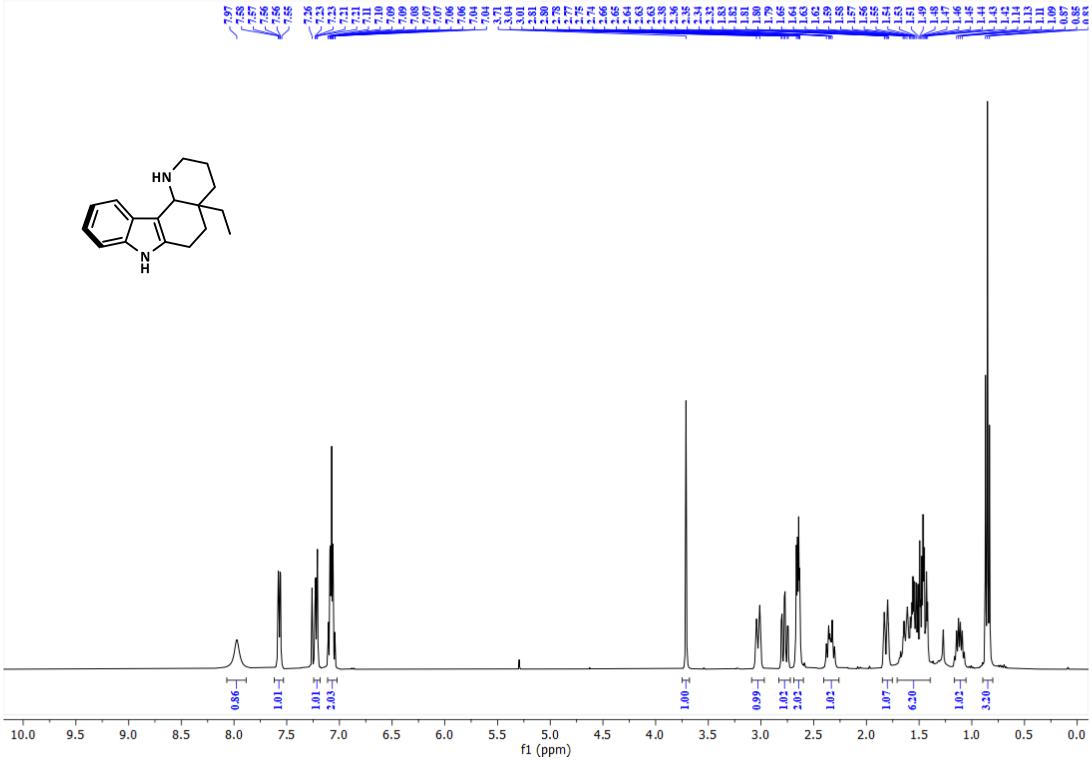

*Compound **40**. ¹³C NMR (100 MHz, CHLOROFORM-D).*

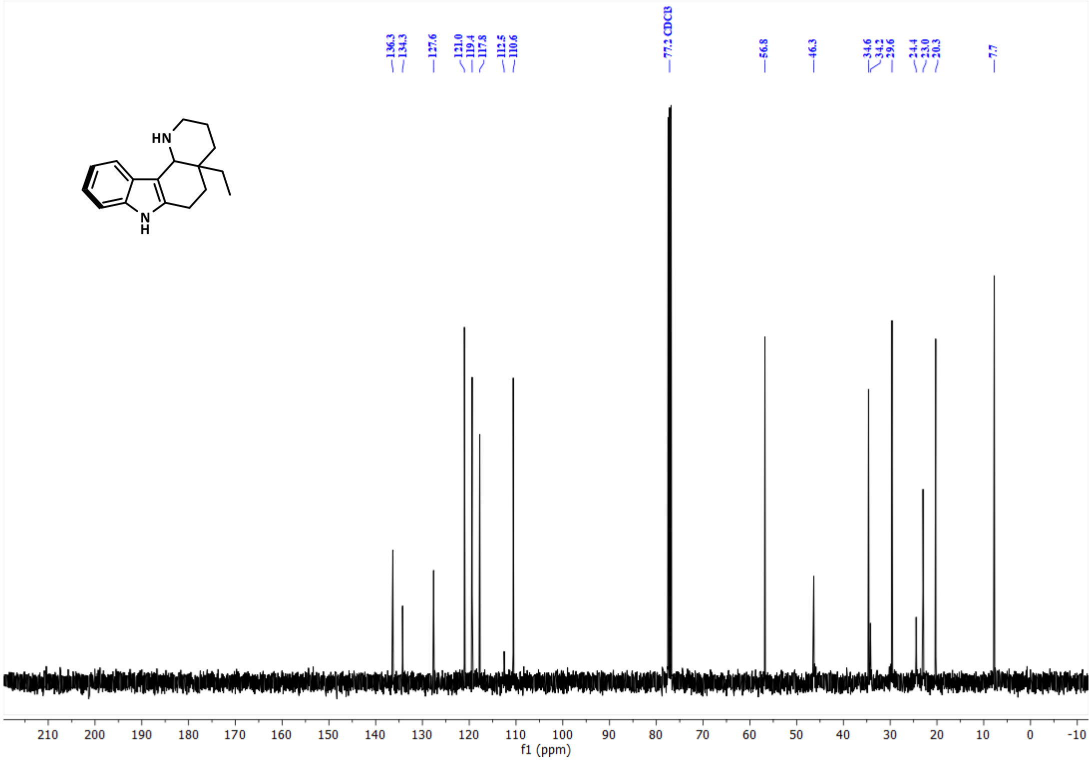

*<u>Compound **41**. ^{1}H NMR (400 MHz, CHLOROFORM-D).</u>*

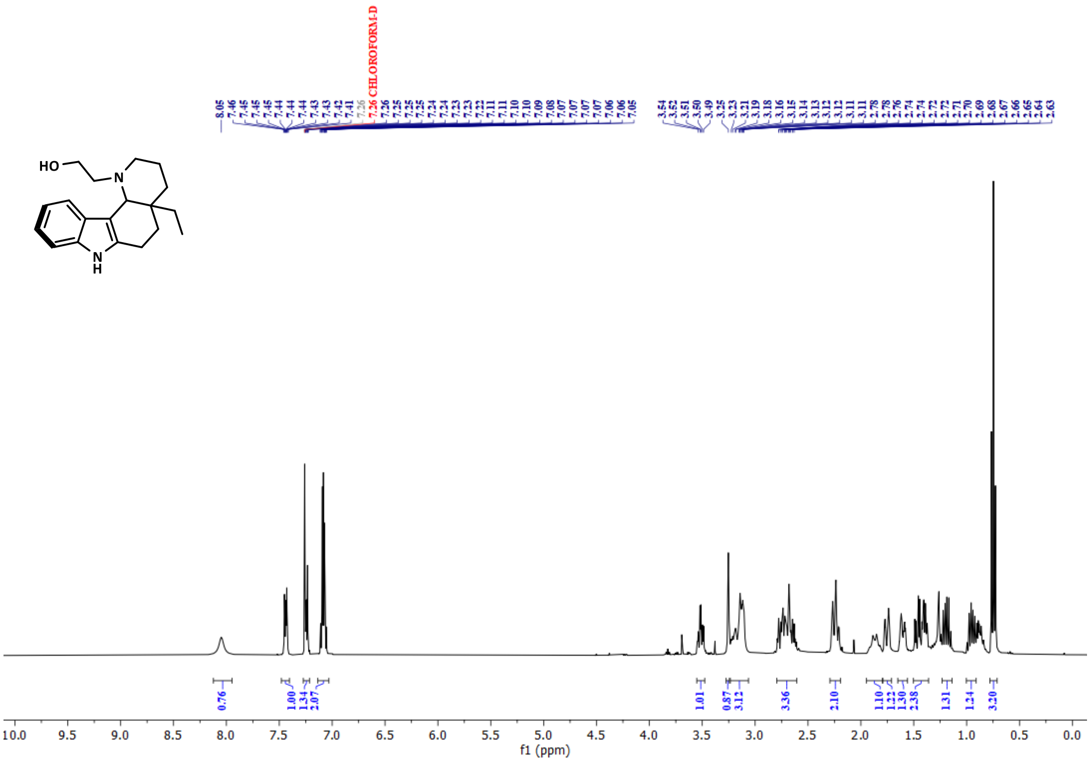

*<u>Compound **41**.</u> <u>^{13}C NMR (100 MHz, CHLOROFORM-D).</u>*

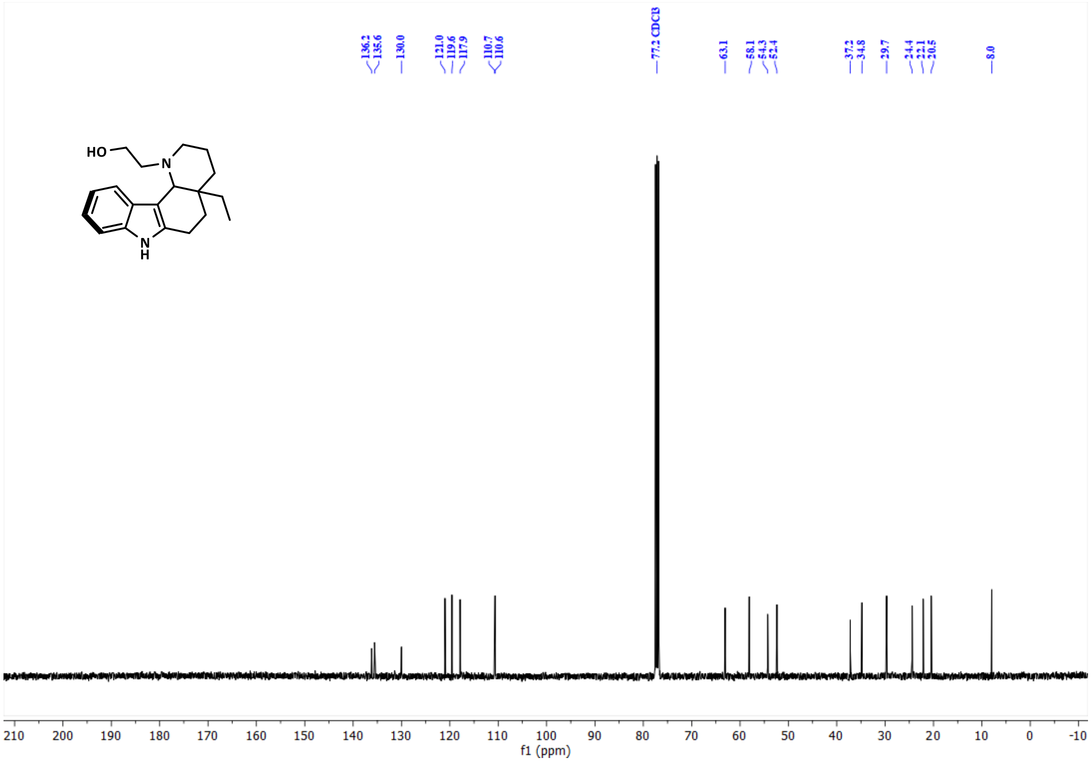

*Compound **42**. ^{1}H NMR (400 MHz, CHLOROFORM-D).*

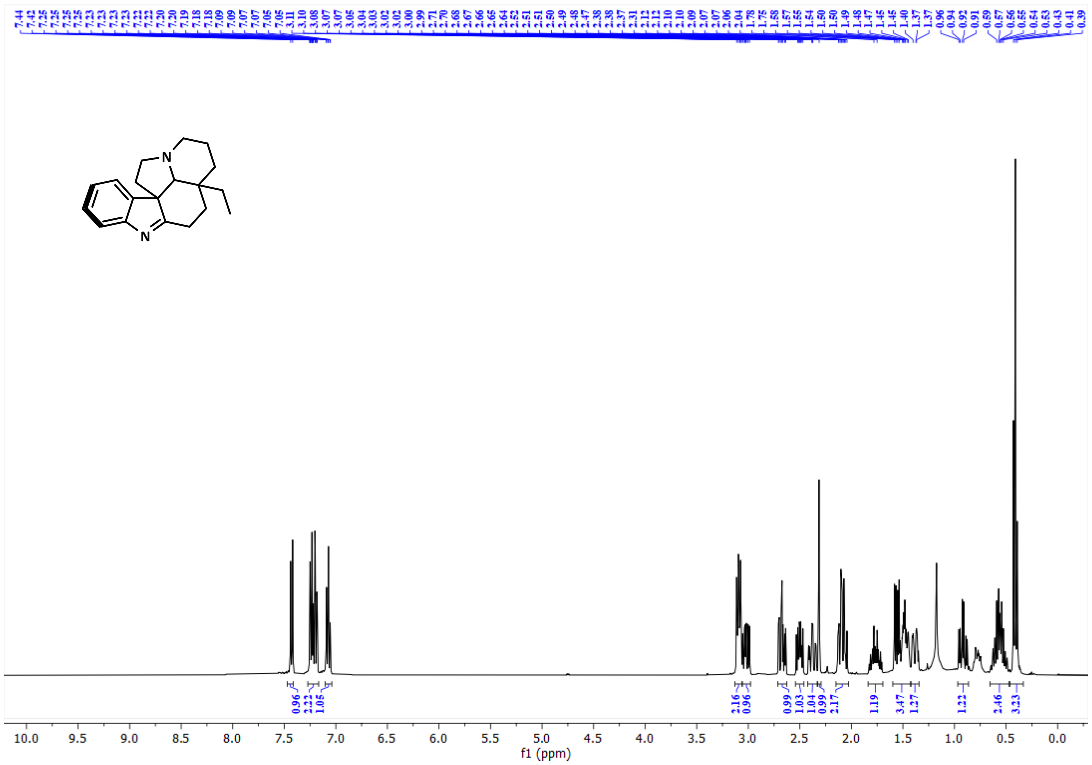

*Compound **42**. ^{13}C NMR (100 MHz, CHLOROFORM-D).*

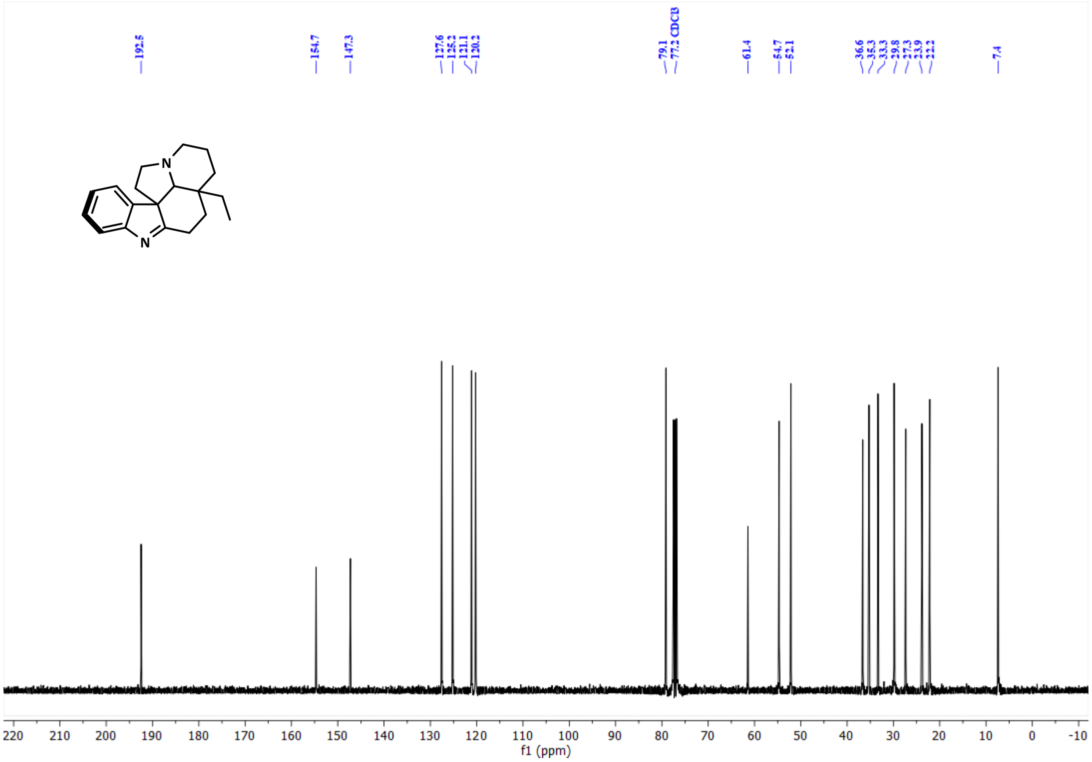

4. REFERENCES.

1 Y. Du, H. Y. Huang, H. Liu, Y. P. Ruan, P. Q. Huang, *Synlett* **2011**, *4*, 565–568.

2 L. Wang, X. Mei, C. Wang, W. Zhu, *Tetrahedron* **2015**, *71*, 7990–7997.

3 C. Empel, S. Jana, Ł. W. Ciszewski, K. Zawada, C. Pei, D. Gryko, R. M. Koenigs, *Chem. - A Eur. J.* **2023**, *29*, e202300214.
